# Spatial and temporal regulation of Wnt signaling pathway members in the development of butterfly wing patterns

**DOI:** 10.1126/sciadv.adg3877

**Published:** 2023-07-26

**Authors:** Tirtha Das Banerjee, Suriya Narayanan Murugesan, Heidi Connahs, Antόnia Monteiro

**Affiliations:** ^1^ Department of Biological Sciences, National University of Singapore, Singapore - 117557.; ^2^ Science Division, Yale-NUS College, Singapore - 138527.

## Abstract

Wnt signaling members are involved in the differentiation of cells associated with eyespot and band color patterns on the wings of butterflies, but the identity and spatio-temporal regulation of specific Wnt pathway members remains unclear. Here, we explore the localization and function of Armadillo/β-catenin dependent (canonical) and Armadillo/β-catenin independent (noncanonical) Wnt signaling in eyespot and band development in *Bicyclus anynana* by localizing Armadillo (Arm), the expression of all eight *Wnt* ligand and four *frizzled* receptor transcripts present in the genome of this species and testing the function of some of the ligands and receptors using CRISPR-Cas9. We show that distinct Wnt signaling pathways are essential for eyespot and band patterning in butterflies and are likely interacting to control their active domains.

## Teaser

Distinct Wnt signaling pathways interact to pattern the wings of butterflies.

## INTRODUCTION

Wnt signaling is fundamental to cellular communication in multicellular organisms. This communication involves secreted glycoproteins, the Wnt ligands, that are produced in signaling cells, traveling some distance away to regulate the expression of target genes in surrounding cells (*1*, *2*). Understanding the mechanisms underlying Wnt signaling is fundamental to studies of both normal and altered development, such as in wound healing and cancer, and has remained elemental in basic and applied biological research (*3*–*7*). There is, however, considerable debate about the spatial and temporal regulation and interaction of different Wnt signaling pathways; the distance and the mechanisms by which Wnts can travel across tissues; the mechanism by which distinct Wnt pathway members are regulated in cells; and how different Wnt ligands and receptors interact with each other (*8*–*10*).

In classical studies, there are two main pathways involved in Wnt signaling—a canonical and a noncanonical pathway—which use different ligand and receptor gene paralogs to transduce extracellular signals. In canonical Wnt signaling in *Drosophila* (fig. S1, A and B), specific Wnt ligands bind to specific Frizzled receptors at the cell surface, which then signal through Armadillo (Arm) (β-catenin in vertebrates) to regulate gene expression in the nucleus (*11*–*14*). Noncanonical Wnt signaling works independently of Arm/β-catenin and includes the planar cell polarity (PCP) pathway, which regulates the cytoskeleton of cells (fig. S1C), among other pathways (*15*–*18*). PCP signaling has been reported to work both in a Wnt ligand–dependent and in a Wnt ligand–independent manner in mouse and *Drosophila,* respectively (*15*, *19*, *20*). Both canonical and noncanonical pathways, however, are involved in similar processes of tissue organization, cell proliferation, and cell-cell communication (*2*, *9*, *21*–*23*).

The distinct Wnt ligands and Frizzled receptors used by Wnt pathways belong to multicopy gene families. In insect genomes, there are six to nine *Wnts* (*24*) and three to four *frizzled* genes (*25*), while in mammalian systems, there are 19 *Wnts* and 10 *frizzled* genes (*9*, *18*). Classically, *Wnts* such as *Wnt1*, *Wnt3*, *Wnt8*, and *Wnt10* have been associated with the canonical pathway, while *Wnt5a*, *Wnt7a*, and *Wnt11* with the noncanonical pathway in metazoans (*9*, *26*–*29*). Newer studies, however, have contradicted such categorization since some Wnts can transduce alternate Wnt signaling when coupled with specific receptors (*9*). Wnt5a, for example, can work both in the canonical pathway, using receptors such as *frizzled4*, and noncanonical pathway using *frizzled8* receptors (*28*, *30*). Here, we examine the diversity of Wnt signaling pathways involved in butterfly wing patterning, as these large epithelial surfaces, with their diverse and colorful wing patterns, are ideal canvases for exploring complex pattern formation processes via cell signaling.

Wnt signaling, using *Wnt1*, has previously been implicated in regulating butterfly eyespot size, but the involvement of Wnt signaling in differentiating eyespot centers, critical for color ring differentiation, has not been investigated. Multiple butterfly species have stable *Wnt1* expression along the wing margin throughout larval development (*31*–*34*), but the signal transducer of canonical Wnt signaling, Arm, displays a dynamic pattern of expression during the larval stages (*35*, *36*). Arm starts to be expressed across the whole wing but later resolves into a broad vein and marginal expression. This expression then progressively narrows on top of the veins, wing margin, and also along fingers parallel to and centered between wing veins. These fingers contain an enlarged cluster of Arm-expressing cells in the middle, the eyespot foci, that mark the future eyespot centers (*35*, *36*). Later in development, during the early pupal stage, *Wnt1* is expressed in these focal cells suggesting a role in signaling from these cells to pattern the rings of color around the focus (*32*, *37*). Transgenic RNA interference studies against *Wnt1*, where *Wnt1* was down-regulated at the end of the pre-pupal stage and beginning of the pupal stage, resulted in the reduction of all the colored rings, indicating that *Wnt1* regulates eyespot size (*37*). It is, however, still unclear whether Arm or Wnt signaling is required for eyespot center differentiation in the larval stages. Furthermore, the identity of the Wnt ligand(s) leading to Arm nuclear localization in the eyespot center during larval wing development is currently unknown.

WntA is another Wnt ligand that has been linked to the development and identity of cells involved in distinct color pattern elements including bands, patches, and eyespots in butterfly wings. Linkage mapping studies first identified WntA as an important prepatterning gene in *Heliconius* butterflies (*38*). Variation in WntA expression in different Nymphalid butterflies appears to correspond to the complex array of banding patterns laid out in the Nymphalid groundplan (*39*, *40*). In a generalized butterfly wing pattern, *WntA* is expressed in a central band [of the central symmetry system (CSS)], a more proximal band [of the basal symmetry system (BSS)], and along a marginal band system (MBS) in the larval wings of some Nymphalid butterflies including *Junonia coenia*, *Pararge aegeria*, *Vanessa cardui*, *Agraulis vanilla*, and *Heliconius* butterflies (*34*, *38*, *41*). *WntA* is also expressed in the silver color patches/spots in *A. vanilla* and in the ventral forewing eyespots of *V. cardui* (*34*, *41*). Knockout of *WntA* resulted in phenotypes with loss of pigmentation and reduction in the forewing eyespots of *V. cardui* (*41*). WntA has been shown to control the domain over which *Optix* is expressed, where knockout of *WntA* results in the expansion of *Optix* in *Heliconius erato* (*42*). In *Bicyclus anynana*, no study has yet explored the expression and function of this ligand.

In the present work, we explored the spatial and temporal expression of different Wnt signaling pathway genes in the wings of *B. anynana*, containing both eyespots and banding patterns. We first focused on the localization of all the *Wnt* ligands, *frizzled* receptors, Arm signal transducer, and the known Wnt target genes in *Drosophila melanogaster*, *Distal-less* (*Dll*) and *vestigial* (*vg*) in larval and pupal wings, and then we tested the function of four of these genes, *arm*, *WntA*, *Wnt7*, and *frizzled4*, with CRISPR-Cas9.

## RESULTS

### Phylogenetic analysis of *Wnt* and *frizzled* genes of *B. anynana* cluster with members of the same gene family

To discover all possible *Wnt* and *frizzled* genes present in the *B. anynana* genome, we searched for the corresponding gene annotations in National Center for Biotechnology Information [NCBI; *B. anynana* (taxid:110368)] and also blasted the orthologous *Wnt* and *frizzled* sequences from *D. melanogaster* in FlyBase against the *B. anynana* genome nBa.0.1, v1.2 and v2.0 in Lepbase (*43*–*45*). We identified eight *Wnts* and four *frizzled* genes in the genome. *Wnt9* was only found in nBa.0.1 but not v1.2 and v2.0 of the *B. anynana* genome (*46*). A phylogenetic analysis performed with these genes and orthologs from other insects showed that the eight *Wnt*s represent *Wnt1*, *Wnt5*, *Wnt6*, *Wnt7*, *Wnt9*, *Wnt10*, *Wnt11*, and *WntA*, who cluster with members of the same gene family from other butterfly and insect species (figs. S2 and S3). Similarly, a separate phylogenetic analysis identified *frizzled*, *frizzled2*, *frizzled4*, and *frizzled9* as being the four *frizzled* genes in the *B. anynana* genome (figs. S4 and S5).

### Arm is expressed in a dynamic pattern in larval and pupal wings

**Fig. 1.**
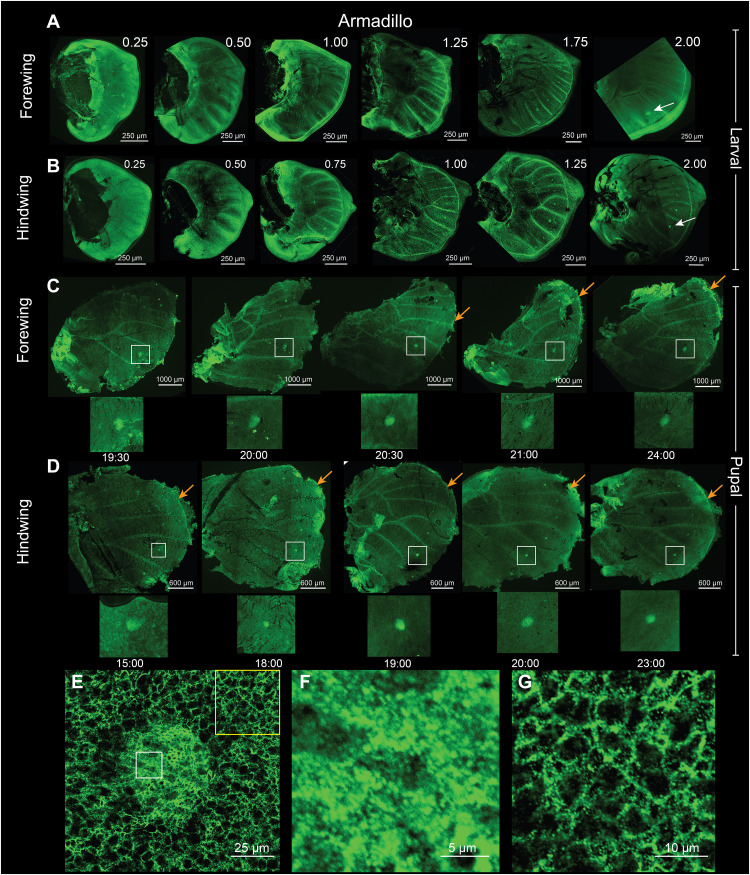
Localization of Arm in *B. anynana*. (**A** and **B**) Arm is present homogeneously throughout the larval forewing and hindwing tissue till stage 0.50. At stages 0.50 to 0.75, accumulation in the eyespot foci is observed that continues throughout the larval stage together with expression along the wing margin and the wing veins (shown here till stage 2.00); for stage nomenclature, please refer to (*36*). Scale bars, 250 μm. (**C** and **D**) During pupal forewing and hindwing development, Arm is observed in the foci from 15 to 24 hours after pupation (AP). (**E** and **F**) Arm is localized in the cytoplasm and in the nuclei of focal cells. (**G**) Outside the foci, Arm is present mostly at the cell membrane.

Previous gene expression/accumulation, transcriptomic, and modeling studies have proposed a role for Arm in the differentiation of the eyespot foci (*35*, *36*, *47*). To extend these findings, we used immunostainings to document Arm’s spatial-temporal expression across both larval and pupal wings at multiple time points and across a longer period of wing development than previously investigated. In early fifth instar larvae, Arm was homogeneously distributed across the wing (stage 0.25; Fig. 1, A and B). As the wing developed, the protein was accumulated along the vein cells, wing margin, and at the eyespot foci (stages 0.50 to 2.00; Fig. 1, A and B). During the pupal stage, Arm protein was observed in the eyespot foci and along the wing margin from 15 to 24 hours after pupation (AP) (Fig. 1, C and D). We lack data from the early hours AP (before 15 hours AP) because wings are too fragile to handle before this stage. Arm protein was also observed in the foci in three other nymphalid butterflies during mid-late larval wing development along with the eyespot marker protein Spalt (fig. S6). We conclude that Arm protein, and canonical Wnt signaling, is likely continuously present in the eyespot foci from mid fifth instar larval development to at least 24 hours AP. We observed cytoplasmic Arm in the eyespot center, while in the surrounding cells, Arm was present mostly in the cell membrane (Fig. 1, E to G).

Knockout of *arm* using CRISPR-cas9 resulted in mosaic crispants that showed either a complete loss of the eyespot (one individual) (fig. S7, C and D) or split eyespots (two individuals), where two foci differentiated side-by-side within a sector bordered by veins (fig. S7, E to H). Defects along the wing margin were also observed in four of the *arm* CRISPR individuals (fig. S7, C to J). We verified that the double foci phenotypes were not due to ectopic veins running through the middle of the pattern (fig. S7, K and L). Ectopic venation, however, was observed in *arm* CRISPR individuals (fig. S7N). The CRISPR phenotypes were verified by Illumina paired-end sequencing where indels were observed at the CRISPR-Cas9 target site in the affected tissues (fig. S7, O and P).

### *Wnt1*, *Wnt6*, and *Wnt10* are expressed in larval and pupal wings

**Fig. 2.**
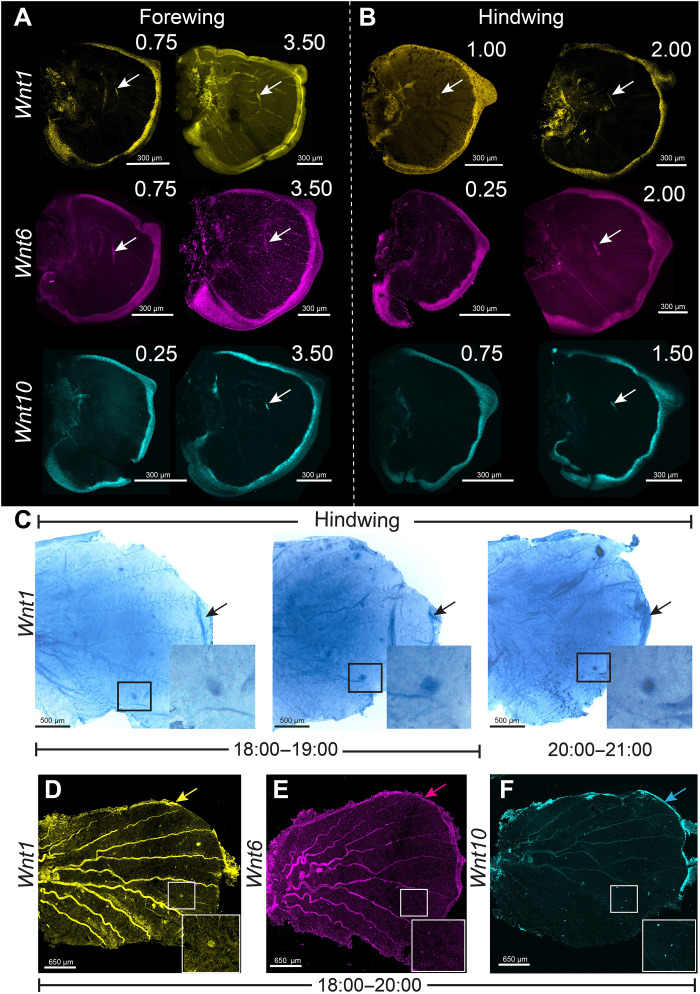
Expression of canonical *Wnt* ligands using HCR3.0 and in situ hybridization in larval and pupal wings. (**A** and **B**) The mRNA expression of three canonical *Wnts*, *Wnt1*, *Wnt6*, and *Wnt10*, in the forewing and hindwing is limited to the wing margin and there is no focal expression. Expression is observed at the discal spot, for all three genes, during larval wing development (white arrows). Expression of (**C** and **D**) *Wnt1* transcripts in the eyespot foci and in the wing margin (black/yellow arrows) and (**E**) *Wnt6* and (**F**) *Wnt10* in the wing margin (magenta and cyan arrow) from 18 to 21 hours of pupal wing development. Note the autofluorescence in the tracheal tissue running along the wing veins in some wings are not mRNA signals.

To localize known canonical *Wnt* transcripts on the wing that could be responsible for the nuclear translocation of Arm in the eyespot focal cells (Fig. 1, E and F), we used hybridization chain reaction [Hybridization Chain Reaction v3.0 (HCR3.0)] (*48*). *Wnt1*, *Wnt6*, and *Wnt10* were all expressed along the wing margin during larval and pupal development (Fig. 2 and figs. S8 and S9), with no specific expression in the eyespot foci during the larval stages tested (Fig. 2, A and B, and fig. S8). In 18 to 24 hours pupal wings, however, we confirmed the expression of *Wnt1* transcripts in the eyespot foci, as well as expression along the wing margin using both chromogenic in situ hybridizations and HCR3.0 (Fig. 2, C and D, and fig. S9, A to F) (*37*), whereas *Wnt6* and *Wnt10* were only expressed along the wing margin (Fig. 2, E and F, and fig. S9, B, E, and M). During the pupal stage, the nuclear presence of Arm in the foci is likely driven by the locally transcribed *Wnt1*. However, in the larval stage, nuclear Arm at the foci is probably driven by *Wnt1*, *Wnt6*, and *Wnt10* produced along the wing margin, some distance away, and reaching the focal cells via some form of diffusion (or other form of transport), as previously modeled (*35*). Expression of all the three *Wnts* was also observed in the discal spot, on top of a future cross-vein (Fig. 2, A and B, and fig. S8), consistent with previous studies in other butterflies (*33*, *34*).

### *Dll* and *vg* are expressed in larval wings and *Dll* also in pupal wings

**Fig. 3.**
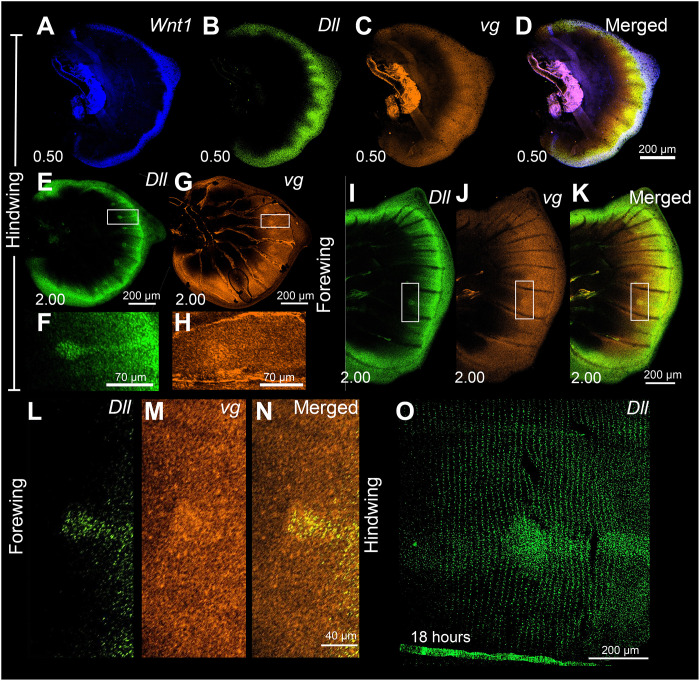
Expression of *Wnt1, Dll,* and *vg* in larval wings. (**A**) *Wnt1* is expressed along the wing margin. (**B**) *Dll* expression is observed in some cells away from the wing margin, while (**C**) *vg* is expressed in a broader domain. (**D**) Merged expression of *Wnt1*, *Dll*, and *vg* showing the range of a potential Wnt glycoprotein gradient activating its potential target genes. (**E** and **F**) Expression of *Dll* in an older larval wing (stage 2.00) in the foci and in the fingers from the wing margin. (**G** and **H**) Expression of *vg* showing a broader domain of expression both along the wing margin and in the foci. (**I** to **N**) Coexpression of *Dll* and *vg* in the larval forewing showing expression in the eyespot center in a smaller domain for *Dll* and in a bigger domain for *vg*. (**O**) Expression of *Dll* in an 18-hour pupal wing showing expression in the future black scale cells.

To investigate the domain over which Wnt1 glycoproteins, along with Wnt6 and Wnt10, might be activating target genes, we examined the coexpression of *Wnt1* and two known targets of Wnt signaling in *Drosophila*, *Dll* and *vg*, in larval and pupal wings of *B. anynana*. In the *Drosophila* wing, disc Wnt1 is secreted from the wing margin and travels into more interior wing regions where it activates *Dll* and *vg* at two different concentration thresholds (*1*). In the early larval wings of butterflies, we observed the expression of *Dll* in broad finger-like projections some cells away from the wing margin (Fig. 3B), while *vg* is expressed in a broader domain (Fig. 3C), consistent with *Drosophila* data (*1*). At a later larval stage (2.00), we observed *Dll* expression in the focal cells (Fig. 3, E, F, and I), while *vg* was up-regulated in a slightly broader cluster of focal cells (Fig. 3, G, H, and J). *Wnt1*, however, was still not expressed in the foci at this stage (Fig. 2, A and B, and fig. S8, A to H). No expression of *vg* was observed in 18 to 24 hours pupal wings (fig. S11).

### *Wnt5*, *Wnt7*, *Wnt9*, and *Wnt11* are not expressed in the stages tested during larval and pupal wing development

**Fig. 4.**
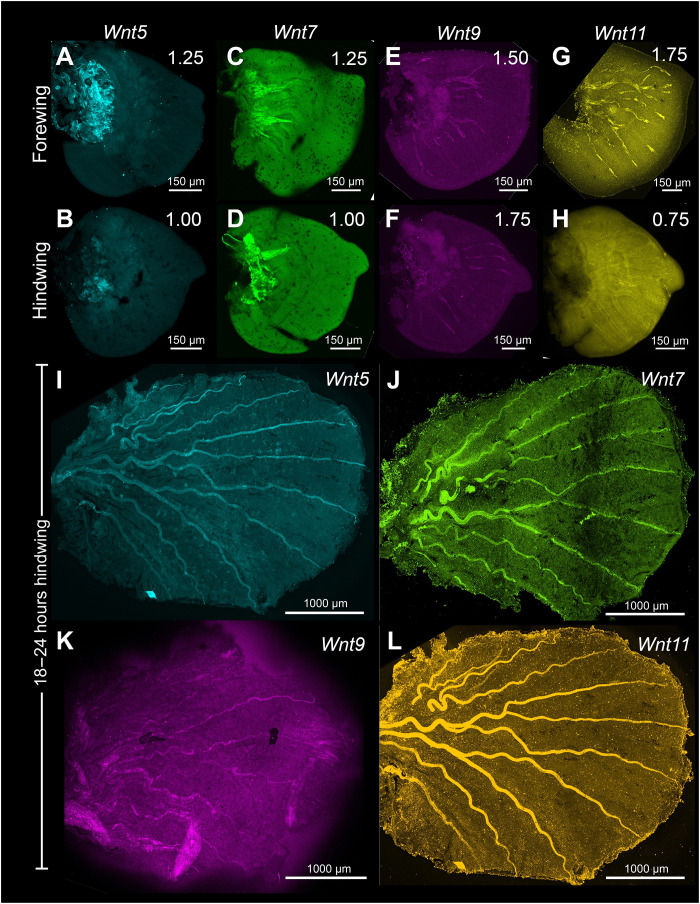
Expression of Wnt ligands *Wnt5*, *Wnt7*, *Wnt9*, and *Wnt11* in the larval and pupal wings. No specific expression domain was observed for (**A**, **B**, and **I**) *Wnt5*, (**C**, **D**, and **J**) *Wnt7*, (**E**, **F**, and **K**) *Wnt9*, and (**G**, **H**, and **L**) *Wnt11* during the stages tested in the larval and 18 to 24 hours pupal wing development in *B. anynana*.

To test whether *Wnt5*, *Wnt7*, *Wnt9*, and *Wnt11* typically associated with noncanonical Wnt signaling could play a role in *B. anynana* wing pattern formation, we also examined their expression in larval and pupal wings. None of the genes showed any specific expression domain in the larval and pupal wings at the stages tested (Fig. 4 and fig. S12). However, knockouts of *Wnt7* using CRISPR-Cas9 resulted in ectopic veins and ectopic eyespots differentiating in the wing sectors in 12 individuals (fig. S15, A to D). Because ectopic veins often lead to the creation of additional wing sectors with eyespots, we cannot directly implicate *Wnt7* in eyespot development. This gene appears to play a role, however, in vein development.

### *WntA* is involved in patterning the wings during larval and pupal wing development

**Fig. 5.**
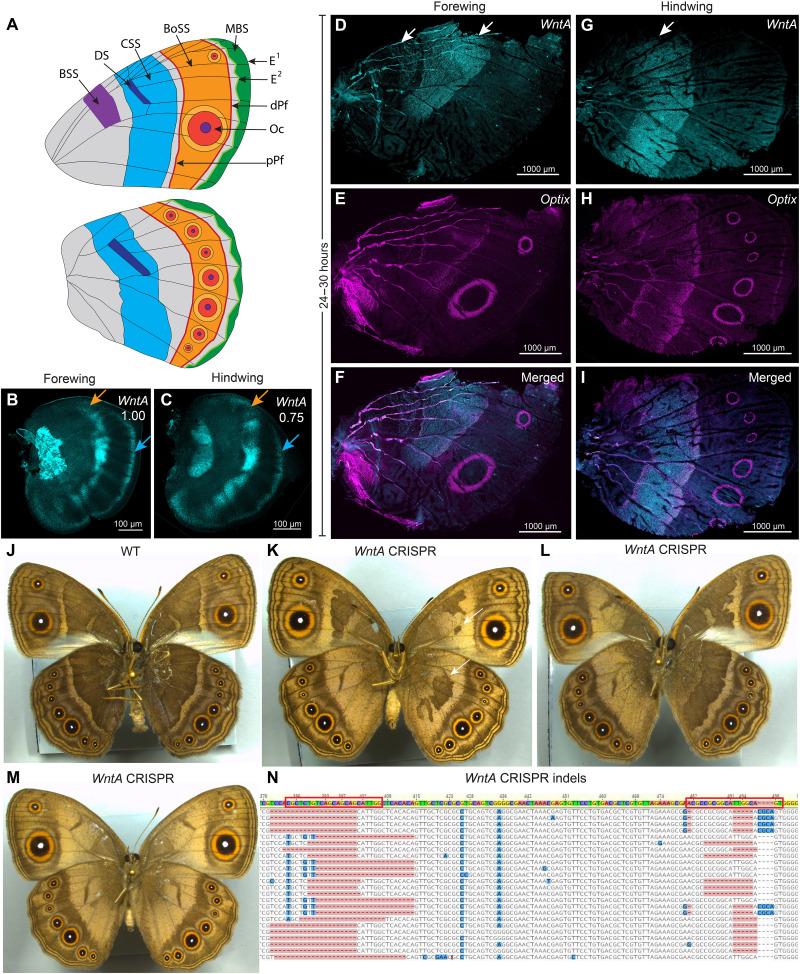
Expression of *WntA* and *Optix* in larval and pupal wings and function of *WntA* in the CCS and BSS. (**A**) An illustration of the nymphalid groundplan as applied to *B. anynana*. DS, Discal Spot; BoSS, Bordel Ocelli Symmetry System; Oc, border Ocelli (eyespot); dPf, distal Parafocal element; pPf, proximal Parafocal element; E^1^ and E^2^, marginal Externae patterns. (**B** and **C**) In larval wings, *WntA* is expressed along the CSS (orange arrow) and in the MBS (blue arrow). (**D** to **I**) Expression of *WntA* and *Optix* in 24 to 30 hours pupal (D to F) forewing and (G to I) hindwing. *WntA* shows expression along the bands of the CSS and BSS (white arrows) in forewings and in one CSS band (white arrow) in hindwings. *Optix* was expressed in cells at the boundary of *WntA* domains along with expression in the eyespot orange scale cells. (**J**) Wild-type (WT) adult. (**K** to **M**) *WntA* crispants show two types of phenotype: loss of scale cell identity along the CSS in both forewings and hindwings, in the BSS band in forewings, and in marginal chevrons, and reduced width and shape changes in the CSS (white arrows). (**N**) Indels in the *WntA* target sites (red boxes) were obtained via Illumina sequencing. *Optix* was used as a positive staining control. A total of 28 individuals with *wntA*-dependent defects were observed.

We next tested the expression of the last Wnt ligand, *WntA*, in both the larval and pupal wings of *B. anynana*. In larval wings, *WntA* was expressed along the CSS and along the MBS (Fig. 5, B and C, and fig. S10A). In the pupal stage, strong expression of *WntA* was observed in two bands along the BSS and along the CSS in forewings and one band along the CSS in hindwings (Fig. 5, D to I, and fig. S10F). These results are consistent with studies in other butterfly species (*41*). No expression was observed in the eyespot region in larval and pupal wings (Fig. 5, B to I, and fig. S10). We used *Optix*, a known transcription factor in butterfly wing patterning (*49*), as a positive control spanning the boundary of *WntA* expression in the CSS (Fig. 5, D to I, and fig. S10, E to G).

Functional disruption of *WntA* using CRISPR-cas9 resulted in the loss of brown color with ectopic orange scales appearing along the CSS and in the BSS (Fig. 5, J to M) and in changes in the width and outer shape of the CSS in 28 individuals (Fig. 5, K and L). No defects in the eyespot pattern were obtained in any individual (Fig. 5, K to M), but the light-colored marginal chevrons of the wing were disrupted (compare left and right hindwings in Fig. 5K). We confirmed indels at the targeted sites using Illumina sequencing from the affected adult wings (Fig. 5N).

### Expressions of *frizzled4* and *frizzled9* in larval wings are anti-colocalized

**Fig. 6.**
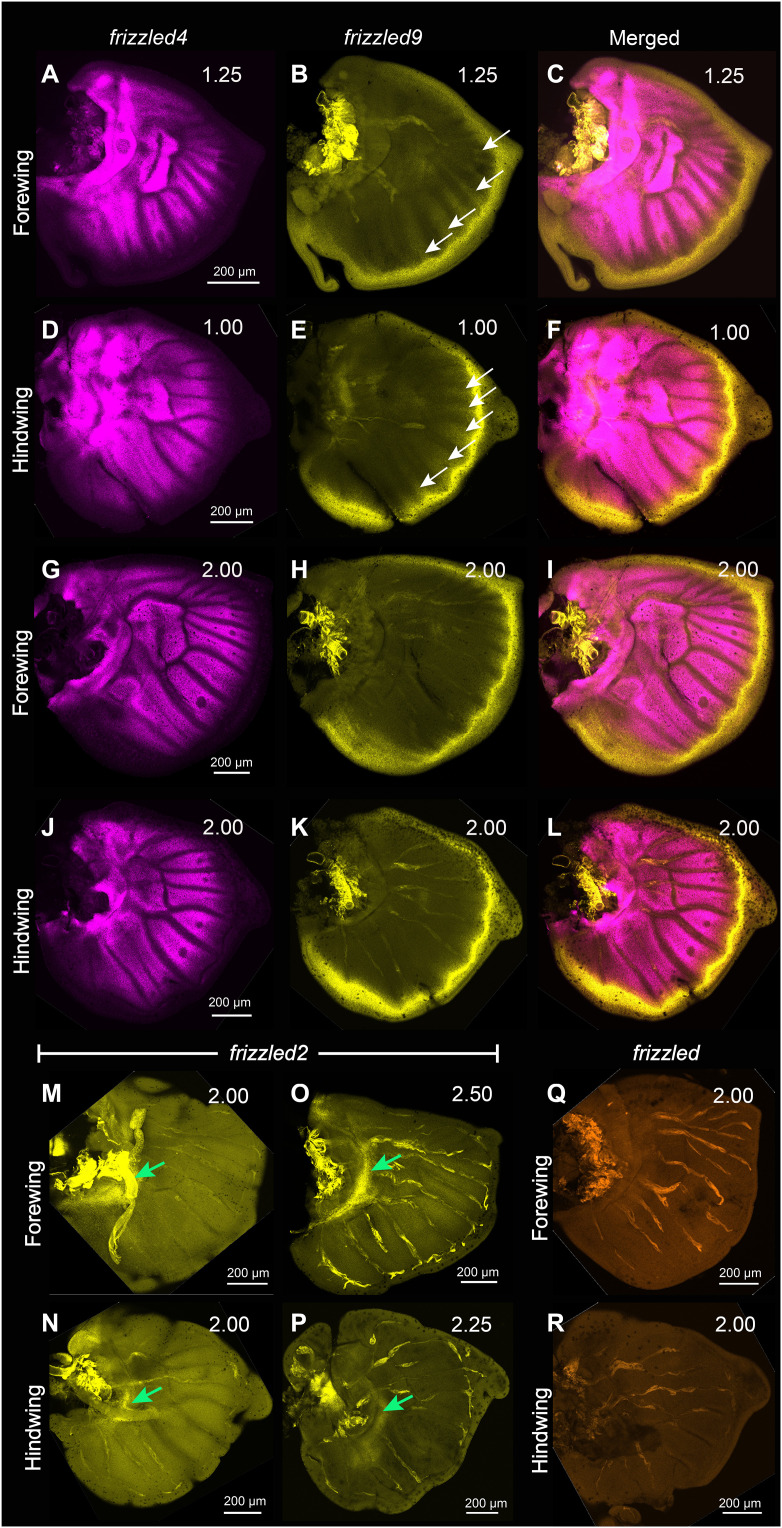
Expression of *frizzled4* and *frizzled9* in the larval wings of *B. anynana*. Expression of *frizzled4* and *frizzled9* in early larval (**A** to **C**) forewings and (**D** to **F**) hindwings. Expression of *frizzled4* and *frizzled9* in late larval (**G** to **I**) forewings and (**J** to **L**) hindwings. Expression of *frizzled2* in (**M** to **P**) larval wings along a proximal domain (green arrows). (**Q** and **R**) There is no clear expression of *frizzled* in larval forewings and hindwings.

We next aimed to identify the Frizzled receptor(s) that might be used for Wnt signaling in larval wings. We first examined the expression of *frizzled4* and *frizzled9* transcripts in larval wings using HCR3.0. In early stages (0.75 to 1.0), *frizzled4* was expressed homogeneously in the intervein cells (Fig. 6, A and D, and fig. S13, A and B), but at later stages (1.25 to 2.0), the expression was down-regulated in the eyespot foci and finger projections from the wing margin (Fig. 6, G and J, and fig. S13, C to H). The expression of *frizzled9* appeared complementary to that of *frizzled4* (Fig. 6, C, F, I, and L), with an early strong expression along the wing margin and in the finger-like projections at stages 0.75 to 1.25 (Fig. 6, B and E, and fig. S13, I and J), that became restricted to the wing margin at later stages (stage 2.00) (Fig. 6, H and K, and fig. S13, K to P). *frizzled4* also had lower expression in the lower posterior domain of late larval forewings where *frizzled9* had higher expression (Fig. 6, G to I).

### *Frizzled2* is expressed in a proximal domain while no specific domain of *frizzled* is observed in larval wings

We performed HCR3.0 on two other potential receptors, *frizzled2* and *frizzled*, in both larval and pupal wings. In the larval stage, the expression of *frizzled2* was restricted to the proximal domain of both fore and hindwings (Fig. 6, M to P). *frizzled* showed no specific expression in larval wings consistent with a previous study (*50*) (Fig. 6, Q and R, and fig. S13, Q to T) but was expressed in elevated levels in the eyespot foci in pupal wings at 18 to 24 hours AP (fig. S13, U to X).

### *frizzled2* is anti-colocalized with *frizzled4* in the eyespot field, with *frizzled9* in the pupal wing margin, and with *WntA* in the CSS band

**Fig. 7.**
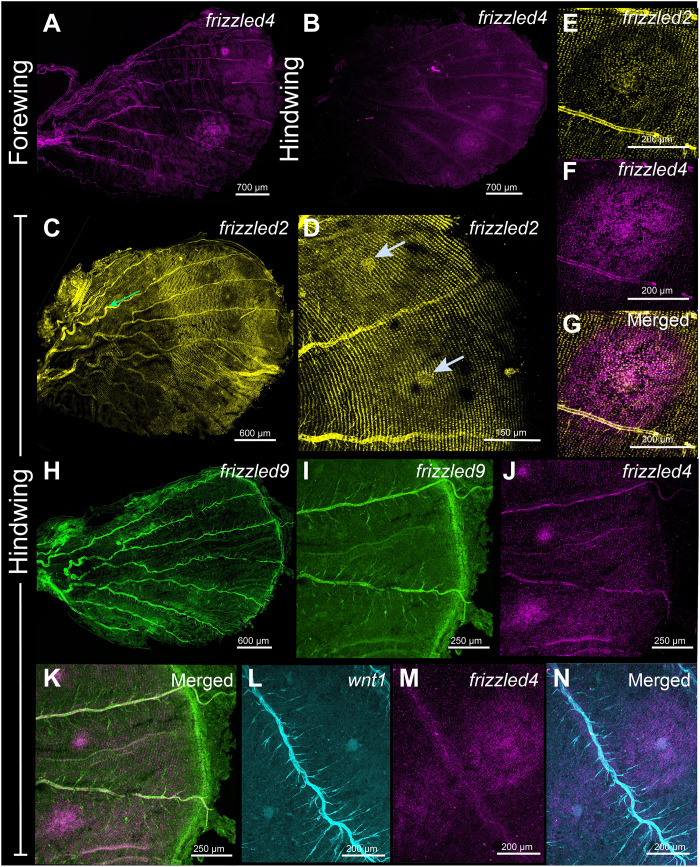
Expression of *frizzled2*, *frizzled4*, *frizzled9*, and *Wnt1* in 18 to 24 hours pupal wings of *B. anynana*. Expression of *frizzled4* in the eyespot field of pupal (**A**) forewings and (**B**) hindwings. (**C**) *frizzled2* is expressed in a proximal (green arrow) and a distal domain relative to the CSS. In the distal domain, *frizzled2* has reduced expression in the eyespot field, and especially in the cells of the orange ring (**D**), but is present in the foci. Expression of (**E**) *frizzled2*, (**F**) *frizzled4*, and (**G**) merged channel in the forewing Cu1 eyespot. *frizzled2* is lowly expressed in the domain where *frizzled4* is expressed. Pupal (**H**) hindwing showing the expression of *frizzled9* in the wing margin. Coexpression of (**I**) *frizzled9*, (**J**) *frizzled4*, and (**K**) merged. Expression of (**L**) *Wnt1*, (**M**) *frizzled4*, and (**N**) merged expression in the hindwing.

Next, we investigated the 18 to 24 hours pupal wing expression of *frizzled2*, *frizzled4*, and *frizzled9*. During the pupal stage, *frizzled4* was expressed in the eyespot foci and in domains spanning the eyespot rings, bearing little resemblance to its early larval patterns (Fig. 7, A and B, and fig. S14C). *frizzled2* was expressed in two large domains flanking each side of the CSS, where *WntA* was previously observed: a proximal band domain and a distal domain (Fig. 7C and figs. S10C and S14, A and B). In this distal domain, the expression of *frizzled2* was elevated in the eyespot foci but reduced in the areas of the future black and orange rings (Fig. 7, C and D, and fig. S10C). Coexpression of *frizzled2* and *frizzled4* showed the eyespot domain over which *frizzled4* was highly expressed had reduced levels of *frizzled2* (Fig. 7, E to G, and fig. S14, D to F). Lower levels of *frizzled2* and *frizzled4* were also observed along the wing margin, where *frizzled9* was strongly expressed (Fig. 7, D and H to K). The focal expression of *frizzled4* overlapped the expression of *Wnt1* (Fig. 7, L to N).

### *frizzled4* functions in eyespot center differentiation and in PCP

**Fig. 8.**
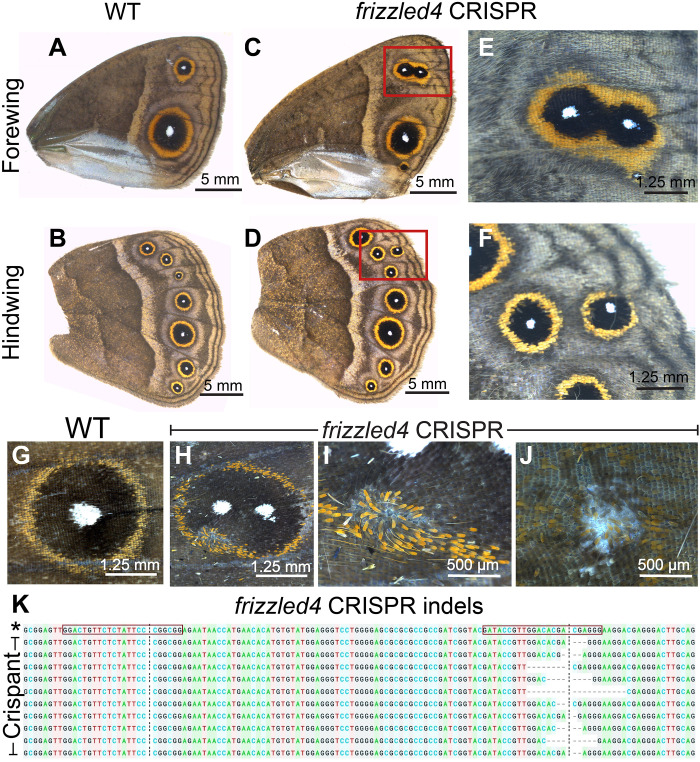
Function of *frizzled4* in eyespot center formation and PCP in *B. anynana.* (**A** and **B**) WT forewing and hindwing. (**C** to **F**) Some *frizzled4* crispants differentiated two eyespots in the same wing sector. (**G**) WT eyespot. (**H** to **J**) *frizzled4* crispants showed that *frizzled4* also plays a role in PCP affecting scale orientation in the eyespot field. (**K**) Sequences from *frizzled4* CRISPR individuals showing indels at the second CRISPR target site. Target sites are highlighted in red boxes. Red boxes indicate the two target sequences. A total of 18 individuals were observed with two eyespots in the same sector and 5 individuals with wing margin defects. “*” indicates the WT sequence.

To test the role of *frizzled4*, we knocked it out using CRISPR-Cas9 at the embryonic stage. In 18 individuals, mosaic crispant wings differentiated two eyespot centers along the proximal-distal axis (Fig. 8, C to F and H, and fig. S15, E to K). Two crispants also showed defects in the orientation of the orange and black scale cells in eyespots (Fig. 8, H to J). The knockout results were confirmed by sequencing the affected wing tissue (Fig. 8K).

## DISCUSSION

### Wnt signaling is involved in eyespot center differentiation in *B. anynana*

Our results suggest that canonical Wnt signaling is involved in the differentiation of the eyespot centers during the larval stage. At this stage, the canonical Wnt signaling transducer Arm is accumulated at high levels in the fingers and in the nuclei of cells in the eyespot centers, indicating the activation of Wnt signaling in those cells (Fig. 1, E and F). Disruptions of *arm* via CRISPR-cas9 resulted in either a split eyespot or no eyespots, suggesting *arm*’s essential role in eyespot center cell differentiation (fig. S7, C to H). *arm* knockouts also led to disruptions in the wing margin, suggesting its involvement in margin differentiation (fig. S7, C to L). The *Wnt* ligands *Wnt1*, *Wnt6*, and *Wnt10*, expressed along the wing margin throughout larval wing development (Fig. 2, A and B, and fig. S8), are likely leading to *arm* cytoplasmatic accumulation along the margin, in the fingers that project from the margin, and in the eyespot focus.

**Fig. 9.**
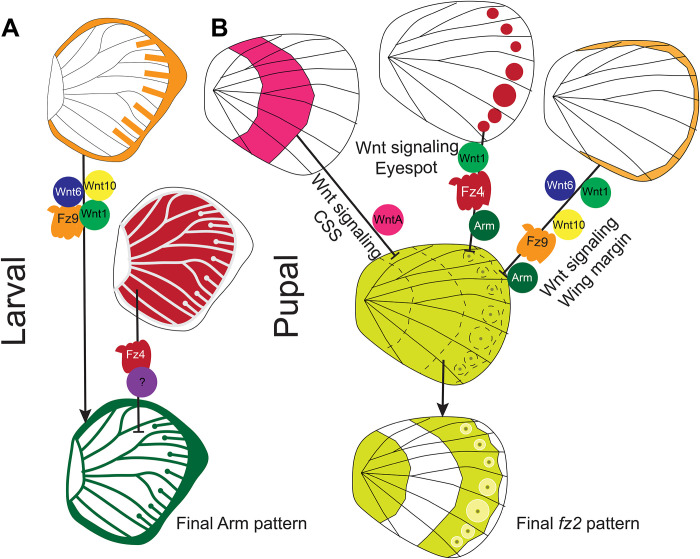
Models for the spatial control of Arm in the larval wings and *frizzled2* in the pupal wings. (**A**) In this model, Wnt signaling from the wing margin involving the ligands Wnt1, Wnt6, and Wnt10, along with receptor Frizzled9, activates Arm in the wing margin, intervein fingers, and eyespot centers. Frizzled4 expressed in the intervein cells and along the eyespot center represses the activity of Arm in those domain resulting in the pattern of Arm we observe in the larval wings. (**B**) In the second model, *frizzled2*, initially homogeneously expressed in the pupal wing tissue, becomes repressed in three distinct domains via Wnt signaling from the wing margin, from the eyespot central domain cells, and along the CSS.

**Fig. 10.**
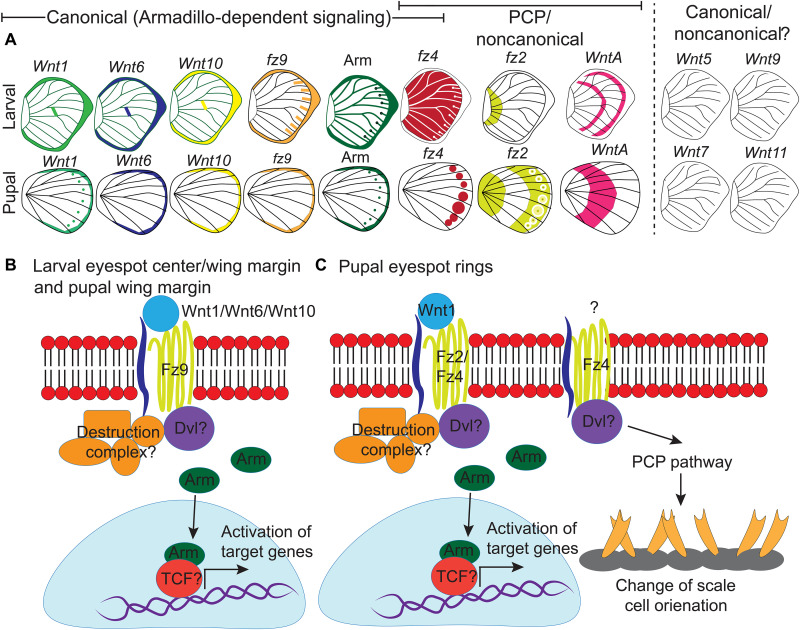
Expression of canonical and noncanonical Wnt ligands, receptors and signal transducer (Arm) in larval and pupal wings. (**A**) Expression patterns of *Wnt1*, *Wnt6*, *Wnt10*, and *WntA*; receptors *frizzled2 (fz2)*, *frizzled9* (*fz9)*, and *frizzled4* (*fz4)*; and the signal transducer Arm in larval and pupal wings. No expression pattern is observed for the Wnt ligands *Wnt5*, *Wnt7*, *Wnt9*, and *Wnt11*. (**B**) Ligands of canonical Wnt signaling bind to the receptor Fz9 preventing the proteasomal degradation of Arm and promoting its accumulation in the nucleus of cells in the foci during larval wing development and along the wing margin in larval and pupal wing development activating target genes. (**C**) In the pupal wing, Wnt1 is a likely morphogen binding to receptors Fz2 and Fz4 and leading to canonical Wnt signaling. Here, the transcription factor *Dll* is likely responding to this signal. *Dll* is responsible for the differentiation of black scales in the eyespot (*35*). The expression and function of *fz4* in the eyespot field indicate active PCP signaling in those cells that keep scales oriented correctly on the wing.

We hypothesize that Frizzled9 is the receptor involved in signal transduction along the wing margin and in the eyespot center cells resulting in the accumulation of cytoplasmic Arm (Fig. 9A). For Wnt ligands to reach the focal cells some distance away from the wing margin, they need to have a Wnt receptor expressed in those cells, and Frizzled9 is likely that receptor (Figs. 6 and 10B and fig. S13, I to P). This receptor is expressed not only along the wing margin but also in the intervein cells (Fig. 6, B and E, and fig. S13, I and J) where Arm is also present (Fig. 1, A and B). In human cell culture experiments, Frizzled9 was shown to be involved in the activation of canonical Wnt signaling and in the accumulation of β-catenin (Arm) in the cytoplasm (*51*). While functional experiments are still required, we propose that during larval wing development, canonical Wnt proteins are secreted from the wing margin and captured by Frizzled9 in the wing margin, in the finger projections, and in eyespot foci. This results in the accumulation of cytoplasmic Arm in those cells and eventually nuclear Arm (Figs. 1, E and F, and 10B). In *J. coenia* and *V. cardui*, knockout of *frizzled9* (named *frizzled3* by those authors) resulted in defects along the wing margin (*52*). *frizzled9* is also most likely involved in the transduction of Wnt signaling to activate *Dll* and *vg* at different thresholds along the wing margin (Figs. 3 and 10B), which are known target genes in *Drosophila* wings (*1*).

The accumulation of Arm in the eyespot centers, however, may proceed through an interplay between canonical and noncanonical Wnt signaling (Fig. 9A). *frizzled4*, a receptor typically associated with noncanonical Wnt signaling (*53*) is anti-colocalized with Arm in the eyespot foci, finger projections, wing margin, and veins (Figs. 6A, E, H, and K, and 10A and fig. S13, A to H), all the areas where Arm is present (Fig. 1, A and B). The anti-colocalization of *frizzled4* and Arm suggests that *frizzled4* functions via a noncanonical mode. None of the proposed noncanonical Wnt ligands (*Wnt5*, *Wnt7*, *Wnt9*, and *Wnt11*) are coexpressed with *frizzled4* in the stages sampled (Figs. 4 and 10A and fig. S12), indicating that noncanonical Wnt signaling is likely transduced through *frizzled4* independently of any Wnt ligand, similar to what has been proposed for *Drosophila* wings (*15*, *20*). Disruptions of *frizzled4* in *B. anynana* resulted in the appearance of two eyespots along the proximo-distal axis of a wing sector (Fig. 8, C to F, and fig. S12, E to K), not involving ectopic venation (fig. S15, H to K). These dual eyespots phenocopy a *Dll* overexpression phenotype previously studied and modeled via a reaction-diffusion mechanism using canonical Wnt signaling (*35*). We propose, thus, that *frizzled4*-mediated signaling represses canonical Wnt signaling, as also observed in arterial network organization in mice (*53*). This repression would restrict canonical Wnt signaling (Arm accumulation) to regions of the fingers and eyespot focus. Removal of *frizzled4* likely results in the up-regulation of *arm* and *frizzled9*, resulting in additional eyespot foci (Fig. 8, C to F).

We also propose that Arm accumulation in the eyespot centers during the larval stage leads to the activation of both *Wnt1* (Fig. 2, C and D) and *dpp* [bone morphogenetic protein (BMP) signaling] (*54*) in the pupal wing eyespot centers. Activation of both *Wnt1* and *dpp* signaling by Arm (β-catenin) has been shown in *Drosophila*, mice, and zebrafish in previous studies (*55*–*57*). Signaling by *Wnt1* (and *dpp*) might be involved in setting up the rings of color around the center during the pupal stage (*37*).

Other frizzled receptors were either present in distinct patterns or absent in the larval wings in the stages tested. We did not detect any expression of *frizzled* in the larval wing stages sampled, and *frizzled2* was expressed along a proximal wing domain. The presence of *frizzled2* in the proximal domain is likely due to the repression of *frizzled2* by Wnt signaling from the wing margin as previously shown in *Drosophila* larval wings (*58*).

### The spatial regulation of *frizzled2* likely involves repression from Wnt signaling at different domains of the pupal wings

During the first 18 to 24 hours of pupal wing development, we observed a very precise control of the domains over which the ligands *Wnt1*, *Wnt6*, and *Wnt10*; the receptors *frizzled2*, *frizzled9*, and *frizzled4*; and the signal transducer Arm are expressed (Figs. 1, 2, and 7), suggesting that they are regulated (and cross-regulated) by the signaling pathways where they function. Both *frizzled2* and *frizzled4*, along with Arm, are expressed in the eyespot focal cells, but *frizzled2* is anti-colocalized with *frizzled4* in the rest of the eyespot field (Fig. 7, E to G, fig. S14, D to F). Cross-regulation might also be happening between a Wnt signaling pathway using *Wnt1*, *Wnt6*, *Wnt10*, *frizzled9*, and Arm, expressed along the wing margin (Figs. 1, C and D; 2, C to F; and 7H), and *frizzled 2*, expressed at lower levels in the margin and chevron area (Fig. 7C and fig. 14, A and B). Similarly, *WntA* is anti-colocalized with *frizzled2* along the CSS (Fig. 3 and fig. S10). The anti-colocalization of *WntA* with *frizzled2* in the CSS is also consistent with expression and functional data in *V. cardui* where *WntA* and *frizzled2* have anti-colocalized expression domains, and loss of *WntA* results in expansion of *frizzled2* in the domains of *WntA* expressing cells (*52*).

We propose a model (Fig. 9B) for the spatial regulation of *frizzled2* in *B. anynana* which involves down-regulation of this gene by Wnt signaling from the margin, from the eyespot centers, and along the CSS. *frizzled2* might be initially homogeneously expressed in the pupal wing, but Wnt signaling from the wing margin (Wnt1, Frizzled9, and Arm), the eyespot center (Wnt1, Frizzled4, and Arm), and along the CSS (WntA) represses the activity of *frizzled2* in their respective domains resulting in the precise domains of *frizzled2* expression we observe in the pupal wings (Fig. 9B). This model is consistent with Wnt1-dependent signaling in *Drosophila* repressing *frizzled2* in the dorsal-ventral axis of the wing disc and in the segments of the embryo (*58*, *59*).

### *frizzled4* is involved in regulating scale polarity in the eyespot field

*frizzled4* is also involved in the orientation of the scale cells in the eyespot field of *B. anynana*. The gene is expressed in the eyespot field during the pupal stage (Figs. 7, A and B, and 10A), and disruptions of *frizzled4* resulted in scale orientation defects (Figs. 8, H to J, and 9C). The PCP pathway, however, is most probably independent of any Wnt ligand as previously shown in *Drosophila* wings (*15*, *20*). This pathway, when disrupted, results in changes in the orientation of cellular protrusions such as trichomes in *Drosophila* wings (*15*) and mouse skin (*16*). Scales appear to behave in the same way as trichomes in *Drosophila* wing, despite not being homologous traits. Butterfly wing scales are F-actin–based protrusions from the epithelial cells which are deposited with chitin and are homologous to bristles (*60*, *61*).

### Wnt1 is likely functioning with Frizzled2, Frizzled4, and Arm in pupal wings to determine eyespot size

Wnt1 was previously proposed as a morphogen produced in the eyespot centers (*32*) to regulate eyespot size in *B. anynana* (*37*). The mechanism by which Wnt1 operates in the determination of the eyespot rings, however, has not yet been explored. Here, we confirmed the presence of *Wnt1* mRNA in the eyespot central cells during early pupal wing development coexpressed with *frizzled2*, *frizzled4*, and Arm (Fig. 10A) (*37*). The Wnt1 ligand is likely being secreted by the central cells and captured by the receptors Frizzled4 and Frizzled2 (to a lesser extent), whose transcripts are expressed across the whole eyespot field (Figs. 7, A to D, and 10C; and fig. S14, A to C). Active canonical Wnt signaling via the Frizzled receptors should result in higher levels of Arm in the eyespot rings. We, however, only observed strong Arm accumulation in the center of the eyespot during pupal wing development (Fig. 1, C and D). Elevated cytoplasmic Arm levels in the eyespot central cells likely happen due to the coexpression of *frizzled2*, *frizzled4*, and *frizzled* (Fig. 7, A to D, and figs. S13, U to X, and S14) during pupal wing development. In the rest of the eyespot field, Wnt signaling is probably being transduced via canonical signaling with lower levels of Arm which are difficult to detect via immunostaining.

### *WntA* appears to function as a morphogen and as a scale color regulator at different stages of wing development

We observed two distinct phenotypes in *B. anynana WntA* crispants that suggest that this gene may have two distinct functions. The most obvious phenotype was the loss of brown scales in the CSS and BSS bands and the appearance of orange scales in their place. The appearance of orange scales is likely due to the ectopic expression of *Optix* in those regions, a gene that might normally be repressed by WntA in the pupal stage (Fig. 5, K to M). Such ectopic expression of *Optix* was observed in *H. erato* pupal wings in *WntA* crispants (*42*). The less obvious phenotypes were the narrower and distorted bands of the CSS and the loss of the light chevron pattern marginal elements (Fig. 5K). These two effects suggest that *WntA* might also function as a morphogen to define the width of the CSS and the MBS. This function may take place during the larval stage, as *WntA* is expressed in the wing margin only at this stage and is expressed in a narrower domain in the center of the future CSS band (Fig. 5, B and C). *WntA* likely operates noncanonically, independently of Arm since no Arm expression is observed in the domains over which *WntA* has strong expression, except for the wing margin (Figs. 1 and 3, B to I). This noncanonical signaling of *WntA* is consistent with a similar hypothesis in other butterflies (*52*). *WntA* has also been shown to be involved in the patterning of the BSS, the CSS, and the MBS in *J. coenia*, *Pararge aegeria*, *V. cardui*, *A. vanilla*, and *Heliconius* butterflies (*34*, *38*, *41*) and in the eyespots of *V. cardui* (*41*, *52*). More recently, *WntA* has also been proposed as a morphogen that induces cell differentiation in a concentration-dependent manner at some distance away from producing cells (*52*).

We note that the discal spots are not affected in *WntA* knockout adults (Fig. 5, K to M). Other *Wnts* (*Wnt1*, *Wnt6*, and *Wnt10*) are expressed in this domain and are likely important for the identity of the cells there (Fig. 2, A and B, and fig. S8). Future studies with functional knockouts of these ligands will unravel their role in the discal spot. The expression of *Wnt1*, *Wnt6*, and *Wnt10* along in this spot is conserved across butterflies and likely plays a similar role in discal spot development (*34*).

In summary, we illustrated the expression and function of different Wnt signaling pathway members in larval and pupal wings of *B. anynana* wings that contribute to the precise differentiation of eyespots, bands, and chevron patterns. Further studies are necessary to examine the function of several of these genes, especially that of *frizzled9*, and the interaction between *frizzled4*, *frizzled9*, and *frizzled2*. The interaction of Wnt signaling with BMP also needs further study, as this has been proposed to function in the reaction diffusion mechanism for eyespot center formation during the larval stage (*35*) and ring differentiation during the pupal stage (*54*). The Wnt signaling mechanism for eyespots, however, is likely conserved in other nymphalid butterfly species, as observed with the expression pattern of Arm (fig. S6) and canonical Wnt ligands (*34*). Studying complex signaling pathways, such as Wnt signaling, in simple two-dimensional (2D) surfaces such as butterfly wings may help unravel the function of this pathway in more complex 3D traits such as legs (*62*, *63*), antennae (*45*), and horns (*64*, *65*), where Wnt signaling is also fundamental to trait development and toward our understanding of this signaling in higher animals.

## MATERIALS AND METHODS

### Sequence alignment and phylogenetic tree construction

Nucleotide sequences were obtained from NCBI and FlyBase. Alignments were carried out using ClustalW (*66*) with the default parameters in “SLOW/ACCURATE” alignment option in GenomeNet.

Using RAxML (Randomly Accelerated Maximum Likelihood) v8.1.20 a maximum likelihood tree was created with model PROTGAMMAJTT and default parameters with 100 bootstraps (*67*) using ETE v3.1.1 (*68*) implemented on GenomeNet. Similar trees were obtained with FastTree with slow Nearest-Neighbor Interchanges (NNIs) and MLACC = 3 (to make the maximum-likelihood NNIs more exhaustive) (*69*) ETE v3.1.1 (*68*).

### Rearing *B. anynana*

*B. anynana* larvae and adults were fed corn leaves and mashed bananas, respectively. The individuals were reared at 27°C with a 12-hour day/12-hour night cycle under 60% humidity. Male and female *B. anynana* butterflies are not sexually dimorphic with respect to the wing patterns studied in the present work.

No animal experimentation permits were required for the experiments conducted here. *B. anynana* butterflies have been reared in the lab since 1988 and imported under an Agri-Food & Veterinary Authority permit to the lab in Singapore.

### CRISPR-Cas9

*arm*, *WntA*, *Wnt7*, and *fz4* embryonic CRISPRs were carried out on the basis of the protocol described in (*45*, *70*) (table S2). One to two RNA guides were designed using CRISPRdirect or CHOPCHOP to target the coding sequence of these genes (see the Supplementary Materials for sequences). Cas9 protein (Integrated DNA Technologies, catalog no. 1081058) (300 ng/μl) along with the guide RNA at a concentration of 300 ng/μl were mixed in molecular grade water with Cas9 buffer (New England Biolabs, catalog no. M0386S). Embryos were injected 6 hours after egg laying for *arm*, 1 to 3 hours for *WntA*, and 3 hours for *frizzled4* and *Wnt7*. Embryos were kept in petri dishes inside a temperature-controlled incubator with moistened cotton to maintain humidity. The larvae were reared in mesh cages and fed young corn leaves. After pupation, the larvae were transferred to plastic containers, and after eclosion, the adults were frozen at −20°C and imaged under a Leica DMS1000 microscope. Scales were bleached using sodium hypochlorite (Clorox).

For next-gen sequencing, DNA was extracted from affected wings using an Omega tissue DNA extraction kit (catalog no. D3396-01). For the *arm* and *WntA* crispants, primers flanking around the CRISPR target site were used to amplify the region of interest (primer sequences in table S1). For *arm*, adapters and indices were added to the polymerase chain reaction (PCR) product via a two-step PCR reaction (table S1), followed by purification of the PCR products. The samples were sequenced using an Illumina iSeq 100 sequencer. For *WntA*, the amplicon was sent to Azenta Inc. for Illumina sequencing. Reads were aligned to the reference wild-type (WT) *arm* and *WntA* sequences using Geneious R10 (*71*).

For Sanger sequencing (*fz4*) of the crispants and WT, DNA was extracted as described above using an Omega tissue DNA extraction kit. PCR was amplified using the gene-specific primers (primer sequences in table S1) and purified. Sequencing was carried out at 1st BASE, Singapore. CRISPR indel was analyzed using Synthego Performance Analysis, ICE Analysis. 2019. v3.0. (last accessed October 2022) (https://synthego.com/products/bioinformatics/crispr-analysis).

### Immunostainings

The moment of pupation was timed using an Olympus tough TG-6 camera, and pupal wings were dissected and stained on the basis of a protocol described in (*36*). Briefly, wings were dissected in 1× phosphate-buffered saline (PBS) under ice for larval wings and in 1× PBS at room temperature for pupal wings. Afterward, wings were transferred to fix buffer (table S3) with 4% formaldehyde in ice for larval wings and at room temperature for pupal wings. After fixation, the wings were transferred to ice, washed with 1× PBS, and kept in block buffer (table S3) overnight. Primary antibodies were diluted in wash buffer at the concentration of 1:1000 anti-Arm [rat; (*36*)] and 1:20000 anti-Spalt [guinea pig GP66.1; (*72*)] and stained overnight at 4°C. Afterward, the wings were washed four times in wash buffer (table S3), for 15 min each time. Wings were then incubated in 1:500 secondary antibody at the concentration 1:500 with anti-rat AF488 (Invitrogen, #A-11006) and anti-guinea pig AF555 (Invitrogen, #A-21435), followed by four washes in wash buffer (table S3). Last, the wings were mounted in an in-house mounting media (see table S3) and imaged under an Olympus fv3000 microscope.

### Chromogenic based in situ hybridization

In situ hybridization experiments were carried out on the basis of the protocol described in (*36*). Pupal wings were dissected in 1× PBS and fixed in 4% formaldehyde in 1× Phosphate Buffer Saline with Tween20 (PBST) at room temperature. After fixation, wings were treated with proteinase k for 5 min and afterward with glycine (100 mg/ml) in 1× PBST. Wings were then washed with 1× PBST and transferred to prehybridization buffer (table S4) at 65°C followed by incubation in hybridization buffer (table S4) with probes against *Wnt1* (see the Supplementary Materials for sequences) at 65°C for 16 to 24 hours. After hybridization, wings were washed with prehybridization buffer at 65°C, five times, for 30 min each time. Afterward, wings were brought down to room temperature and washed four times with 1× PBST. Wings were then incubated in block buffer (table S4) for 60 min, followed by incubation in anti-digoxygenin (Sigma-Aldrich, catalog no. 11093274910) at the concentration of 1:2000 in block buffer. The wings were then washed five times with a block buffer, 10 min each time. The wings were then washed two times in alkaline-phosphatase buffer, for 5 min each time. After washing, wings were transferred to alkaline-phosphatase buffer supplemented with bromochloroindolyl phosphate–nitro blue tetrazolium (Promega, catalog no. S3771) and left for 4 to 6 hours in the dark for color to develop. Once the color developed, wings were washed two times with 1× PBST and mounted in 60% glycerol, and imaged under a Leica DMS1000 microscope.

### Fluorescent based in situ hybridization (HCR3.0)

Fluorescent in situ hybridization was performed on the basis of the protocol developed by Choi *et al.* (*48*) with a few modifications optimized for butterfly wing tissue. Briefly, wings were dissected in 1× PBS and fixed in 4% formaldehyde in 1× PBST. After fixation, wings were washed with 1× PBST and permeabilized using a detergent solution (*73*). The wings were again washed with 1× PBST and with 5× Saline-Sodium Citrate with Tween20 (SSCT) followed by the addition of 30% probe hybridization buffer. Afterward, wings were incubated overnight at 37°C in a chamber with 30% probe hybridization buffer and HCR3.0 probes. The next day, wings were washed five times with 30% probe wash buffer at 37°C followed by two washes with 5× SSCT at room temperature. Wings were then incubated in an amplification buffer with secondary fluorescent hairpin probes (Molecular Instruments) in the dark for 16 to 20 hours. The next day, wings were washed four times in 5× SSCT, mounted in an in-house mounting media, and imaged under an Olympus fv3000 microscope.
