# Supplementary material for: Spatial and temporal regulation of Wnt signaling pathway members in the development of butterfly wing patterns
Source: Sci Adv. 2023 Jul 26;9(30):eadg3877. doi: 10.1126/sciadv.adg3877 (PMC10371022; doi:10.1126/sciadv.adg3877)
Supplement: Supplementary file 1 — Table S1 to S4 Figs. S1 to S15 [file sciadv.adg3877_sm.pdf]

Science Advances
AAAS

Supplementary Materials for

Spatial and temporal regulation of Wnt signaling pathway members in the development of butterfly wing patterns

Tirtha Das Banerjee *et al.*

Corresponding author: Tirtha Das Banerjee, tirtha_banerjee@u.nus.edu;
Antónia Monteiro, antonia.monterio@nus.edu.sg

Sci. Adv. **9**, eadg3877 (2023)
DOI: 10.1126/sciadv.adg3877

This PDF file includes:

Tables S1 to S4
Figs. S1 to S15

Supplementary information

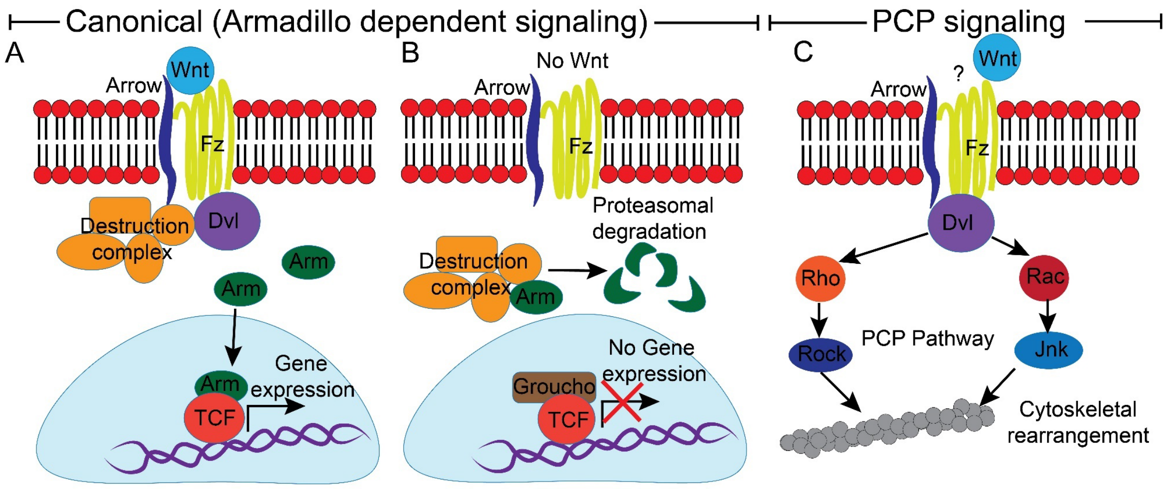

Figure S1: Canonical (Armadillo dependent) and PCP (non-canonical) Wnt signaling. (A and B) In the canonical Wnt signaling in the presence of Wnt the receptor Frizzled (Fz) and co-receptor Arrow with the help of Dishevelled (Dvl) prevents the destruction complex from destroying cytoplasmic Arm. Arm moves into the nucleus and with the help of co-transcriptional activator TCF and activates gene expression. In the absence of Wnt, the destruction complex helps in the proteasomal degradation of Arm, and due to the absence of Arm in the nucleus the Wnt repressor Groucho binds TCF and prevents gene expression. (**C**) In the non-canonical planar cell polarity pathway Dvl activates the G-protein complex Rho and Rac which activates the Rock and Jnk signaling respectively. This signaling pathway is responsible for the cytoskeletal rearrangement of the cell.

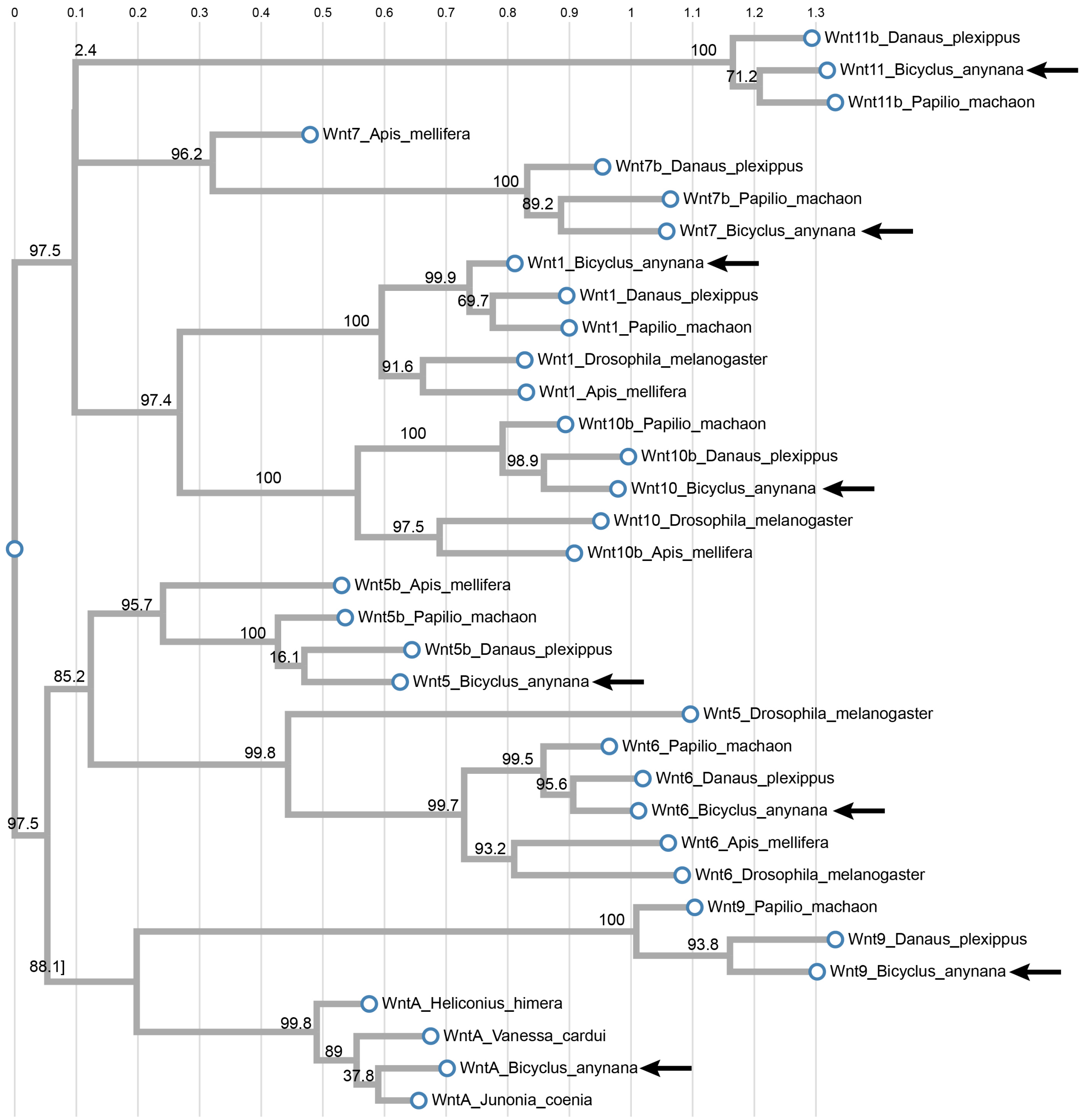

Figure S2. Phylogenetic tree of *Wnt* genes created using fasttree (Maximum likelihood). Vertical lines indicate mean number of nucleotide substitutions per site. Numbers above branches represent bootstrap branch support. Arrows point to the eight *Wnt* genes in the genome of *B. anynana*.

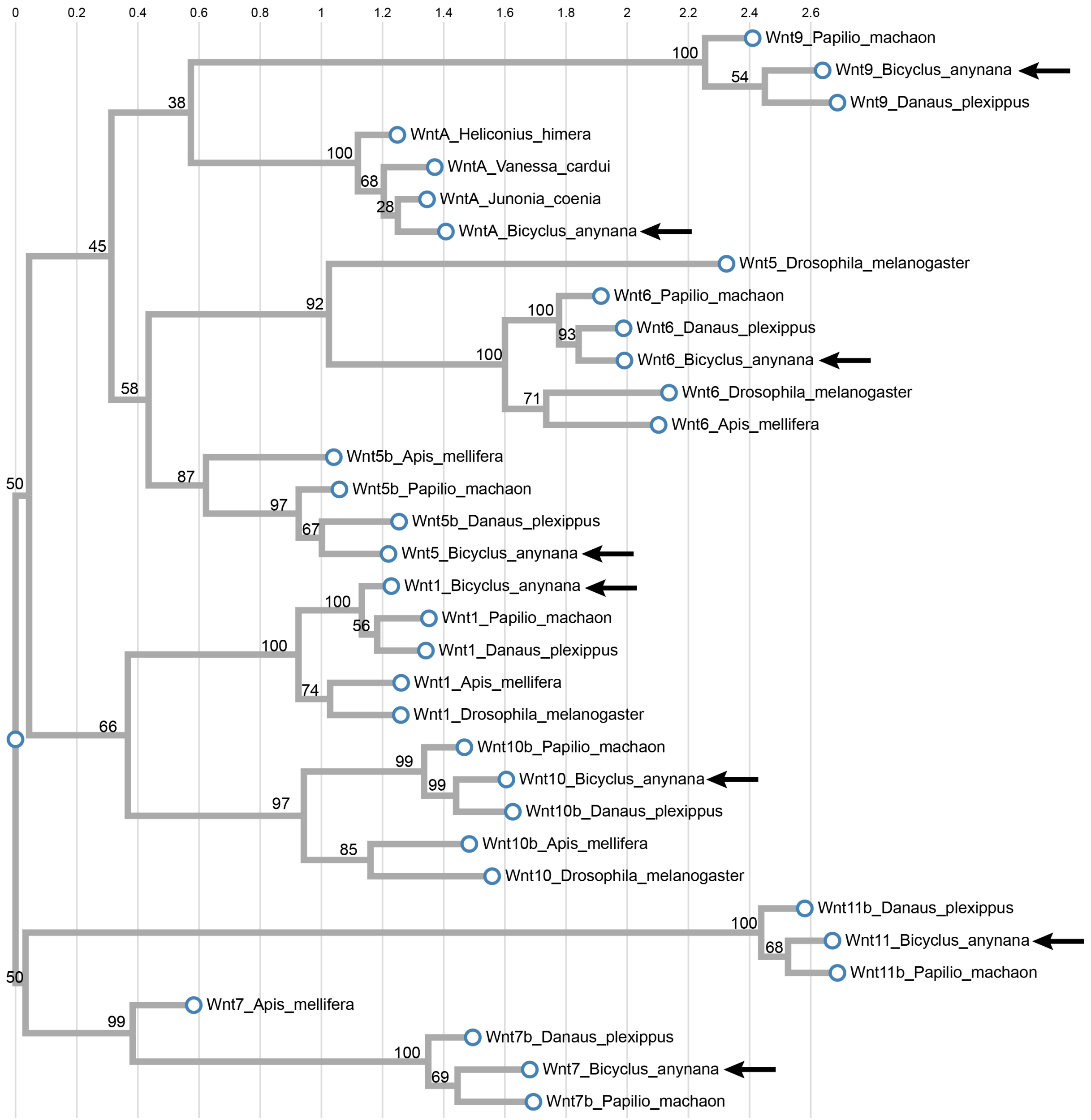

Figure S3. Phylogenetic tree of *Wnt* genes created using RAxML. Vertical lines indicate mean number of nucleotide substitutions per site. Numbers above branches represent bootstrap branch support. Arrows point to the eight *Wnt* genes in the genome of *B. anynana*.

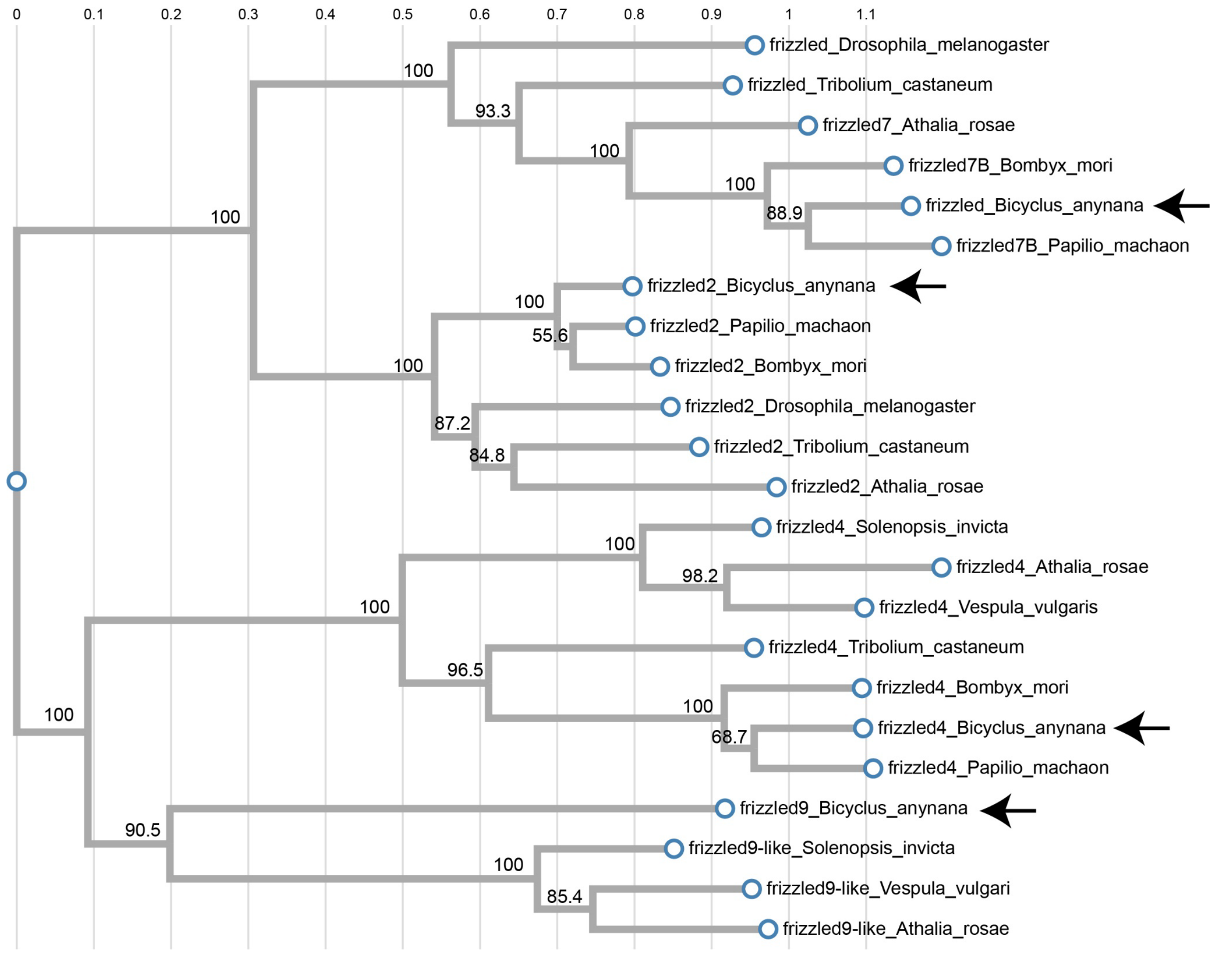

Figure S4. Phylogenetic tree of *frizzled* genes created using fasttree (Maximum likelihood). Vertical lines indicate mean number of nucleotide substitutions per site. Numbers above branches represent bootstrap branch support. Arrows point to the four *frizzled* genes in the genome of *B. anynana*.

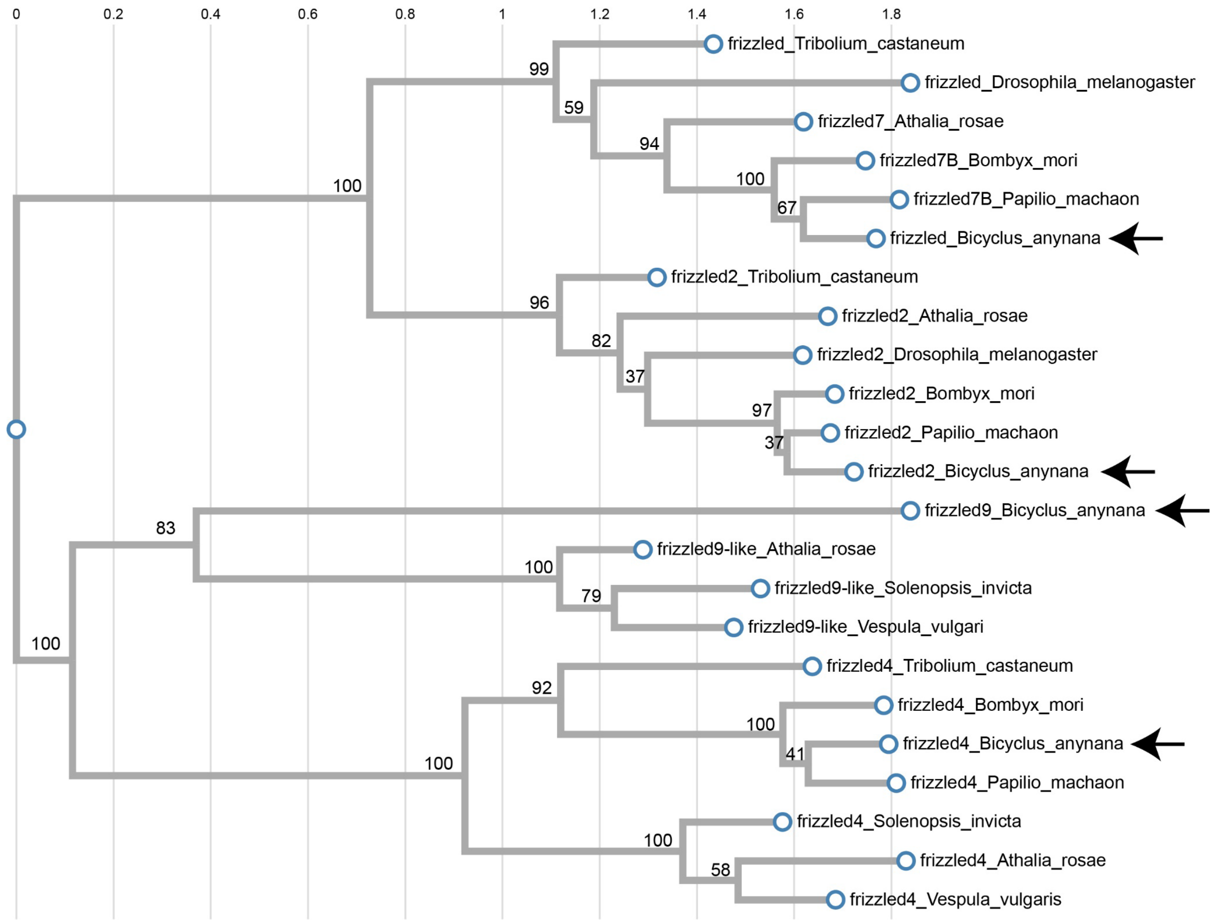

Figure S5. Phylogenetic tree of *frizzled* genes created using RAxML. Vertical lines indicate mean number of nucleotide substitutions per site. Numbers above branches represent bootstrap branch support. Arrows point to the four frizzled genes in the genome of *B. anynana*.

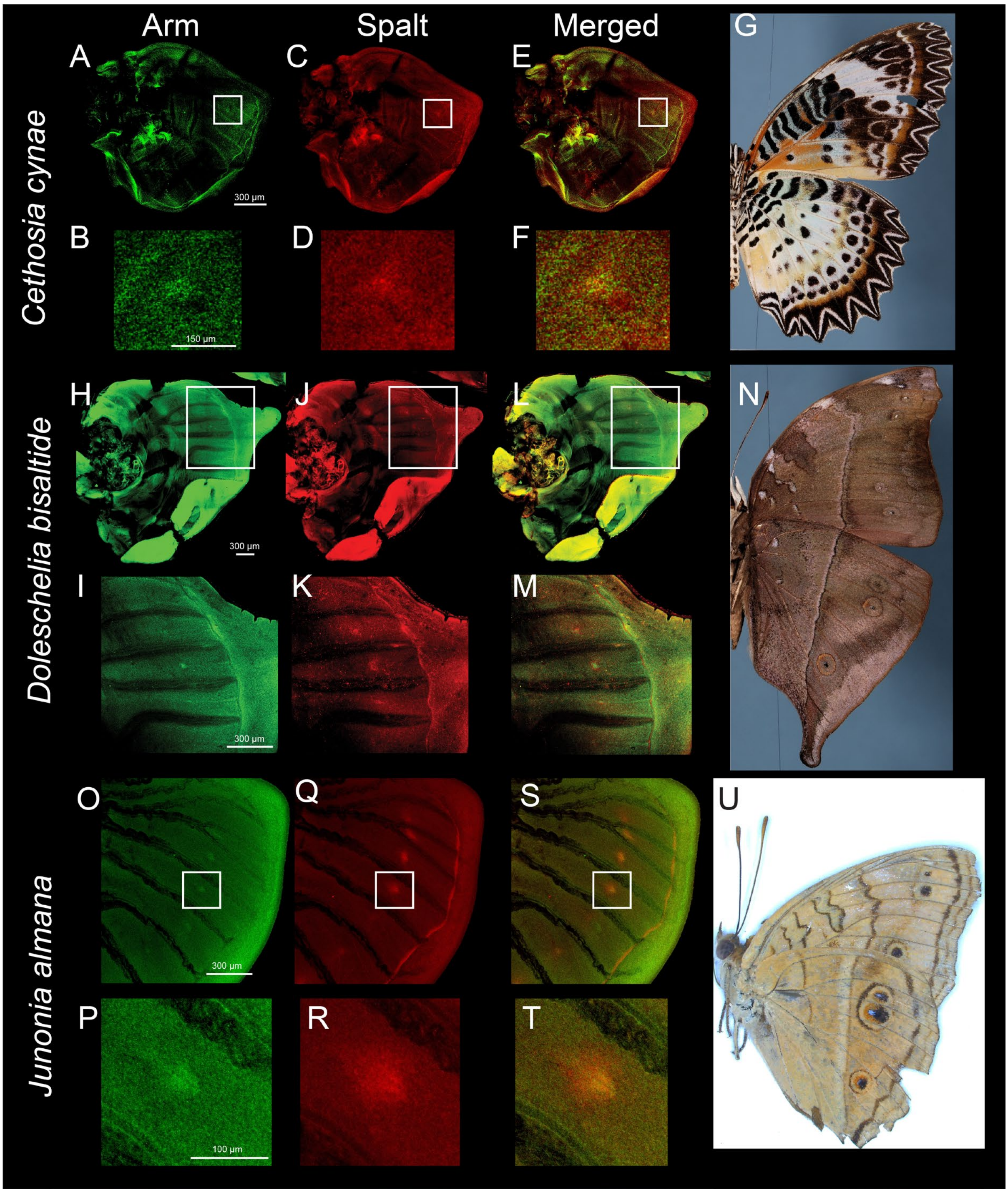

Figure S6. Expression of Arm and eyespot marker gene Spalt in *Cethosia cynae*, *Doleschelia bisaltide* and *Junonia almana* larval wings. Both (**A, B**) Arm and (**C, D**) Spalt are expressed in the foci of *Cethosia cynae* larval wing. (**E and F**) Merged expression of Arm and Spalt. (**G**) WT *Cethosia cynae* adult. (**H, I**) Arm and (**J, K**) Spalt are expressed in the foci of *Cethosia cynae* larval wings. (**L, M**) Merged expression of Arm and Spalt. (**N**) WT *Doleschelia bisaltide* adult. (**O, P**) Arm and (**Q, R**) Spalt are expressed in the foci of *Junonia almana* larval wings. (**S, T**) Merged expression of Arm and Spalt. (**U**) WT *Junonia almana* adult.

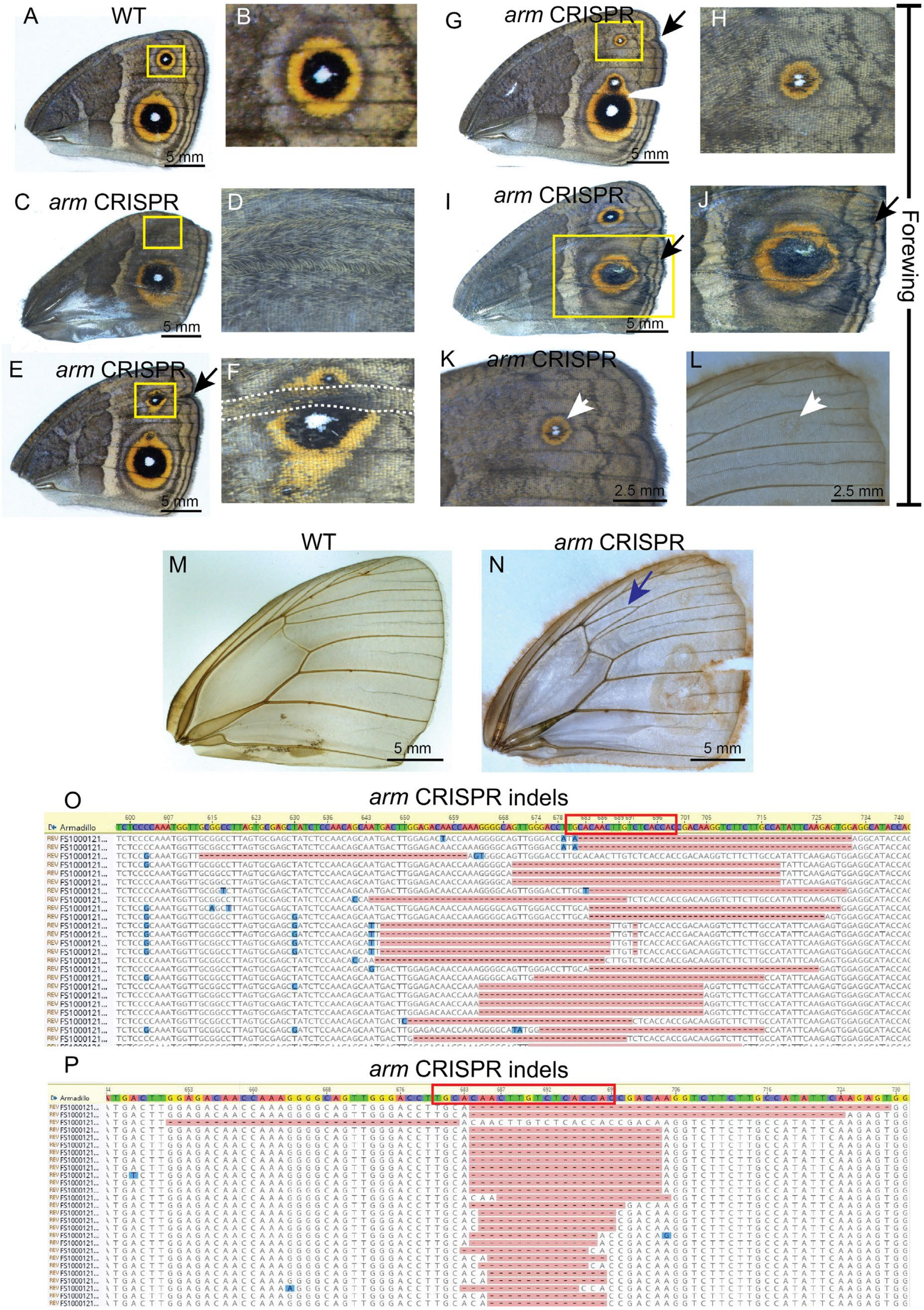

Figure S7. Function of *arm* in the eyespot development of *B. anynana*. (**A, B**) WT adult forewing. *arm* disruptions, using CRISPR-Cas9, resulted in (**C, D**) the complete loss of an eyespot and (**E-H**) in two foci differentiating in a single wing sector. (**I, J**) *arm* CRISPR showing defects in the wing margin (black arrows). (**K, L**) Descaled *arm* CRISPR wing showing that the differentiation of two eyespot

centers is not due to ectopic venation (white arrow). (**M**) WT wing venation. (**N**) *arm* CRISPR adult wing showing venation defect (blue arrow). (**O, P**) Next-generation sequencing (NGS) of the affected wing region of the *arm* CRISPR individual in panels **C** and **D** showing deletions at the target site (red box). Note the folds in the wing margin present in both WT and CRISPR wings not to be confused with veins (hollow cuticular tube).

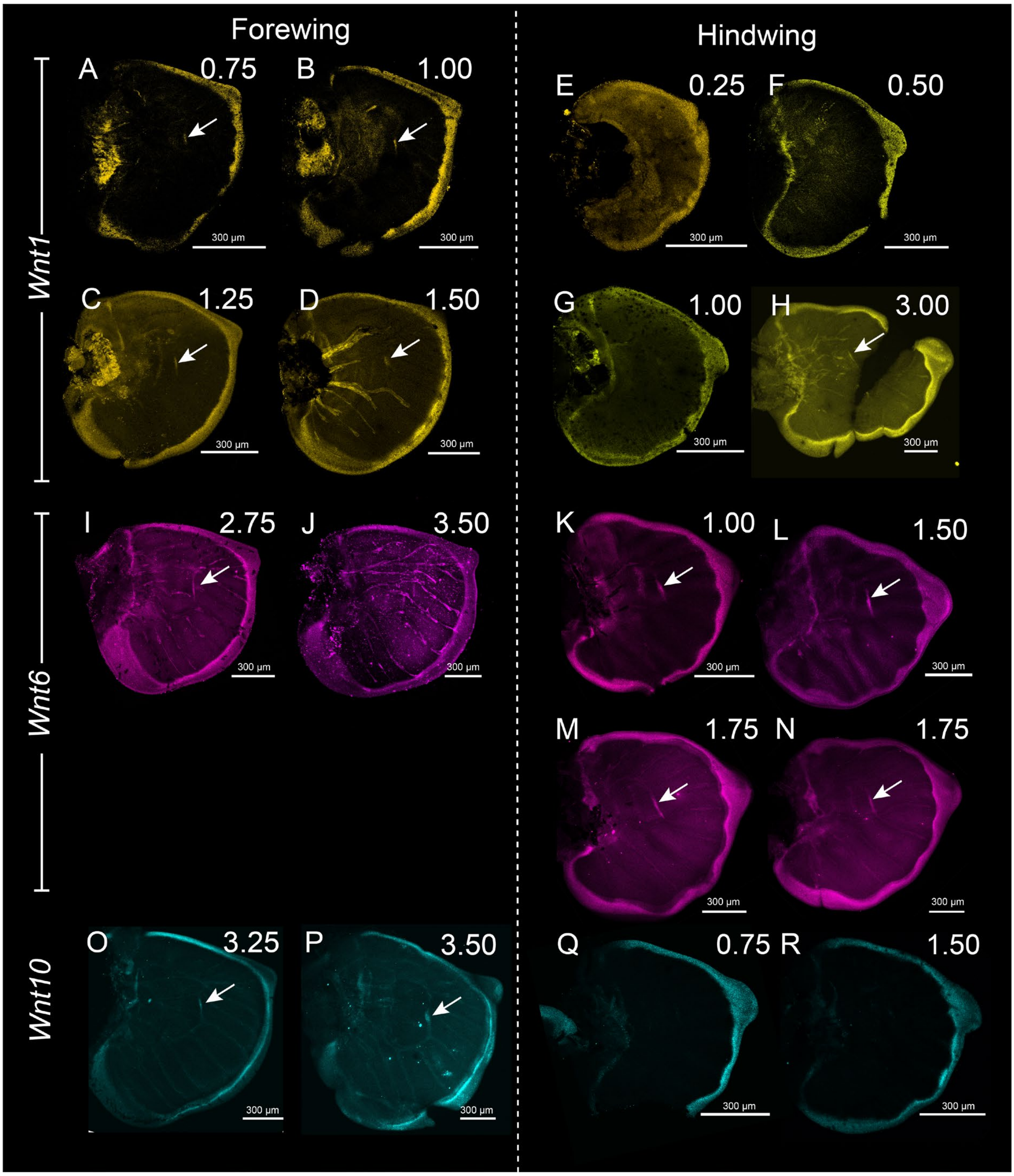

Figure S8. Expression of *Wnt1*, *Wnt6*, and *Wnt10* in larval wings. Expression of (**A-H**) *Wnt1*, (**I-N**) *Wnt6*, and (**O-R**) *Wnt10* in the developing larval wings. Expression is observed along the wing margin and in the discal spot (white arrows).

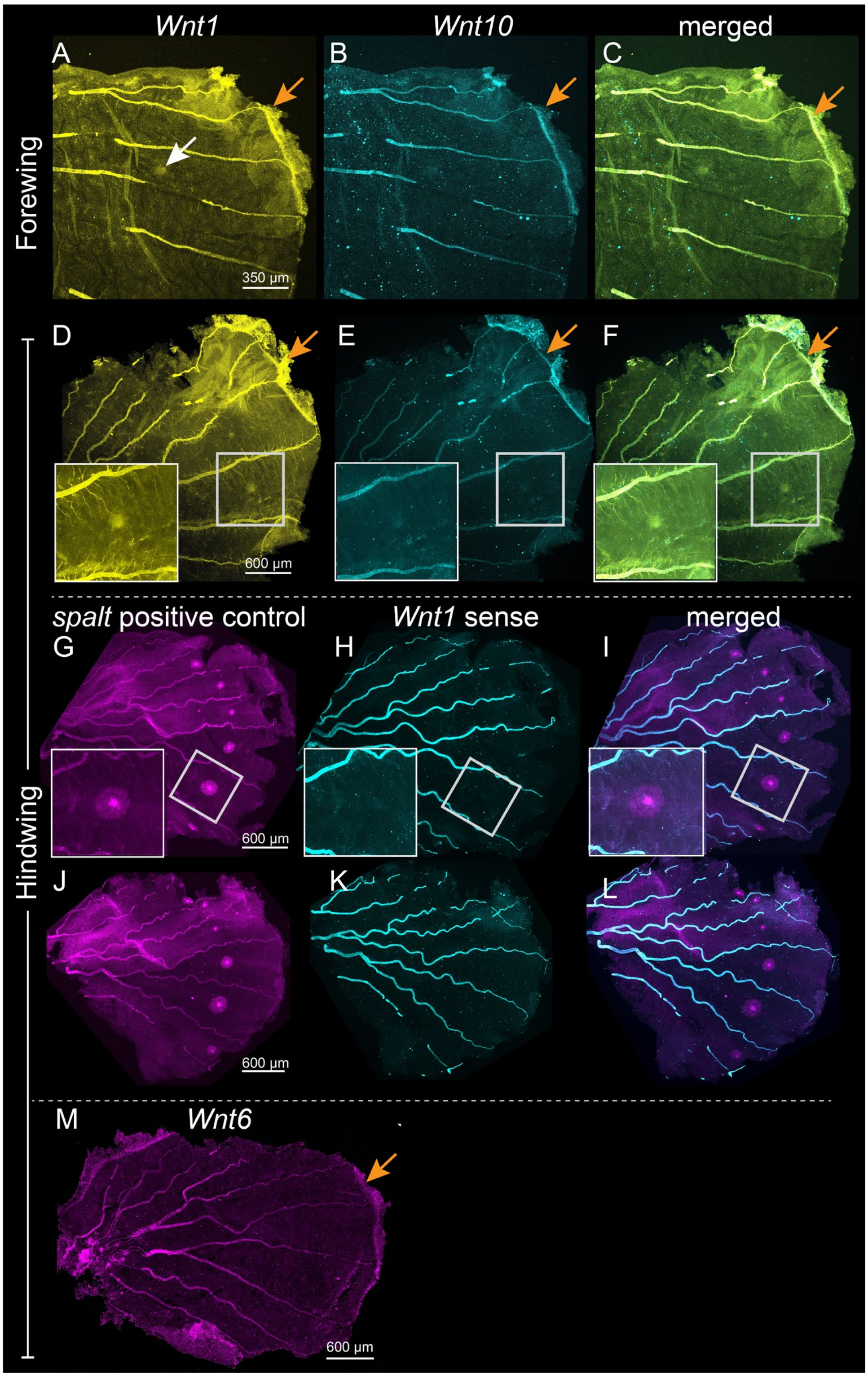

Figure S9. Expression of *Wnt1, Wnt6, Wnt10,* and *Wnt1* sense in 18-24hrs pupal wing. (**A-F**) In an 18-24 hrs pupal wing *Wnt1* and *Wnt10* are expressed in the wing margin (orange arrow), additionally *Wnt1* is expressed in the center of the eyespots (white arrow). (**G-L**) Expression of *Wnt1* sense and spalt in 18-24 hrs pupal wing showing no expression for *Wnt1* sense, while *spalt* (used as positive control) shows expression in the eyespot center and in the black scale cells. (**M**) Expression of *Wnt6* in the wing margin (orange arrow).

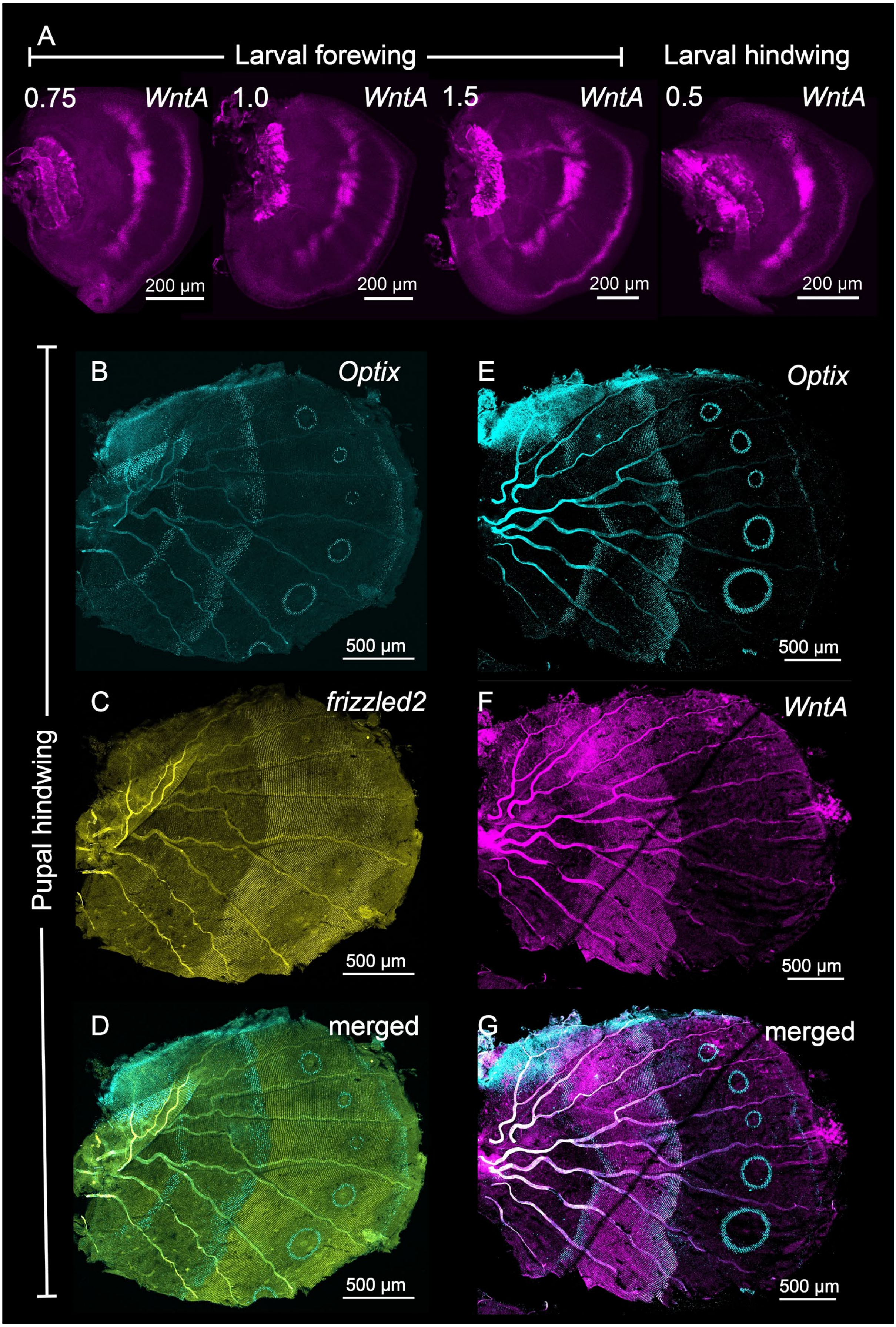

A
Larval forewing
Larval hindwing
0.75
WntA
1.0
WntA
1.5
WntA
0.5
WntA
200 µm
200 µm
200 µm
200 µm
Pupal hindwing
B
Optix
500 µm
C
frizzled2
500 µm
D
merged
500 µm
E
Optix
500 µm
F
WntA
500 µm
G
merged
500 µm

Figure S10. Expression of *WntA* in larval and 18-24 hrs pupal wings; and *frizzled2* and *Optix* in the 18-24 hrs pupal wings of *B. anynana*. (**A**) Expression of *WntA* in the larval wing development along the central symmetry system and marginal band system. (**B-D**) Co-expression of *Optix* and *frizzled2* in an 18-24 hrs pupal wing. (**E-G**) Co-expression of *Optix* and *WntA* in an 18-24 hrs pupal wing. Note the precise cell boundary of *frizzled2* and *Optix* (as well as *WntA*).

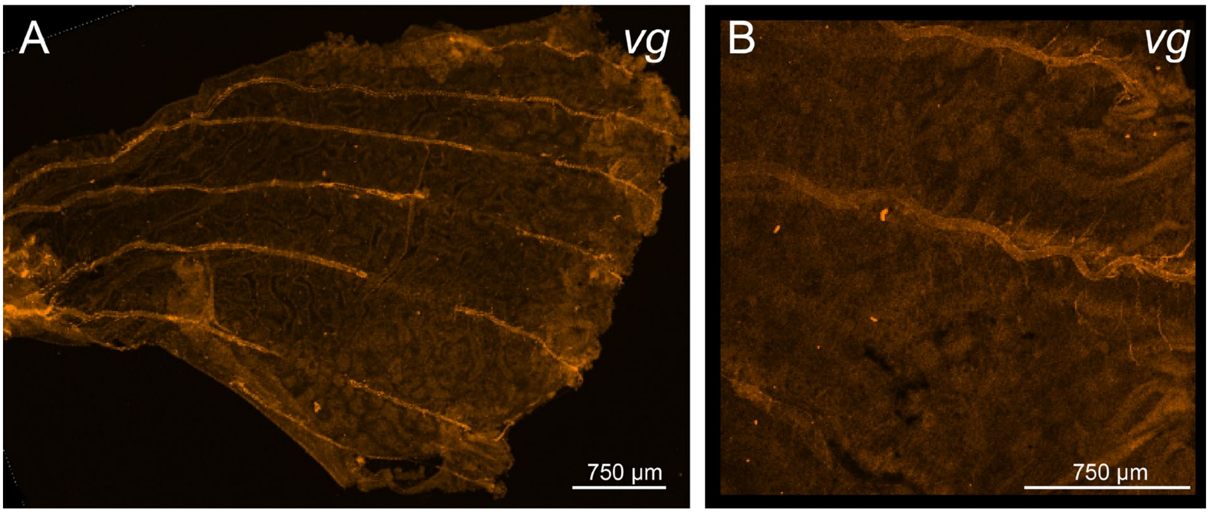

Figure S11. Expression of *vg* in in the pupal wing. No specific domain of expression was observed for *vg* in 18-24 hrs pupal wings.

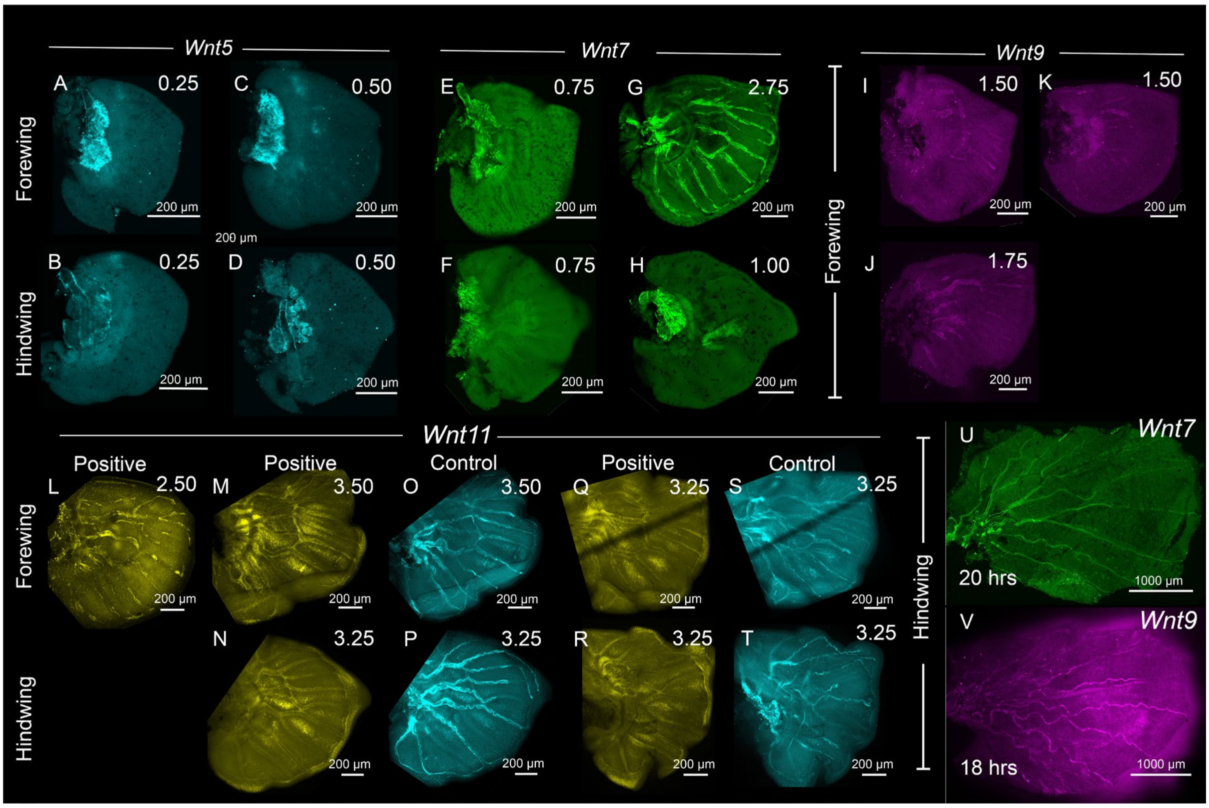

Figure S12. No specific expression of *Wnt5, Wnt7* and *Wnt9* in the stages tested during larval wing development of *B. anynana*. Expression of (**A-D**) *Wnt5*, (**E-H**) *Wnt7*, and (**I-K**) *Wnt9* in the larval wings showing no specific expression domains. (**L-T**) Expression of *Wnt11* and control (only secondary probes). *Wnt11* seems to have slightly elevated levels along the wing margin and expression in the

intervein cells but similar expression pattern in the intervein cells is also obtained in the control wings. Expression of (**U**) *Wnt7* and (**V**) *Wnt9* in 18-24 hrs pupal wings.

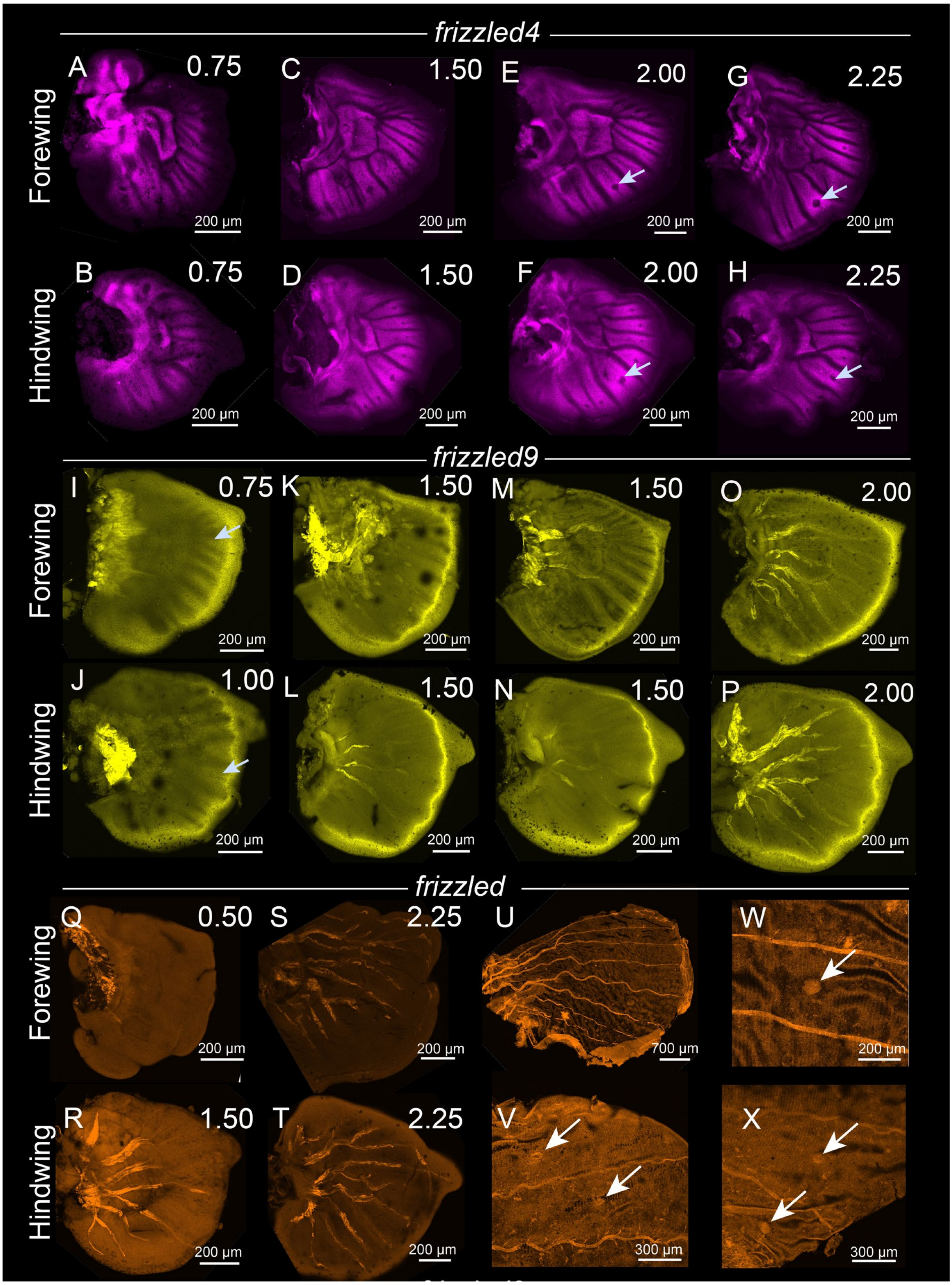

Figure S13. Expression of *frizzled4*, *frizzled9* and *frizzled* in the larval wings and *frizzled* in the pupal wings of *B. anynana*. (A-H) Expression of *frizzled4* in the developing larval wings. During the

early larval stage *frizzled4* is expressed in the intervein cells. As the wing develops, *frizzled4* continues to express in the intervein cells but is down-regulated in the eyespot centers. **(I-P)** Expression of *frizzled9* in larval wings is present initially along the wing margin and in broad fingers projecting from the margin (arrows in **I** and **J**), which become less visible as the wing ages. (**Q-X**) Expression of *frizzled* in the larval stages tested showed no expression, while in the pupal stage faint expression is observed in the center of the eyespots.

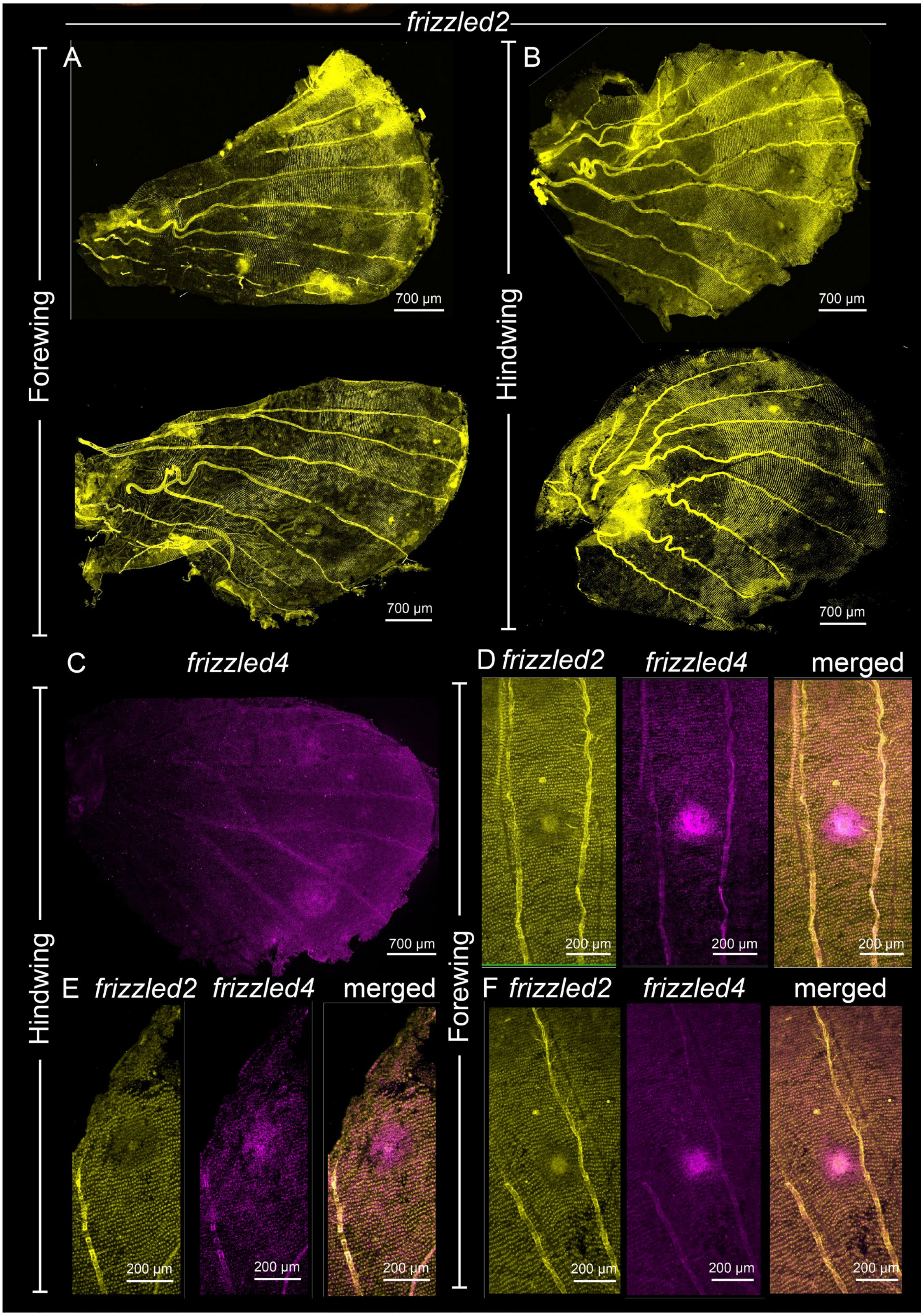

Figure S14. Expression of *frizzled2,* and *frizzled4* in the larval and pupal wings of *Bicyclus anynana*. (**A, B**) Expression of *frizzled2* in 18-24 hrs pupal wings where *frizzled2* has lower expression in the CSS, in the eyespot field, and in the wing margin. (**C**) Expression of *frizzled4* in a 18-24 hrs pupal

hindwing showing stronger expression in the eyespot center and in the eyespot field. (**D-F**) Anti-colocalized expression of *frizzled2* and *frizzled4* in the eyespot field.

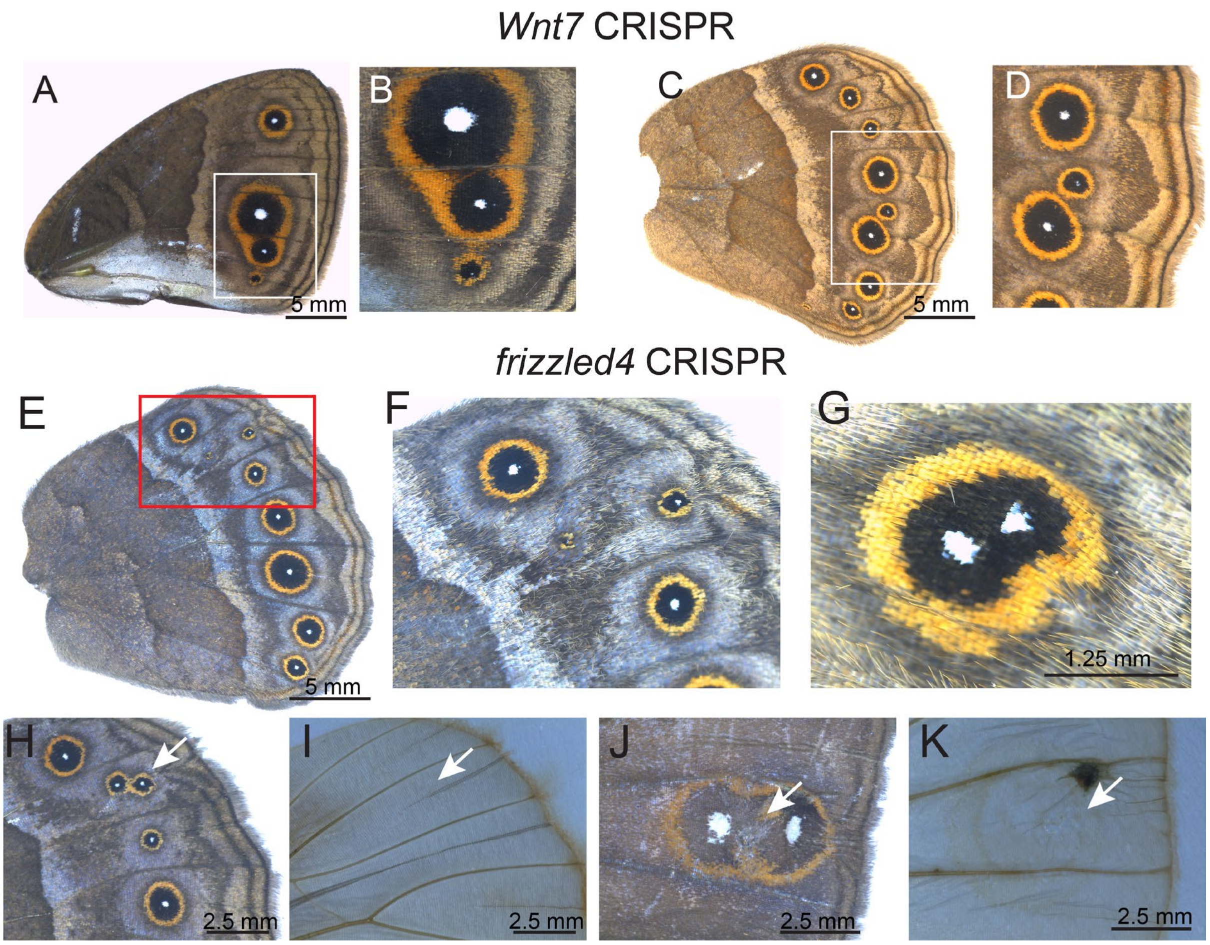

Figure S15. CRISPR-Cas9 on *Wnt7* and *frizzled4* in *Bicyclus anynana*. (A-D) Knock-out of *Wnt7* resulted in venation defects in both forewings and hindwings. These defects resulted in additional eyespots forming in each wing sector. (**E-G**) *frizzled4* knockout resulted in two eyespot centers differentiating in the same wing sector. (**H-K**) *frizzled4* knockout wings with scales removed showing that the differentiation of two eyespot foci are not due to ectopic venation (white arrows). Note the fold in the wing margin not to be confused with a vein (hollow cuticular tube).

Table S1: Primer table

Sl. No.	Primer name	Sequence
1.	Wnt1_insitu_F	CAGCAGCTGGATTTTGTCAG
2.	Wnt1_insitu_R	TATTGTGCCGTTGTCATCGT
3.	arm_CRISPR_guide_F	GAAATTAATACGACTCACTATAGG**GTGGTGAGACAAGTTGTGCA**GTTTTAGAGCTAGAAATAGC
4.	fz4_CRISPR_guide1_F	GAAATTAATACGACTCACTATAGG**GGACTGTTCTCTATTCCCGG**GTTTTAGAGCTAGAAATAGC
5.	fz4_CRISPR_guide2_F	GAAATTAATACGACTCACTATAGG**GATACCGTTGGACACGACGA**GTTTTAGAGCTAGAAATAGC
6.	Wnt7_CRISPR_guide1_F	GAAATTAATACGACTCACTATAGG**GCGACAAGTACCACCGAGCG**GTTTTAGAGCTAGAAATAGC
7	Wnt7_CRISPR_guide2_F	GAAATTAATACGACTCACTATAGG**AGACGCATCTCGTCATACGC**GTTTTAGAGCTAGAAATAGC
8.	WntA_CRISPR_guide_F	GAAATTAATACGACTCACTATAGG**CGCTCTGTCAGCAGCAGCAT**GTTTTAGAGCTAGAAATAGC
9.	WntA_CRISPR_guide_R	GAAATTAATACGACTCACTATAGG**ACGCCGCGGCATTGGCAGTG**GTTTTAGAGCTAGAAATAGC
10.	CRISPR_guide_R	AAAAGCACCGACTCGGTGCCACTTTTTCAAGTTGATAACGGACTAGCCTTATTT TAACTTGCTATTTCTAGCTCTAAAAC
11.	Arm_nextgenseq_step1_F	CTCAACGATGAAGATCAAGTGG
12.	Arm_nextgenseq_step1_R	CACAGATTCCACTGGTGAGG
13.	Arm_nextgenseq_step2_F	ACACTCTTTCCCTACACGACGCTCTTCCGATCT CTCAACGATGAAGATCAAGTGG
14.	Arm_nextgenseq_step2_R	GTGACTGGAGTTCAGACGTGTGCTCTTCCGATCT CACAGATTCCACTGGTGAGG
15.	Arm_nextgenseq_step3_F	AATGATACGGCGACCACCGAGATCTACACtatagcctACACTCTTTCCCTACACGAC
16.	Arm_nextgenseq_step3_R	CAAGCAGAAGACGGCATACGAGATacgaattcGTGACTGGAGTTCAGACGTGT
17.	Fz4 primers_F	GATAAGGTCGCGTTACCGATAGG
18.	Fz4 primers_R	GAGTACACGCGAAGCTCAACGC
19.	Wnt7 primers_F	GTCACAATTAGCACAATTATTCAC
20.	Wnt7 primers_R	CGTGAATTTGGGTGAGATTCTTTG
21.	WntA_seq_F	GCGGCCTTCCCCTAATTACA
22.	WntA_seq_R	GACACTCGAGCGTAACACCA

Table S2. CRISPR-Cas9 injection table

Sl. No.	Gene	Number of embryos injected	Number of adults examined	Differentiation of two eyespot centers	Venation dependent eyespot splits	Wing margin disruptions	Scale cell polarity disruptions	Central symmetry system disruptions
**1.**	*arm*	685	24	3	-	4	-	-
**2.**	*fz4*	742	124	18	-	5	2	-
**3.**	*wnt7*	487	75	-	12	-	-	-
**4.**	*wntA*	460	40	-	-	-	-	28

Table S3. Immunostaining Buffers

**Buffers**	**Chemicals**	**Amount**
Fix buffer (30 ml)	M PIPES pH 6.9 (500 mM)	6 ml
	mM EGTA pH 6.9 (500mM)	60 µl
	% Triton x-100 (20 %)	1.5 ml
	mM $MgSO_4$ (1M)	60 µl
	37% Formaldehyde	55 µl per 500 µl of buffer
	dH_2O	22.4 ml
Block buffer (40 ml)	50 mM Tris pH 6.8 (1 M)	2 ml
	150 mM NaCl (5 M)	1.2 ml
	0.5% IGEPAL (NP40) (20%)	1 ml
	5 mg/ml BSA	0.2 gr
	H2O	35.8 ml
Wash buffer (200 ml)	50mM Tris pH 6.8 (1 M)	10 ml
	150 mM NaCl (5 M)	6 ml
	0.5% IGEPAL (20 %)	5 ml
	1 mg/ml BSA	0.2 gr
	dH_2O	179 ml
Mounting media	Tris-HCl (pH 8.0)	20 mM
	N-propyl gallate	0.5%
	Glycerol	60%

Table S4. Chromogenic *in-situ* hybridization Buffers

Buffers	Chemicals	Amount
10X PBS (500 ml)<br>* Sterilize by autoclaving.	K_2HPO_4	5.34 g
	KH_2PO_4	2.64 g
	NaCl	40.9 g
	DEPC treated H_2O	To 500 ml
1X PBST (50 ml)	1X PBS	50 ml
	Tween® 20	50 µl
20X SSC (1000 ml)<br>*Adjust the pH to 7.0 with 1M HCl and sterilize by autoclaving.	NaCl	175.3 g
	Trisodium citrate	88.2 g
	DEPC treated H_2O	Till 1000 ml
Pre-hybridization buffer (40 ml)	Formamide	20 ml
	20X SSC	10 ml
	DEPC treated water	10 ml
	TWEEN20	40 µl
Hybridization buffer (40 ml)	Formamide	20 ml
	20X SSC	10 ml
	DEPC treated water	10 ml
	TWEEN20	40 µl
	Salmon sperm	40 µl
	Glycine (100mg/ml)	40 µl
Block buffer (50 ml)	1X PBS	50 ml
	TWEEN20	50 µl
	BSA	0.1 gm
Alkaline phosphatase buffer (20 ml)	Tris-HCl (pH 8.0)	2 ml
	NaCl (5M)	400 µl
	$MgCl_2$ (200mM)	250 µl
	DEPC treated water	Till 20 ml
	TWEEN20	20 µl

Region of *armadillo* used for CRISPR-Cas9 (Bold, PAM sequence underlined)

ATGAGTTACCAGATACCATCCTCACAGAGCCGTACGATGTCACACAGCAACTATGGTGG
CTCTGACGTGCCAATGGCACCCAGCAAGGAACAACAGACACTCATGTGGCAGCAGAACT
CATACCTGGTGGATTCTGGTATCAACTCTGGGGCAGCCACTCAGGTGCCATCTCTCACTG
GCAAAGAAGATGACGAGATGGAAGGAGATCAGCTCATGTTTGATCTGGACCAGGGCTTT
GCTCAAGGGTTCACTCAGGAGCAAGTGGATGACATGAACCAGCAGTTATCACAAACCAG
GTCCCAGCGTGTGCGCGCTGCTATGTTCCCAGAGACTCTGGAGGAGGGCATTGAAATCCC
CTCCACACAGCTGGACCCAGCGCAGCCTACTGCAGTCCAGCGCTTGTCTGAGCCCTCTCA
GATGTTGAAACATGCTGTTGTCAATCTTATTAATTATCAAGATGATGCTGATTTGGCAAC
AAGGGCAATCCCAGAGTTGATCAAACTACTCAACGATGAAGATCAAGTGGTAGTGTCAC
AAGCTGCCATGATGGTTCACCAGTTGTCAAAGAAGGAGGCTTCGCGGCATGCTATAATG
AACTCTCCCCAAATGGTTGCGGCCTTAGTGCGAGCTATCTCCAACAGCAATGACTTGGAG
ACAACCAAAGGGGCAGTTGGGA**<u>CCT</u>TGCACAACTTGTCTCACCAC**CGACAAGGTCTTC
TTGCCATATTCAAGAGTGGAGGCATACCAGCTCTGGTGAAACTCCTATCCTCACCAGTGG
AATCTGTGTTGTTCTATGCCATTACGACCTTGCATAACCTTTTACTGCACCAAGATGGTTC
AAAAATGGCTGTCCGTCTTGCTGGTGGTCTTCAAAAGATGGTGGCCTTACTTCAGAGGAA
TAATGTGAAGTTCCTGGCAATTGTTACCGATTGTCTCCAGATATTGGCGTATGGGAACCA
GGAATCCAAGCTTATCATCCTGGCTTCACAAGGCCCTATCGAGTTAGTGCGTATCATGCG
CTCTTTTGACTACGAGAAGTTGCTTTGGACCACATCCAGGGTTCTGAAGGTTTTGTCCGTC
TGCTCAAGCAACAAGCCGGCGATAGTGGAGGCAGGTGGTATGCAGGCCCTGGCCATGCA
CCTTGGCAACCCCAGTGGCCGCTTAGTCCAGAATTGCCTTTGGACTTTGAGGAATCTGTC
TGATGCTGCTACCAAGGTGGAAGGGCTGGAAGGCCTGCTGCAAAGCCTAGTGCAAGTGC
TGGCCTCCACCGACGTGAACATCGTGACTTGCGCGGCCGGCATACTGTCCAACTTGACCT
GCAACAACCAACGTAACAAGGTGACAGTGTGCCAAGCCGGCGGCGTGGACGCCCTGGTG
CGCACGGTGGTGTCGGCCGGGGACCGCGAGGAGATCACCGAGCCGGCCGTGTGCGCGCT
CCGCCACCTCACCTCGCGGCACGTCGAGAGCGAGATGGCGCAGAACGCTGTCCGGCTGC
ATTACGGCCTGCCTGTGATAGTGAAACTTCTGCAACCGCCGTCCCGCTGGCCGCTGGTGA
AGGCGGTGGTGGGTCTGGTGCGCAACCTGGCGCTGTGCCCCGCCAACCACGCGCCGCTG
CGCGAACACGGCGCCGTGCACCACCTCGTGCGCCTGCTGCTGCGCGCCTTCAACGACACA
CAGCGGCAACGCGGCTCGGTGTCGGGCGGCGGCGGCGCGGGCGGCGCGTACGCGGACG
GCGTGCGCATGGAGGAGATCGTGGAGGGCGCGGTGGGCGCGCTGCACATCCTGGCGCGC
GAGGGGCTCAACCGCGCGCTCATCCGCCAGCAGAACGTCATACCGATCTTTGTGCAGCTG
CTGTTCAACGAGATCGAGAACATACAGCGCGTAGCAGCAGGGGTACTTTGCGAGTTGGC
AGCAGACAAAGAAGGGGCCGAAATGATAGAAGCCGAAGGGGCTACGGCCCCTCTCACA
GAGTTGCTGCACTCCCGTAATGAAGGCGTAGCGACGTACGCCGCGGCCGTACTGTTCCGC
ATGTCCGAGGACAAGCCGCATGACTACAAGAAGAGACTCTCCATGGAGCTGACCAATTC
ACTCTTCCGCGACGACCACCAGATGTGGCCCAACGATCTGGCCATGCAACCTGATCTGCA
GGACATGCTCGGGCCCGAGCAGGGCTATGAGGGTCTCTACGGGACGCGGCCCTCCTTTC
ATCAGCAAGGCTACGATCAGATACCGATAGATTCAATGCAGGGCCTCGAAATCGGCAGC
GGATTCGGAATGGACATGGATATTGGGGAGGCCGACGGCGGCGGCGCAGCGTCTGCGGA
CCTGGCCTTCCCCGAACCACCGTTAGACAACAACAATGTCGCCGCCTGGTATGACACCGA
CCTTTGA

Region of *frizzled4* used for CRISPR-Cas9 (Bold, PAM sequence underlined)

ATGAAGTGTTTTGTGGTATTAATAACTGTGACCCTCGTCTACGAGATCGTAGCCGAGGCG
TCCGTGAGGACATGTGAACCGATCAAGGTGGCTATGTGCAAGAACATCGGATACAACCA
GACGGGAATGCCCAACTTGGCTCGGCACACTCTCCAAGCGGACGCCGACGTCACACTAC
AGACCTTCAGCCCCCTGGTGCAGTATGGATGCTCGTCCCAGTTGCATTTGTTTTTGTGTGC
GGTGTACGTGCCCATGTGCACTGATAAGGTCGCGTTACCGATAGGTCCGTGTAGGGGTTT
GTGCGAGAGTGTTTACGCCAGATGTTACCCCGTGTTGCGGGGTTTCGGGTTCCCTTGGCC

GGCGGAGTT**GGACTGTTCTCTATTCCCGGCGG**AGAATAACCATGAACACATGTGTATG
GAGGGTCCTGGGGAGCGCGCGCCGCCGATCGGTAC**GATACCGTTGGACACGACGAGG**
GAAGGACGAGGGACTTGCAGACGTTTGGTGAAGCCGAACAGTTGGGTGTGGGTTAGGGG
GTCGGGGCGGTGCGCGCAGTTCTGTGACGCTGAAGTGTTGTGGGAGGTGGGGGAGAGGC
GGGCGGCTGAGGTGTGGCTGGCGACTTGGGCAGCGTTGAGCTTCGCGTGTACTCTTGCGG
CGGTGGCGGCGCAGCTGGCGTGTGGAGCCAGGGGCGGAGCTGGCGAGCGCGCGTTGGTG
CTGGTGGCACTGTGCCGGTGCGCGGCGGCGGCGGGCTGGGGCGTGCGCGCGGCTGCGGG
CCGGACGGCGGCGGGCTGCGCCAAGGACTCCACGTCTCCCACCAGGATGCTGTTAGCGC
ATGACGGCTTAGCGAACCCCAACTGCGCGGTCGTCTTCTTGCTGCTGTATTACTTCGGGC
TGGCTGCGTCAGTTTGGTGA

Region of *Wnt7* used for CRISPR-Cas9 (Bold, PAM sequence underlined)

AAGTCACAATTAGCACAATTATTCACAGATGGTAAAAGACTTAGTGCGACGAGAGTGCAA
GTGTCACGGCGTGTCGGGCTCCTGCGCCTTGCGAACTTGCTGGCGCGCGCTCCCGCCCTT
CCGCGCGGTGGGCGCCGCCCTAC**GCGACAAGTACCACCGAGCGAGG**CTCGTCGCACCGCA
CCCGCCGCCGGCCACGCACGCGCCAC**AGACGCATCTCGTCATACGCAGG**TAGAGTGCACC
CTTGCCGCCCGACGCGACAAAGAATCTCACCCAAATTCACGAACAAAATTTTCTGCTATT
T

Region of *WntA* used for CRISPR-Cas9 (Bold, PAM sequence underlined)

ATGGATGACATAAACAAGATCCCTTCCCCCCATAAACAAGTTGAATGCGCTTCCCCGATGCCGCAG
TCTCTGCACGTGCGGCACTCAAGGAATTTAGCAGCACCGAATAGGCCTGTACAATCGTCTAACACC
TCTGTGGAAACCTTTACAATACTGCACAAAGAAAGCTGCCATAGATTAGAGTATCTCGTCGAACGA
CAAAAGCAATTATGTATGCTTTCTGATAAAATGGTACAGGTGATACAAACAGGAGCGCAACAGGC
AATTGATGAATGTCAGCATCAATTTCGGAATAGCCGTTGGAACTGTAGTACCGTCGACAATTCCAC
TGATATATTCGGCGGAGTGCTAAAATTTAAATCTCGCGAGTCTGCATTCGTCCA**CGCTCTGTCAG**
CAGCAGCATTGGCTCACACAGTTGCTCGCGCGTGCAGTCGGGGCGAACTAAACGAGTGTTCCTGT
GACGCTCGTGTTAGAAAGCGAA**ACGCCGCGGCATTGGCAGTGGGG**TGGTTGTTCTGAGGATATAA
GATATGGAGAAAAGTTCAGTCGTGACTTTGTAGATGCTAAAGAAGACAAGGATAATGATGAAGGT
CTCATGAACTTACATAACAATGAAGCTGGCCGCAGAGCAGTCCGCGGCAGGATGCAGCGCGTGTG
CAAATGCCACGGCATGTCGGGCTCGTGCTCCGTGCGCGTGTGCTGGCGCCGCCTGCCGCAGCTGCG
GCTGGTGGGCGACGTGCTGAGCACCAGATACGAGGGCGCCTCTCATGTTAAGGTTGTAGAGAGGA
AGAGAGGCAAGAATATAAGAAAACTGCGACCGCTGCATCCTGATATAAAGAAACCGAACAAAAC
CGATCTAGTCTATCTCGAGGACTCTCCCGATTACTGTGAACCGAACGACGAGTAA

Sequence of *Wnt1* used for enzyme based *in-situ* hybridization

CAGCAGCTGGATTTTGTCAGTCCAGCTAGGAAGGGGGGCATAGCAAAGGCAGGCGAACC
AAATAACTTATCACCCTTGTCTCCAAGTGTCCTATACATGGACCCGGCTGTTCACGCCAC
CTTGAGGAGGAAACAGAGAAGGCTAGCGAGGGAGAACCCTGGGGTCCTCGCAGCAATA
TCCAAGGGAGCCAGCATGGCTGTGGCCGAATGCCAGCATCAGTTCAAATACAGGAGATG
GAACTGTTCTACAAGAAATTTTTTGCGAGGGAAGAATCTATTTGGAAAAATTGTTGACAG
AGTTTCGCCGGACAAAGCCCCCCCGGCCGGGGCGGCTATAATTACTAATATACACGTCG
ACACGCCATTGACGATTGACGCGACATCTTCATTTCATTGTGGTGTAAACCTCAAGGATC
GCATT AACACGGACGATGACAACGGCACAATA

Sequences and primers used for fluorescent *in-situ* hybridization (HCR3.0)

>Wnt1_XM_024099417.1_ncbi
CAGCAGCTGGATTTTGTCAGTCCAGCTAGGAAGGGGGGCATAGCAAAGGCAGGCGAACC
AAATAACTTATCACCCTTGTCTCCAAGTGTCCTATACATGGACCCGGCTGTTCACGCCAC

CTTGAGGAGGAAACAGAGAAGGCTAGCGAGGGAGAACCCTGGGGTCCTCGCAGCAATA
TCCAAGGGAGCCAGCATGGCTGTGGCCGAATGCCAGCATCAGTTCAAATACAGGAGATG
GAACTGTTCTACAAGAAATTTTTTGCGAGGGAAGAATCTATTTGGAAAAATTGTTGACAG
AGTTTCGCCGGACAAAGCCCCCCCGGCCGGGGCGGCTATAATTACTAATATACACGTCG
ACACGCCATTGACGATTGACGCGACATCTTCATTTCATTGTGGTGTAAACCTCAAGGATC
GCATTAACACGGACGATGACAACGGCACAATA

Wnt11_HCR_P1B1: gAggAgggCAgCAAACggAACCTAGCTGGACTGACAAAATCCAGC
Wnt11_HCR_P2B1: TTCGCCTGCCTTTGCTATGCCCCCCTAgAAgAgTCTTCCTTTACg
Wnt12_HCR_P1B1: gAggAgggCAgCAAACggAAGTATAGGACACTTGGAGACAAGGGT
Wnt12_HCR_P2B1: CAAGGTGGCGTGAACAGCCGGGTCCTAgAAgAgTCTTCCTTTACg
Wnt13_HCR_P1B1: gAggAgggCAgCAAACggAACCCAGGGTTCTCCCTCGCTAGCCTT
Wnt13_HCR_P2B1: GGCTCCCTTGGATATTGCTGCGAGGTAgAAgAgTCTTCCTTTACg
Wnt14_HCR_P1B1: gAggAgggCAgCAAACggAATATTTGAACTGATGCTGGCATTCGG
Wnt14_HCR_P2B1: TTTCTTGTAGAACAGTTCCATCTCCTAgAAgAgTCTTCCTTTACg
Wnt15_HCR_P1B1: gAggAgggCAgCAAACggAAGTCAACAATTTTTCCAAATAGATTC
Wnt15_HCR_P2B1: CGGGGGGGCTTTGTCCGGCGAAACTTAgAAgAgTCTTCCTTTACg
Wnt16_HCR_P1B1: gAggAgggCAgCAAACggAAGCGTGTCGACGTGTATATTAGTAAT
Wnt16_HCR_P2B1: ATGAAGATGTCGCGTCAATCGTCAATAgAAgAgTCTTCCTTTACg

>Wnt5_XM_024081550.1_ncbi
ATGGGCCTCATAGAGTTCAAGGAATGGTCGAATCAGACGGTGGTATTCTCCGGAAAATC
ACGGGTGTGTGCCCACCTGCGCGGCCTGACTCCGGGCCAGCGGCGCGTGTGCCGCCGAC
ACAAGGACCACATGCCGGCGGTCGGCGACGGCGTGCTGCGCGGCATCAGGGAGTGCCAG
CACCAGTTCCGGCACAAGCGGTGGAACTGCACCGTCACTGCTGACGAAACTGTCTTTGGG
CCCTTGACATTGATTGCGTCAAGAGAAACCGCGTTCACCCACGCGATCACAGCGGCGGG
CGTGTCTCTGGAGATCAGCCGCGCGTGTCGCGACGGACGGCTGGCGTCGTGCGGTTGCA
GCCGCGCGGCTCGCCCGCGGCATTTGCACTCCGACTGGGTGTGGGGCGGCTGTGGGGAC
AACCTCGAGTATGGATACAAATTCACGGAAGGTTTCGTGGACATTCGGGAAAGGGAACG
CAAAGTGAAAAGAGGCAGCCGCGAACAGGGCAGGCAGCTTATGAATAGACACAACAAT
GAAGCTGGCAGGCGGGCTGTCATAAAGAAGTCGCGAGTCACGTGCAAGTGCCACGGCGT
GTCCGGCTCCTGCAGCCTCATCACCTGCTGGCAACAGTTGGCCACATTCAGAGAGATCGG
AGACTATCTACGCGATAAATATGAAGGTGCCACGGAGGTGAAGGTGTCTCGACGTGGAA
AACTGAGGCTGAGCAACCCAAACTACAGTCTACCCACTGCGCAAGATCTCGTATATTTGG
AGGACTCACCAAATTACTGCATTCGAAATATTTCTTTGGGTGCACCTGGAACAATGGGCA
GAGAGTGCAACAAAACTTCAGCAGGCATTGACGGGTGCTCACTAATGTGCTGCGGACGG
GGCTACAACACCAAGAAGATTGTAGTTAAAGAGAGATGCGAGTGCAAGTTCCACTGGTG
CTGCCGTGTGGATTGCAATACTTGTGTTACCACTATGGAAGTGTATACTTGTAAATAA

Wnt51_HCR_P1B1: gAggAgggCAgCAAACggAAGCAGGTGGGCACACACCCGTGATTT
Wnt51_HCR_P2B1: CGCGCCGCTGGCCCGGAGTCAGGCCTAgAAgAgTCTTCCTTTACg
Wnt52_HCR_P1B1: gAggAgggCAgCAAACggAAGCCGGCATGTGGTCCTTGTGTCGGC
Wnt52_HCR_P2B1: ATGCCGCGCAGCACGCCGTCGCCGATAgAAgAgTCTTCCTTTACg
Wnt53_HCR_P1B1: gAggAgggCAgCAAACggAAACGCGCGGCTGATCTCCAGAGACAC
Wnt53_HCR_P2B1: CGCACGACGCCAGCCGTCCGTCGCGTAgAAgAgTCTTCCTTTACg
Wnt54_HCR_P1B1: gAggAgggCAgCAAACggAAGTGCAAATGCCGCGGGCGAGCCGCG
Wnt54_HCR_P2B1: CCCACAGCCGCCCCACACCCAGTCGTAgAAgAgTCTTCCTTTACg
Wnt55_HCR_P1B1: gAggAgggCAgCAAACggAATTGCGCAGTGGGTAGACTGTAGTTT
Wnt55_HCR_P2B1: TGGTGAGTCCTCCAAATATACGAGATAgAAgAgTCTTCCTTTACg
Wnt56_HCR_P1B1: gAggAgggCAgCAAACggAAGTTCCAGGTGCACCCAAAGAAATAT
Wnt56_HCR_P2B1: GAAGTTTTGTTGCACTCTCTGCCCATAgAAgAgTCTTCCTTTACg
Wnt57_HCR_P1B1: gAggAgggCAgCAAACggAACCGTCCGCAGCACATTAGTGAGCAC
Wnt57_HCR_P2B1: AACTACAATCTTCTTGGTGTTGTAGTAgAAgAgTCTTCCTTTACg

Wnt58_HCR_P1B1: gAggAgggCAgCAAACggAACGGCAGCACCAGTGGAACTTGCACT
Wnt58_HCR_P2B1: GTGGTAACACAAGTATTGCAATCCATAgAAgAgTCTTCCTTTACg

>Wnt6_XM_024099418.1_ncbi
ATGGATAATACAAGAGAGACGGGGTTTGTGAACGCGATCACTGCAGCCGGAGTGACATA
CGCGATCACCCGCGCCTGTACTGCGGGCTCACTGCTCGAGTGCTCATGTGAAAAGGGTGT
ACCAAAACCGCGTCGTGGAAGAACTCAAACGCCCCAGCCCCCAGCACCAACTCAGACAG
AGCAGTGGCAGTGGGGCGGATGCAGTGACAACGTCCGCTTCGGCCTGCAGAAGTCCAGG
GAATTCATGGACAGTAGATACAGGAAGAGGAGCGACATCAAAACGATGATAAAGCTGC
ATAACCACAACGCTGGGAGGTTGGCAATCAAAAATAACATGAAAGTAGACTGTAAATGT
CACGGCCTATCTGGCTCATGCACACTGCGAACTTGTTGGTGGAGAATGCCCACCTTTAGA
GAAGTGGGGGACCGATTGAGAGACAACTTTGAAGGTGCTGCTAAGGTGATCTCAAGTAA
TGATGGCGACAGTTTTATGCCCGAAAGTCCTAACATCAAGCGACCTGGGAAAAAAGATA
TCATATACTCTGAAGAATCACCCGATTTCTGCGGACCTAACATGAAGACAGGGTCACTCG
GCACTGAAGGGCGCCAGTGCAATATAAGTTCTGCGGGAACTGACAGTTGCGATCAACTT
TGTTGTAGAAGAGGGTACATACAAACATCTATAAAGGAGGCTGAAAATTGCAATTGTCA
ATTTAAGTGGTGTTGCGAAGTCATTTGTAAAACATGCTATGTGAAGCGAGACATACAAAC
GTGCCTTTAA

Wnt61_HCR_P1B2: CCTCgTAAATCCTCATCAAACGTTCACAAACCCCGTCTCTCTTGT
Wnt61_HCR_P2B2: CGTATGTCACTCCGGCTGCAGTGATAAATCATCCAgTAAACCgCC
Wnt62_HCR_P1B2: CCTCgTAAATCCTCATCAAACTGTCTGAGTTGGTGCTGGGGGCTG
Wnt62_HCR_P2B2: CACTGCATCCGCCCCACTGCCACTGAAATCATCCAgTAAACCgCC
Wnt63_HCR_P1B2: CCTCgTAAATCCTCATCAAATGCCAACCTCCCAGCGTTGTGGTTA
Wnt63_HCR_P2B2: TACAGTCTACTTTCATGTTATTTTTGAAATCATCCAgTAAACCgCC
Wnt64_HCR_P1B2: CCTCgTAAATCCTCATCAAAAGATCACCTTAGCAGCACCTTCAAA
Wnt64_HCR_P2B2: GCATAAAACTGTCGCCATCATTACTAAATCATCCAgTAAACCgCC
Wnt65_HCR_P1B2: CCTCgTAAATCCTCATCAAATGCACTGGCGCCCTTCAGTGCCGAG
Wnt65_HCR_P2B2: AACTGTCAGTTCCCGCAGAACTTATAAATCATCCAgTAAACCgCC
Wnt66_HCR_P1B2: CCTCgTAAATCCTCATCAAATTTATAGATGTTTGTATGTACCCTC
Wnt66_HCR_P2B2: AATTGACAATTGCAATTTTCAGCCTAAATCATCCAgTAAACCgCC

>Wnt7_XM_024079304.1_ncbi
ATGGTTAGTTGCATAATATCAAGTACGTGCGTTGAAAAATATCCCAACCTTAATGCTAAC
TTCAAGCAGAGGCGTCCCAAAAACGTCCTTTACGATCCAAACGATGACATGACCGACAA
TGCTTCCCGGGCAGCACAATCAGAAACTACACAGCGTCTTCGCCGGCGAAGACGAGGTC
CCCGATATATAACGAACAGCGCGTCGGGCGGAGTGTCTCTGGGCGCTCACCTGGTGTGC
GCGCGCGTGGCCGGCCTCACCGACAAGCAGCGCGCCATGTGCCGCGCCTCGCCCGCCGC
CATCGCCGCTGTGGGCGACGGACTGAGAATGGCGTACACAGAGTGTCGGTCTCAGCTGG
GCGGCTACCGGTGGAACTGCACCGGCATCGGAGACGGGAACGACTTCGGACATGTCATG
CCTTTAGCGACCCGGGAAGCAGCGTTCACGTACGCGATAACGTCGGCCGGAGTCACTCA
CGCGCTGAGCACGGCGTGCGCGCGCGGCGACCTGCCCGCTTGTGGCTGCTCCTCCAACAG
ACGGCGGTCTCCAAGTCCAACGGAGCAGTTCCAGTGGGGCGGGTGCGGGGAGGCGGCGT
ACGGCGCGCGGTTCGCGAGACGCTTCCTGGACTCGCGGGAGATGGAGGCAGACGCGCGC
AGCCTCATGAATCTGCACAACAACCGCGTCGGCAGGAAGATGGTAAAAGACTTAGTGCG
ACGAGAGTGCAAGTGTCACGGCGTGTCGGGCTCCTGCGCCTTGCGAACTTGCTGGCGCGC
GCTCCCGCCCTTCCGCGCGGTGGGCGCCGCCCTACGCGACAAGTACCACCGAGCGAGGC
TCGTCGCACCGCACCCGCCGCCGGCCACGCACGCGCCACAGACGCATCTCGTCATACGC
AGGTCAAGACAAAACGCTGGTGTGGGCAGACAACCCCGCAAGTCCGAGCTAGTGTTCCT
GGACCCGTCGCCGTCGTACTGTGAACCTGATCCTGTCTCTGGTTCCATGGGCACGCATGG
GCGACATTGCAACAGGACCTCAAGGGGAGAGGAAGGCTGTGAAACTCTTTGCTGTGGTC
GCGGCTACAATACTGTGCGGACCGAAGAAGAAACAAAATGTAATTGCCGTTTCCATTGG
TGTTGCCGTGTTTCGTGCGATAAGTGCATCACTCGTACCGAGTTACACGTTTTGCAGTTCC

AACGATTCATTGCTTTGGCGAAGTGCAGAGAACGGACAAATATGTACAAGGCAAAGACA
CCAGCTCCTGCCCCTTCAAAGCCTGCAGCCCCACGTGGCCGAGGCAGACGGAGCATCTTC
AAGAGGCAGCCATTAAAAGCTCCCACTGCCACAGCAACTGTAGTCACAAGTGACTCTTT
GTTTTTAAGGGCTCATACATGCAAGTTGGAGATATAGTATCTATGTTAGATGTAGACGG
TGGCACATTCTATGCTCAGATCAGAGGTTTCCTCACCGACCAGTACTGTGAGAAGAGTGC
GGTAGTGACCTGGCTGTTGCCTACTAAAGCTAGCCCTCCGCCGGAGAAGGGATTTGATCC
AGCTACTTACATTATTGGACCAGAAGAAGATCTACCTCGGAAGCTGGAATATATGGAGTT
TGTAATGCATGCACCGTCTGACTATTATAAAGCAAGCAATAGTCCCTACCCACTCACAGA
CAATGAAGTGAACAATTACAATGGATTCATTTGGACAAGCCTTGAACCTAAAGATAGAA
CATAG

Wnt7_1_P1B3 gTCCCTgCCTCTATATCTTTTCGCTCGGTGGTACTTGTCGCGTAG
Wnt7_2_P1B3 gTCCCTgCCTCTATATCTTTACGCCGCCTCCCCGCACCCGCCCCA
Wnt7_3_P1B3 gTCCCTgCCTCTATATCTTTTGCCGACGCGGTTGTTGTGCAGATT
Wnt7_4_P1B3 gTCCCTgCCTCTATATCTTTTGCCCACACCAGCGTTTTGTCTTGA
Wnt7_5_P1B3 gTCCCTgCCTCTATATCTTTGCGTGCCCATGGAACCAGAGACAGG
Wnt7_6_P1B3 gTCCCTgCCTCTATATCTTTCTTCTTCGGTCCGCACAGTATTGTA
Wnt7_1_P2B3 CCGGCGGCGGGTGCGGTGCGACGAGTTCCACTCAACTTTAACCCg
Wnt7_2_P2B3 GGAAGCGTCTCGCGAACCGCGCGCCTTCCACTCAACTTTAACCCg
Wnt7_3_P2B3 AGTCTTTTACCATCTCGCTCACCTTTTCCACTCAACTTTAACCCg
Wnt7_4_P2B3 ACACTAGCTCGGACTTGCGGGGTTGTTCCACTCAACTTTAACCCg
Wnt7_5_P2B3 TTGAGGTCCTGTTGCAATGTCGCCCTTCCACTCAACTTTAACCCg
Wnt7_6_P2B3 AATGGAAACGGCAATTACATTTTGTTTCCACTCAACTTTAACCCg

>Wnt9_nBa.0.1-t07006-RA_lepbase
ATGAAAATCCACACCCCTTTCGGTTTCTACACGACATCGTACCGAAATGCTAAATCGCTT
GGCGTTTCTATATTGGCTTTGAAAAATGTTGAGGGATCTCGACCGGTTCTAGAGAAGACA
GTTGAAGCCTTATTCGCTGACAGATCTGAAGTTATTCATCCAGCCACGTGTAAAGTATTC
ACATCGTCTACACGTCAGTCAAAAATGTGCAAAAGAGAGCCAGGTTTGCCGAATATACT
AGTCAAAGCGAAACAGCAAGCAATTAAGGCTTGTGAAGAGACTTTTCAATACGACAGAT
GGAACTGTTCACTGGTTTTCAATAAGAAGCCGAAAAGGAGTATTTTCAAAAAGATATAC
AGAGAGACTGCGTTTATACACTCTCTTGTGGCAGCTTCTATCACTCACGCAGTAGCAAAA
GGATGTGCTTCTGGTGAGCTATCCAGATGTTCTTGTATCGGTAGTTTTAGAAACGCCTCG
AACGTTCAAATGAGAGGAGGCTGTGGCGATGATTTTAAATTCGGTAAACGATTTGCTAA
GAACTTTCTCGAATGGAAAACAGCGGGGAACGATCAAATAGCGGATGCTTTAAAACAAG
ATGTCAACATAGGTATTGATTCCATAGGCGAACAGTTGAAAGAAGTTTGCAAATGCCAT
GGATTTTCCGGTTCGTGTACAACGAAGACTTGCTGGAAAAGACTGGGTCCTTTCAACTCA
GCTATGGGTTTATTGAAGAAGCATTACCACCACGCGGTGAAGAAAAAACTAGTGAATTT
TACATCTAAAAGAGCTGTTACGATGTAG

Wnt91_HCR_P1B2: CCTCgTAAATCCTCATCAAACCGGTCGAGATCCCTCAACATTTTT
Wnt91_HCR_P2B2: ATAAGGCTTCAACTGTCTTCTCTAGAAATCATCCAgTAAACCgCC
Wnt92_HCR_P1B2: CCTCgTAAATCCTCATCAAATTTCGCTTTGACTAGTATATTCGGC
Wnt92_HCR_P2B2: CTCTTCACAAGCCTTAATTGCTTGCAAATCATCCAgTAAACCgCC
Wnt93_HCR_P1B2: CCTCgTAAATCCTCATCAAAATAGAAGCTGCCACAAGAGAGTGTA
Wnt93_HCR_P2B2: GCACATCCTTTTGCTACTGCGTGAGAAATCATCCAgTAAACCgCC
Wnt94_HCR_P1B2: CCTCgTAAATCCTCATCAAAAGAAGTTCTTAGCAAATCGTTTAC
Wnt94_HCR_P2B2: TGATCGTTCCCCGCTGTTTTCCATTAAATCATCCAgTAAACCgCC
Wnt95_HCR_P1B2: CCTCgTAAATCCTCATCAAACCAGCAAGTCTTCGTTGTACACGAA
Wnt95_HCR_P2B2: AGCTGAGTTGAAAGGACCCAGTCTTAAATCATCCAgTAAACCgCC

>Wnt10_XM_024099420.1_ncbi
ATGAGGAAGTTGAAAGTTGCAGTTGGAAAACGCAGAATGCACAGGACGTACCCGGGGG
CGATATATTTTGTGCTGATTGCATTTTTTGAGGTTGTGAGCTCAAGAGACAACATGCTGC
CACACCACCTGAAGCTCAGTTCTACTCTAACTTGTCGACTCATCGGTGGTCTGACCAGAG
AACAGAGATCTGTCTGCCACGACGCGGCCGATACAGCAGCCATCGCCTTCGAGGGTCTG
CAGATGGCGGTCAAGGAGTGCCAGCATCAGTTCCGCTGGCACAGGTGGAACTGCTCCAG
TCTGCTGGTCAAGAGTTCCAATCCTCACGCCAGTGCTATTATGAAGAGAGGATTCCGGGA
AACCGCGTTCCTGTACGCCCTAACAGCGGCAGGAGTAGCTCACGCAGTGGCCCGGGCGT
GCGCCCAGGGCCGGCTCATATCCTGCGGCTGCGACCCCCTGGGGTACCGCGCAGCCCAT
GAGAGGGGCCGCACGAGGACCAACAAGTGGGAGTGGAGTGGCTGCTCCCACAACCTGG
CCTATGGCGTCGAGTTCTCCAAGAAATTCCTCGATGTACGGGAAAAGGTGGACGATCTGC
AGTCGAAGATCAACGTACATAATAACAATGCTGGTAGATCGATTCTATCATCTCACATGG
AGGTGCGGTGCAAGTGCCACGGGCTGTCAGGAAGTTGTCAACTGCGAACGTGTTGGCGC
GCCACGCCCGACTTCAGGGCTGTGGCTTCTACTATTAAGAGACAATACCGCAAAGCTTTA
GTAGTAGCCCAAGAAGAGCTCAATAACAGCCCTTCAGTGTTACGAGGGCGGCCACGAGG
AAGAAGGAGGAGTCGAGCAAGACCTGCACCGAAGTCTAGCTTGCTGTTTTTGAGAAGT
CCCCAAGTTTTTGTGAAGCAGACCCCAAATTTGATTCCGCGGGTACATCAGGAAGAGTCT
GCCGCATCGGAAGGACAACAAGGACTGGATCCTGTGACCTGCTGTGCTGTGGACGAGGA
CACGCCCTCATCAGAAAGTCAAGTATCAAACCATGTAACTGCACCTTTCACTGGTGCTGT
AGAGTCGATTGCCAGAGGTGCCAGGATGATAAATGGATTTCAATTTGCAAGTAA

Wnt101_HCR_P1B2: CCTCgTAAATCCTCATCAAACACAACCTCAAAAAATGCAATCAGC
Wnt101_HCR_P2B2: GTGTGGCAGCATGTTGTCTCTTGAGAAATCATCCAgTAAACCgCC
Wnt102_HCR_P1B2: CCTCgTAAATCCTCATCAAACCGATGAGTCGACAAGTTAGAGTAG
Wnt102_HCR_P2B2: ACAGATCTCTGTTCTCTGGTCAGACAAATCATCCAgTAAACCgCC
Wnt103_HCR_P1B2: CCTCgTAAATCCTCATCAAACTGGAGCAGTTCCACCTGTGCCAGC
Wnt103_HCR_P2B2: TGAGGATTGGAACTCTTGACCAGCAAAATCATCCAgTAAACCgCC
Wnt104_HCR_P1B2: CCTCgTAAATCCTCATCAAACGCGGTTTCCCGGAATCCTCTCTTC
Wnt104_HCR_P2B2: TCCTGCCGCTGTTAGGGCGTACAGGAAATCATCCAgTAAACCgCC
Wnt105_HCR_P1B2: CCTCgTAAATCCTCATCAAAGTCCTCGTGCGGCCCCTCTCATGGG
Wnt105_HCR_P2B2: GAGCAGCCACTCCACTCCCACTTGTAAATCATCCAgTAAACCgCC
Wnt106_HCR_P1B2: CCTCgTAAATCCTCATCAAAAATTTCTTGGAGAACTCGACGCCAT
Wnt106_HCR_P2B2: TCGTCCACCTTTTCCCGTACATCGAAAATCATCCAgTAAACCgCC

>Wnt11_XM_024078736.1_ncbi
ATGCGTTTATTTATTGTGATTTTTATTTTGTTCTTTGTGTTTTTACCTAAATCTACTAAGGC
GATTCGATGGCTAGCGCTTCATGAGAACGAAGGCAACTGGACGGAGGCGGAGTGCGGCG
ACGCGCGGCGGCGGGGCGAGCTGTGGGCGGGCAGGACGCGCGTGTGCCGCCGCCAGCC
GGCAGCCATGCCGCACGTGGGCAGCCGCCGCGCCCTGGCCCGCGCAGCCTGCCTCGCCG
CGCACGCCGGCGAGCGCTGGAACTGCAGTTCCATCGATNTCGCGCCGAGATACACGCCC
GACTTACTCACAGGTTCTCGAAGCAAGCCTTACGTTGTATGCGATGTCGGCGCGGCGCTT
GCGTGGTCAGTTTTGAGCGCAGCGCGTGCGCGTCGGGGCGCGCTGGCCGCGTGCTCGTGC
GCCGCCCCGCCGCGTGTCCGCCGCGCCCGCCCGCGCCAGGGCCGCGCGCCCGAGCCGCA
CGCGCGGTTCAAAGTGGGGCGGACTGCGGAGACAAACTAATTCAGTGCGGCTGAGAGAT
TCGCGAAACAATTCTTGGACACACACGAGATAGACGTGCGAGACGGTAGAATAGAAGAC
GATATCATCGAGTGGGAGCCGACCACCGAGAAGACCACCACCCTGGAGCCGACCACGAT
GCCTCCCGTCGTCATATTGGTGGACGACCAGCCGGCGCCGCCGAACACCACCGCACCTCC
GAGGAAAAAAGGTCGACGGGGACGCAAACGTTTGCCGAGGTCACCGAGACGAGGACGG
CCCACAAGGAAAAGATTCAGATCCAGATACGACTACGATAACGAGAAAGAATTTGAACA
TCGAAACATAGAGTACCGCATGGCGGCCGACGACCCGCGCTTCGATCCTCAGGTAGACC
TGCACACTCGGCTGCTACATCTGCGGCCGCTCATTGCTGCTGCCAACCTCATCAATAGCC

GCTTCGGAAGAAAGGTGGTATCGCAAGGCATGCGCACCAAGTGCACATGTCACGGCGTG
TCGGGCTCGTGCTCCGTGCGCACCTGCTGGCGCGCGCTGACGCCGCTGGCGCGCGCGGCC
GAGGCCCTGGCGCATGAAGCCGCGCGCGCTGCTCCCCTTGCGCCGCGCACCCCGCCGCG
CCGCCACCGCCGCACGAAGACGCGGCTGCGCTACGTCACGCCCAGCCCAGATTACTGCG
AGCCCGATCCCGCCGCTGGTTCATTGGGCACACACGGCAGGAAGTGCAACGCGACGCTA
GGCGGGGCAGCGGGCGGGTGTGGGCGGCTGTGCTGCGGGCGCGGGCGGCGCGCGGTGC
GCTCGGCGCGGCTGGAGCGCTGCCGTTGCCGCTACCACTGGTGCTGCCGCGTCGACTGCC
AGCTGTGCCGCGTTACCAGCGAGGACCACTATTGCAACTAG

Wnt111_HCR_P1B2: CCTCgTAAATCCTCATCAAACCTCCGTCCAGTTGCCTTCGTTCTC
Wnt111_HCR_P2B2: CCCGCCGCCGCGCGTCGCCGCACTCAAATCATCCAgTAAACCgCC
Wnt112_HCR_P1B2: CCTCgTAAATCCTCATCAAAGCCGGCGTGCGCGGCGAGGCAGGCT
Wnt112_HCR_P2B2: ATCGATGGAACTGCAGTTCCAGCGCAAATCATCCAgTAAACCgCC
Wnt113_HCR_P1B2: CCTCgTAAATCCTCATCAAACCGACGCGCACGCGCTGCGCTCAAA
Wnt113_HCR_P2B2: GGCGCACGAGCACGCGGCCAGCGCGAAATCATCCAgTAAACCgCC
Wnt114_HCR_P1B2: CCTCgTAAATCCTCATCAAAAATCTCTCAGCCGCACTGAATTAGT
Wnt114_HCR_P2B2: TCGTGTGTGTCCAAGAATTGTTTCGAAATCATCCAgTAAACCgCC
Wnt115_HCR_P1B2: CCTCgTAAATCCTCATCAAACGTCCACCAATATGACGACGGGAGG
Wnt115_HCR_P2B2: CGGTGGTGTTCGGCGGCGCCGGCTGAAATCATCCAgTAAACCgCC
Wnt116_HCR_P1B2: CCTCgTAAATCCTCATCAAATTCTTTCTCGTTATCGTAGTCGTAT
Wnt116_HCR_P2B2: GCGGTACTCTATGTTTCGATGTTCAAAATCATCCAgTAAACCgCC
Wnt117_HCR_P1B2: CCTCgTAAATCCTCATCAAAACCACCTTTCTTCCGAAGCGGCTAT
Wnt117_HCR_P2B2: GTGCACTTGGTGCGCATGCCTTGCGAAATCATCCAgTAAACCgCC

>frizzled4_XM_024081478.1_ncbi
ATGAAGTGTTTTGTGGTATTAATAACTGTGACCCTCGTCTACGAGATCGTAGCCGAGGCG
TCCGTGAGGACATGTGAACCGATCAAGGTGGCTATGTGCAAGAACATCGGATACAACCA
GACGGGAATGCCCAACTTGGCTCGGCACACTCTCCAAGCGGACGCCGACGTCACACTAC
AGACCTTCAGCCCCCTGGTGCAGTATGGATGCTCGTCCCAGTTGCATTTGTTTTGTGTGC
GGTGTACGTGCCCATGTGCACTGATAAGGTCGCGTTACCGATAGGTCCGTGTAGGGGTTT
GTGCGAGAGTGTTTACGCCAGATGTTACCCCGTGTTGCGGGGTTTCGGGTTCCCTTGGCC
GGCGGAGTTGGACTGTTCTCTATTCCCGGCGGAGAATAACCATGAACACATGTGTATGGA
GGGTCCTGGGGAGCGCGCGCCGCCGATCGGTACGATACCGTTGGACACGACGAGGGAAG
GACGAGGGACTTGCAGACGTTTGGTGAAGCCGAACAGTTGGGTGTGGGTTAGGGGGTCG
GGGCGGTGCGCGCAGTTCTGTGACGCTGAAGTGTTGTGGGAGGTGGGGGAGAGGCGGGC
GGCTGAGGTGTGGCTGGCGACTTGGGCAGCGTTGAGCTTCGCGTGTACTCTTGCGGCGGT
GGCGGCGCAGCTGGCGTGTGGAGCCAGGGGCGGAGCTGGCGAGCGCGCGTTGGTGCTGG
TGGCACTGTGCCGGTGCGCGGCGGCGGCGGGCTGGGGCGTGCGCGCGGCTGCGGGCCGG
ACGGCGGCGGGCTGCGCCAAGGACTCCACGTCTCCCACCAGGATGCTGTTAGCGCATGA
CGGCTTAGCGAACCCCAACTGCGCGGTCGTCTTCTTGCTGCTGTATTACTTCGGGCTGGCT
GCGTCAGTTTGGTGGGTGGTAGTGACGGGTGCGTGGCGAGCGAGCGTGCTGCGCCCCCC
AGCCAGTAGCGGCGCCAGGAACGACCGCCACTCCTCCCTGCTGCAGCTGGCTGCGTGGG
GCGTGCCCGCCGCGCTGGCCGCCGCCGTGCTGGTCACAAGGGACGTGGATGCTGATGAG
CTTACAGGCACATGCTTCGTGGGCAATCAGTCTAGCAAATCTTTGCTAGCGTTGGTTATC
GTTCCCGAAGCGATATGCTTGCTTCTGGGCAGCGTGTTCCTCGCTTCCGGCCTTCGCGCA
GTGCTTCGTAAACCTGTACCGATTCCAGCTCCGGCAACGCTTCTGAATTCTGCACCGCAA
GCGCACCCTGATCAGAGTCTGCTGAGGCTAGGAGCCTTCGCTGCGTTGTACGCTGTGCCA
TCTGCCTGCATACTGGCGACATGGGTGTATGAATATATTCTGAGAGAAAATTGGTTGGCT
GCCCCCGTCCCTTCGACGGAACCTTCGACGCAACCGCGTCCAGCATTCTGGGTGTTCCTT
TTTAGGATATTCGCATCACAAATACTGGGTGTCATGGTGGCGGTTTGGATAGCCACGCCA
CGGTTGAAGGCGCTGTGGAGGAGAATCAGTGGGCCAAGAAAACCAGCGTTAGCAAAAT
GCCCACCTGGTCCGACGCCTACACCGTTAACATTGCATTGCTACGCTACGCATCCGCATA
CGTTGACGAGACACCCACAGAAATACGCCACATACCGACCACCACAGCAACAGTCGTAT
AGAAAACCTCGACATTATCATTATTCTGCTGGAGAAACTATCCTATGA

Fz4_1_P1B2 CCTCgTAAATCCTCATCAAATGATCGGTTCACATGTCCTCACGGA
Fz4_2_P1B2 CCTCgTAAATCCTCATCAAACATACTGCACCAGGGGGCTGAAGGT
Fz4_3_P1B2 CCTCgTAAATCCTCATCAAAAACATCTGGCGTAAACACTCTCGCA
Fz4_4_P1B2 CCTCgTAAATCCTCATCAAACAGCGTCACAGAACTGCGCGCACCG
Fz4_5_P1B2 CCTCgTAAATCCTCATCAAACCCTGGCTCCACACGCCAGCTGCGC
Fz4_6_P1B2 CCTCgTAAATCCTCATCAAAACGTGGAGTCCTTGGCGCAGCCCGC
Fz4_1_P2B2 ATCCGATGTTCTTGCACATAGCCACAAATCATCCAgTAAACCgCC
Fz4_2_P2B2 AAAACAAATGCAACTGGGACGAGCAAAATCATCCAgTAAACCgCC
Fz4_3_P2B2 GGAACCCGAAACCCCGCAACACGGGAAATCATCCAgTAAACCgCC
Fz4_4_P2B2 GCCTCTCCCCCACCTCCCACAACACAAATCATCCAgTAAACCgCC
Fz4_5_P2B2 GCACCAACGCGCGCTCGCCAGCTCCAAATCATCCAgTAAACCgCC
Fz4_6_P2B2 CATGCGCTAACAGCATCCTGGTGGGAAATCATCCAgTAAACCgCC

>frizzled2_XM_024097661.1_ncbi
ATGAAAAAAGAAAAACCGACGTTTATGAGGCTGTTGTGTACAATTTTGAATCCGCGGCTC
GTGGCGCTGCGCGCGCGCAACGCCAGCGCGCCCGGCGTGCCGGCGCCCGCCTGCGGCCT
GCCGTGCCGCGGCGCCTTCTTCTCGCGCGAGGAGAAGGAGTTCGCCGCCGTGTGGGTCGC
GCTGTGGGCCGGCCTGTGCGCCGCCTCCACGCTCATGACGCTCACCACCTTCGCCATCGA
CTCGCAGCGCTTCAAGTACCCCGAGCGGCCCATCGTGTACCTGTCGGCCTGCTACTTCAT
GGTGGCGCTGGGCTACCTGGCGCGCCTGGCGCTGGGCCACGAGGCCGTGGCGTGTGACG
GGCCGCTGCTGCGGACGTCGGCGGGCGGGCCCGGCGCCTGCACGCTGGTGTTCGTGCTG
GTGTACTTCTTCGGCATGGCGTCGTCCATCTGGTGGGTGGTGCTGTCGTTCGCGTGGTTCC
TGGCCGCCGGGCTCAAGTGGGGCAACGAGGCCATCGCCGGGCACGCGCAGTACTACCAC
CTGGCGGCGTGGCTGGTGCCGGCCGCCAAGACCGTGGCCGTGCTGCTGGCGGGCGCCGT
GGACGGCGACCCCGTGGCCGGCGTGTGCTACGTGGGCAACTCGTCGCCCGAGCACCTGC
GGCGCTACGTGCTGGCGCCGCTCGTGGTGTACTTCGCGCTGGGCGCCTCCTTCCTGCTGG
CCGGCTTCGTGTCGCTGTTCCGCATCCGCAGCGTCATCAAGCGGCAGGGCGGCGCGGGC
GCGGGCTCCAAGGCCGACAAGCTGGAGAAGCTCATGATCCGCATCGGCGTGTTCAGCGT
GCTGTACGCCGTGCCGGCCGGCGTGGTGATCGGCTGCCTGGCGTACGAGGCGGGCGGGC
GCGAGCGCTGGCTGCGGCGCGTGGCGTGCGGCGCGGCGTGCGGCCCGCGCCCCGTCTAC
TCGGCGCTCATGCTCAAGTACTTCATGGCGCTGGCCGTGGGCATCACGTCGGGCGTGTGG
ATCTGGTCGGGCAAGACGCTGGAGTCGTGGCGCCGCGTGTGGCGCGGGGGGCGCGCGCC
GCCGCCCGCGCAGCGCGCGCTG

Ftz21_HCR_P1B4: CCTCAACCTACCTCCAACAAGAACTCCTTCTCCTCGCGCGAGAAG
Ftz21_HCR_P2B4: GGCCCACAGCGCGACCCACACGGCGATTCTCACCATATTCgCTTC
Ftz22_HCR_P1B4: CCTCAACCTACCTCCAACAAAAGTAGCAGGCCGACAGGTACACGA
Ftz22_HCR_P2B4: CGCGCCAGGTAGCCCAGCGCCACCAATTCTCACCATATTCgCTTC
Ftz23_HCR_P1B4: CCTCAACCTACCTCCAACAAAGAAGTACACCAGCACGAACACCAG
Ftz23_HCR_P2B4: CCCACCAGATGGACGACGCCATGCCATTCTCACCATATTCgCTTC
Ftz24_HCR_P1B4: CCTCAACCTACCTCCAACAACCGGCACCAGCCACGCCGCCAGGTG
Ftz24_HCR_P2B4: CCAGCAGCACGGCCACGGTCTTGGCATTCTCACCATATTCgCTTC
Ftz25_HCR_P1B4: CCTCAACCTACCTCCAACAAGAAGTACACCACGAGCGGCGCCAGC
Ftz25_HCR_P2B4: GGCCAGCAGGAAGGAGGCGCCCAGCATTCTCACCATATTCgCTTC
Ftz26_HCR_P1B4: CCTCAACCTACCTCCAACAAGCCGATGCGGATCATGAGCTTCTCC
Ftz26_HCR_P2B4: CGGCACGGCGTACAGCACGCTGAACATTCTCACCATATTCgCTTC

>frizzled_XM_024099445.1_ncbi
ATGGTCTGGCGATGGATTTTGTTTGTATACGCCTTGTCTGTTATAACTAAAAGTTCACGTG
TTACAGTGAACCAAGGGGATACTTTACCTCATCATGGACGATGTGAACCAATAACAATA
CAGTTTTGCCAGCAGATACAGTACAATCAAACAATTTTTCCAAACTTGCTGAACCACGCT
AAACAGGAAGATGCTGGATCCGAAGTACATCAATATACACCTCTTATAAAAGTAAACTG
TTCGCCAGACTTGAAATTCTTTTTGTGCTCAGTTTTTGCACCAGTTTGTACAATATTAAAT
GAACCTATTCCGCCTTGTCGTCATTTATGCGAATCTGCAAGACACAACTGCGATGAGATA

CTTATACATTTTGGATTTGAGTGGCCAAAGGCGTTAGAGTGTTCACGATTTCCAGTGGTT
ACAGATGAAAATGTTATATGTGTTGGGGATAACAATGTGACTCATGAACCTCGTAAGGCT
ACAGAGACCAAACTTCGGAAGGTAGATTACAAGTCAAACAATGATAGAGACCCTACAAA
GTTACAAGGTGCTAAAGACCTTGGTTTTGTTTGTCCAGTACATTTTAAAATACCTAAGAA
TCTGGATTTAGAGTATTCCCTGAAAGTTGGAGATAGAACTGAACGTGACTGTGGGGCACC
ATGCAACGGAATGTTTTTCAGTGAAGAGGAAAAAAGTTTTGCACGATCATGGATTGTTAT
TTGGGCCACTTTGTGCTCAATCAGTTGCTTGTTCACTGTTTTGACATTTGTAATAGATACA
GATAGATTCCGCTATCCAGAGAGGCCTATAATTTTTTTATCAGTGTGTTATCTAATGGTTG
CCGCAGCCTATGTTATGGGATGGGGTGCAGGAGACTCTGTCAGCTGCCAAGGGCCCTTTC
CGTCAACTATTAGTGGAACAAGGTTGCCTAACATATCAGTTATTACACAAGGAACAAAA
CATGAGCCGTGTACAATCCTTTTTATGATTGTATATTTCTTCAGTATGGCATCTAGCATTT
GGTGGGTTATTTTAACACTCACATGGTTTTTAGCAGCAGGATTGAAGTGGGGGCATGAAG
CAATAGAAGCTAATTCACAATATTTTCATCTAGTGGCTTGGGCAGTGCCTGCCATTAAAA
CAATATTTATTCTAGCTATGGGCAAAGTTGATGGTGATGTACTCTCTGGTGTGTGCTATGT
TGGGCTTTGGGATGCAGAAACTTTACGTGGTTTTGTATTGGCACCATTATGTGTTTATTTA
GTCTTGGGCACTATATTTTTAATGGCAGGTTTTGTGTCACTATTCAGAATAAGAACAGTT
ATGAAACATGATGGTACCAAAACTGATAAATTGGAAAAGTTAATGATACGTATTGGTAT
ATTTGGTGTGTTATACACTGTTCCTGCTCTTATTGTGATTTCATGTTTATTTTACGAACAG
GCTTATTTTGACAAATGGATGGTGACATGGCACCGTGACATGTGCTCAACACCTCTCTAC
TCTATTCCATGCCCATTTACACATCAGGAGATGGAAAGGCCTAAATTTGAAGTGTTCATG
ATAAAATATCTTATGACAATGATTGTTGGCATCACATCAAGTTTTTGGATTTGGTCTGGTA
AAACTCTAGTTTCATGGCATCAATTTTTTGATAAAATAAGGGGAAGACGAGTTGAAGCAT
ATGTTTGA

Ftz1_HCR_P1B4: CCTCAACCTACCTCCAACAACCATGATGAGGTAAAGTATCCCCTT
Ftz1_HCR_P2B4: AACTGTATTGTTATTGGTTCACATCATTCTCACCATATTCgCTTC
Ftz2_HCR_P1B4: CCTCAACCTACCTCCAACAACTTTTATAAGAGGTGTATATTGATG
Ftz2_HCR_P2B4: AGAATTTCAAGTCTGGCGAACAGTTATTCTCACCATATTCgCTTC
Ftz3_HCR_P1B4: CCTCAACCTACCTCCAACAAATGTATAAGTATCTCATCGCAGTTG
Ftz3_HCR_P2B4: TAACGCCTTTGGCCACTCAAATCCAATTCTCACCATATTCgCTTC
Ftz4_HCR_P1B4: CCTCAACCTACCTCCAACAAACCTTCCGAAGTTTGGTCTCTGTAG
Ftz4_HCR_P2B4: TCTCTATCATTGTTTGACTTGTAATATTCTCACCATATTCgCTTC
Ftz5_HCR_P1B4: CCTCAACCTACCTCCAACAATTCTATCTCCAACTTTCAGGGAATA
Ftz5_HCR_P2B4: TGCATGGTGCCCCACAGTCACGTTCATTCTCACCATATTCgCTTC

> frizzled9_XM_024087045.1_ncbi
ATGATGGGGAGGATTTTGTGTGTTTTTCTCCTGGTCTGGATGGTCGCTGCTGGCCAGGAG
GAGGAGGGTGGGAAATGCGAGAGGATCACCCTGTCCCAGTGCCAGGATCTGGGGTACAA
TTGGACCGCTATGCCCAATCTCATTGGACATAGAGACCAGAAGGAGGCAGAAGAAGCGA
TGACTGCGTTCACGAGCATCCTTGCGAGCGAATGCTCGGTGCACGCTCGCTTCCTGCTGT
GCTCGGCCTTCGCCCCGCTGTGCTCCGAGCAGGTGTCGGGCTCCGTCAGCGCCTGCCGCG
CGCTCTGCGACAAGGTCGTCCGCGACTGCAAGAACCAGATCGCGGCCCTGCCCGACGGC
ATCAAGCTCGACTGCTCCGCCTTCCCGCTCCGCCCGGACTGGCGGCTCTGCATGCGGCCG
CCCAACGCCAGCGAGGAGCCCGAGCCGCCGCCGGTGCCGCGCTGGCCCTTCAACGAGCA
AGATCTGAAAGAGCACGCGTGTCCCCCGGGCTACGCGCACTCCCCCACGGGCTCGTGCT
GGCCCGCCTGCGACAAGCCCGCGCGCTACACGCAAGCTAACAAGAGAAAAGCGGAGAT
ATGGATGCTCACGCTCGCCTGGTTCTCTCTGCTCTCCACTTCTTTCGCGCTGCTCACGTTC
TGCGCTGAACCGTCGCGATACCGCTACCCCGAGAGGCCCGTCGTCTGGATGGCCGCGTGC
CACGCTGTCGTCGCGCTCGCCTACGTCACGAGAGGCTGGCTCGGTGCGAAGACCGTCTCC
TGCACTGGAAACCTGTTGGCCGTCGACGGAATGGGTTCGACGATTTGCGTCGCATTCTTC
TCTCTTACATACTACTTCACGTTAGCGGCGGACGCGTGGTTCGCGAACGCGTGCGTAGCG

TGGTACTTGACCGCTGCGAGCGAGTGGTCCACCGAGGCGTTGGAGCGGGCGGCGGCGTA
CCTGCACGCGGTGGCGTGGGGCTGGGCGGGGGCGTGGACGGCGGCGGCGCTGGCGCTGC
GGCGGGTGACGGCGGACGAGCTGACCGGGACCTGCGGCGTGTCGGACGAAGCGGCCGC
GGCGCTGATCGGCGTCCCGCGAGGCGCTCTGCTCATCACCTCAATAGCGTTGGCTATCGG
CGCGTGTCCGGGCATAATGAGAGTGCGCCGCGCGTTGGACTCGCGAGGAGCGAAACGAG
TGGGACGGTTGGCCGCTCGAGCGGCCGCTGGAGGCCTGCTCTACCTGTTCTTAGCGGCGT
GCGCGACCGGCGCGCGCGTGATTGAAGCCAGAAACAGAGCGGCGCAAAAGAGCTTGCC
GCTTCTGGCGGCGATGGGCGCGGGCAGTCTGGATGACTCGGCGGGGTCGCGCGCCGTGG
CCGTGAGCCTGTGGCTGTGCTGCGGGGTCGCCGCGGGGGCGTGGTCGTGGTCGCGCAAG
TCGGCGGTGGTGTGGCGCAAGGCGCTGTGTCCGCCGCGCAAGGCCCCGTGCTGCGCGCC
GCCGCTGCTCGGACCCCATCACCCGTACTATAAAAGACCTCTGCACGTGTCTAGAGTGTG
A

Ftz91_HCR_P1B4: CCTCAACCTACCTCCAACAACTGGTCTCTATGTCCAATGAGATTG
Ftz91_HCR_P2B4: CGCAGTCATCGCTTCTTCTGCCTCCATTCTCACCATATTCgCTTC
Ftz92_HCR_P1B4: CCTCAACCTACCTCCAACAAGGCGCTGACGGAGCCCGACACCTG
Ftz92_HCR_P2B4: GGACGACCTTGTCGCAGAGCGCGCGATTCTCACCATATTCgCTTC
Ftz93_HCR_P1B4: CCTCAACCTACCTCCAACAAGGCGTTGGGCGGCCGCATGCAGAGC
Ftz93_HCR_P2B4: CACCGGCGGCGGCTCGGGCTCCTCGATTCTCACCATATTCgCTTC
Ftz94_HCR_P1B4: CCTCAACCTACCTCCAACAAGCGCGGGCTTGTCGCAGGCGGGCCA
Ftz94_HCR_P2B4: CTTTTCTCTTGTTAGCTTGCGTGTAATTCTCACCATATTCgCTTC
Ftz95_HCR_P1B4: CCTCAACCTACCTCCAACAAACGGGCCTCTCGGGGTAGCGGTATC
Ftz95_HCR_P2B4: ACAGCGTGGCACGCGGCCATCCAGAATTCTCACCATATTCgCTTC
Ftz96_HCR_P1B4: CCTCAACCTACCTCCAACAATGCGACGCAAATCGTCGAACCCATT
Ftz96_HCR_P2B4: CGTGAAGTAGTATGTAAGAGAGAAGATTCTCACCATATTCgCTTC

>WntA_XM_052886643.1_ncbi
ATGGATGACATAAACAAGATCCCTTCCCCCCATAAACAAGTTGAATGCGCTTCCCCGATG
CCGCAGTCTCTGCACGTGCGGCACTCAAGGAATTTAGCAGCACCGAATAGGCCTGTACA
ATCGTCTAACACCTCTGTGGAAACCTTTACAATACTGCACAAAGAAAGCTGCCATAGATT
AGAGTATCTCGTCGAACGACAAAAGCAATTATGTATGCTTTCTGATAAAATGGTACAGGT
GATACAAACAGGAGCGCAACAGGCAATTGATGAATGTCAGCATCAATTTCGGAATAGCC
GTTGGAACTGTAGTACCGTCGACAATTCCACTGATATATTCGGCGGAGTGCTAAAATTTA
AATCTCGCGAGTCTGCATTCGTCCACGCTCTGTCAGCAGCAGCATTGGCTCACACAGTTG
CTCGCGCGTGCAGTCGGGGCGAACTAAACGAGTGTTCCTGTGACGCTCGTGTTAGAAAG
CGAACGCCGCGGCATTGGCAGTGGGGTGGTTGTTCTGAGGATATAAGATATGGAGAAAA
GTTCAGTCGTGACTTTGTAGATGCTAAAGAAGACAAGGATAATGATGAAGGTCTCATGA
ACTTACATAACAATGAAGCTGGCCGCAGAGCAGTCCGCGGCAGGATGCAGCGCGTGTGC
AAATGCCACGGCATGTCGGGCTCGTGCTCCGTGCGCGTGTGCTGGCGCCGCCTGCCGCAG
CTGCGGCTGGTGGGCGACGTGCTGAGCACCAGATACGAGGGCGCCTCTCATGTTAAGGT
TGTAGAGAGGAAGAGAGGCAAGAATATAAGAAAACTGCGACCGCTGCATCCTGATATAA
AGAAACCGAACAAAACCGATCTAGTCTATCTCGAGGACTCTCCCGATTACTGTGAACCG
AACGACGAGTAA

Wnta1_HCR_P1B1: gAggAgggCAgCAAACggAACAACCACCCCACTGCCAATGCCGCG
Wnta1_HCR_P2B1: TTTTCTCCATATCTTATATCCTCAGTAgAAgAgTCTTCCTTTACg
Wnta2_HCR_P1B1: gAggAgggCAgCAAACggAACCTTGTCTTCTTTAGCATCTACAAA
Wnta2_HCR_P2B1: GTAAGTTCATGAGACCTTCATCATTTAgAAgAgTCTTCCTTTACg
Wnta3_HCR_P1B1: gAggAgggCAgCAAACggAACCGCGGACTGCTCTGCGGCCAGCTT
Wnta3_HCR_P2B1: TGGCATTTGCACACGCGCTGCATCCTAgAAgAgTCTTCCTTTACg
Wnta4_HCR_P1B1: gAggAgggCAgCAAACggAAGCACACGCGCACGGAGCACGAGCCC
Wnta4_HCR_P2B1: CAGCCGCAGCTGCGGCAGGCGGCGCTAgAAgAgTCTTCCTTTACg

Wnta5_HCR_P1B1: gAggAgggCAgCAAACggAACGCCCTCGTATCTGGTGCTCAGCAC
Wnta5_HCR_P2B1: TCCTCTCTACAACCTTAACATGAGATAgAAgAgTCTTCCTTTACg
Wnta6_HCR_P1B1: gAggAgggCAgCAAACggAACGGTCGCAGTTTTCTTATATTCTTG
Wnta6_HCR_P2B1: GTTCGGTTTCTTTATATCAGGATGCTAgAAgAgTCTTCCTTTACg
Wnta7_HCR_P1B1: gAggAgggCAgCAAACggAAGGGAGAGTCCTCGAGATAGACTAGA
Wnta7_HCR_P2B1: TTACTCGTCGTTCGGTTCACAGTAATAgAAgAgTCTTCCTTTACg

>Optix_XM_024080404.2_ncbi
ATGCGCGGCTCCTGGGACGAGTCCACGACGGCGGCGCTGCACGCGCGCATCCTGGAGGC
GCACCGCGGGTCCGCCGCGCCCGACCGCGCCGAGCCCGCGTGCGAGCCTCCGCCGCTGA
CGCTGGGCGCGCTGGAGCTGGCGGCGCCCACGCCGCTGCTGCCGCTGCCCACGCTGAGC
TTCAGCGCCGCGCAGGTGGCCACCGTGTGCGAGACGCTGGAGGAGAGCGGCGACGTGGA
GCGCCTGGCGCGCTTCTTGTGGTCGCTGCCCGTGGCGCACCCCAACGTGGCCGAGCTGGA
GCGCTGCGAAGCCGTGCTGCGCGCGCGCGCCGTCGTCGCCTTCCACGCCGGCCGCCACCG
CGAGCTGTACGCCATCCTCGAGCGCCACCGCTTCCAGCGCTCCAGCCACGCCAAGCTGCA
AGCGCTGTGGCTGGAGGCGCACTACCAGGAGGCTGAGCGCCTGCGCGGCCGTCCGCTGG
GCCCCGTCGACAAGTACCGCGTGCGGAAGAAGTTCCCGCTCCCGAGGACGATCTGGGAC
GGCGAGCAGAAGACGCACTGTTTCAAGGAGCGGACGCGATCTCTACTCCGAGAATGGTA
CCTCCAAGATCCCTACCCGAACCCGACGAAGAAGAGGGAATTGGCGGCGGCGACGGGTC
TGACGCCGACGCAAGTCGGCAACTGGTTCAAAAACCGACGGCAAAGAGACCGAGCGGC
CGCCGCCAAGAACCGCTCCGCCGTGCTGGGCAGAGGATAA

Optix1_HCR_P1B2: CCTCgTAAATCCTCATCAAACGCCGTCGTGGACTCGTCCCAG
Optix1_HCR_P2B2: CGCCTCCAGGATGCGCGCGTGCAGCAAATCATCCAgTAAACCgCC
Optix2_HCR_P1B2: CCTCgTAAATCCTCATCAAAGGCTCGGCGCGGTCGGGCGCGGCGG
Optix2_HCR_P2B2: AGCGTCAGCGGCGGAGGCTCGCACGAAATCATCCAgTAAACCgCC
Optix3_HCR_P1B2: CCTCgTAAATCCTCATCAAAGGCAGCAGCGGCGTGGGCGCCGCCA
Optix3_HCR_P2B2: GCGGCGCTGAAGCTCAGCGTGGGCAAAATCATCCAgTAAACCgCC
Optix4_HCR_P1B2: CCTCgTAAATCCTCATCAAACCGCTCTCCTCCAGCGTCTCGCACA
Optix4_HCR_P2B2: AAGAAGCGCGCCAGGCGCTCCACGTAAATCATCCAgTAAACCgCC
Optix5_HCR_P1B2: CCTCgTAAATCCTCATCAAAGCGGTGGCGGCCGGCGTGGAAGGCG
Optix5_HCR_P2B2: GTGGCGCTCGAGGATGGCGTACAGCAAATCATCCAgTAAACCgCC
Optix6_HCR_P1B2: CCTCgTAAATCCTCATCAAAACAGCGCTTGCAGCTTGGCGTGGCT
Optix6_HCR_P2B2: CAGCCTCCTGGTAGTGCGCCTCCAGAAATCATCCAgTAAACCgCC
Optix7_HCR_P1B2: CCTCgTAAATCCTCATCAAAACAGTGCGTCTTCTGCTCGCCGTCC
Optix7_HCR_P2B2: GAGTAGAGATCGCGTCCGCTCCTTGAAATCATCCAgTAAACCgCC
Optix8_HCR_P1B2: CCTCgTAAATCCTCATCAAACGTCGGGTTCGGGTAGGGATCTTGG
Optix8_HCR_P2B2: CGTCGCCGCCGCCAATTCCCTCTTCAAATCATCCAgTAAACCgCC
Optix9_HCR_P1B2: CCTCgTAAATCCTCATCAAATTTGAACCAGTTGCCGACTTGCGTC
Optix9_HCR_P2B2: GGCCGCTCGGTCTCTTTGCCGTCGGAAATCATCCAgTAAACCgCC

>Wnt1_XM_024099417.1_**sense**_ncbi
CAGCAGCTGGATTTTGTCAGTCCAGCTAGGAAGGGGGGCATAGCAAAGGCAGGCGAACC
AAATAACTTATCACCCTTGTCTCCAAGTGTCCTATACATGGACCCGGCTGTTCACGCCAC
CTTGAGGAGGAAACAGAGAAGGCTAGCGAGGGAGAACCCTGGGGTCCTCGCAGCAATA
TCCAAGGGAGCCAGCATGGCTGTGGCCGAATGCCAGCATCAGTTCAAATACAGGAGATG
GAACTGTTCTACAAGAAATTTTTTGCGAGGGAAGAATCTATTTGGAAAAATTGTTGACAG
AGTTTCGCCGGACAAAGCCCCCCCGGCCGGGGCGGCTATAATTACTAATATACACGTCG
ACACGCCATTGACGATTGACGCGACATCTTCATTTCATTGTGGTGTAAACCTCAAGGATC
GCATTAACACGGACGATGACAACGGCACAATA

Wnt11_sense_P1B2 CCTCgTAAATCCTCATCAAAGCTGGATTTTGTCAGTCCAGCTAGG
Wnt11_sense_P2B2 GGGGGGCATAGCAAAGGCAGGCGAAAAATCATCCAgTAAACCgCC

Wnt12_sense_P1B2	CCTCgTAAATCCTCATCAAAACCCTTGTCTCCAAGTGTCCTATAC
Wnt12_sense_P2B2	GGACCCGGCTGTTCACGCCACCTTGAAATCATCCAgTAAACCgCC
Wnt13_sense_P1B2	CCTCgTAAATCCTCATCAAAAAGGCTAGCGAGGGAGAACCCTGGG
Wnt13_sense_P2B2	CCTCGCAGCAATATCCAAGGGAGCCAAATCATCCAgTAAACCgCC
Wnt14_sense_P1B2	CCTCgTAAATCCTCATCAAACCGAATGCCAGCATCAGTTCAAATA
Wnt14_sense_P2B2	GGAGATGGAACTGTTCTACAAGAAAAAATCATCCAgTAAACCgCC
Wnt15_sense_P1B2	CCTCgTAAATCCTCATCAAAGAATCTATTTGGAAAAATTGTTGAC
Wnt15_sense_P2B2	AGTTTCGCCGGACAAAGCCCCCCCGAAATCATCCAgTAAACCgCC
Wnt16_sense_P1B2	CCTCgTAAATCCTCATCAAAATTACTAATATACACGTCGACACGC
Wnt16_sense_P2B2	TTGACGATTGACGCGACATCTTCATAAATCATCCAgTAAACCgCC

>spalt_XM_052891247.1_ncbi
ATGCCGCGCGTCAAGCCCGCCTGCGTCCGCCGCGTCTCCATCGGTGAAAGCTCGGGATCT
TGTTCGGAGGAAGATGTTGGCAATGCCATGCCGGATGAAGCGAGAGATAGGCCAGAGGC
GCACATGTGTCCACGCTGTCAGGAACAGTTCGAAAACCTTCACGATTTCTTGTATCATAA
GCGACTTTGCGATGAGAAAGCAATGCAAATGGGTGAAGAGAGGATGCACTCCGATCCAG
AGGATATGGTAGTGTCGGGGGATGAAGAGATGGATGGTCCCAATAAACGACTAGAACAA
GTCAGGAGGCATCGACAAGATGCTGAAAATAATAATAGTCTCGAAGACGGCGAGGCCGA
AATACCTGAAGCCGACATGCCCCCCGTGGGCTGCCGTTCCCTTTGGCAGGACACGTTACT
CTTGAGGCTCTACAAAATACGAGAGTAGCGGTCGCCCAATTCGCTGCAACAGCGATGGC
AAATAATGCGAATAACGAAGCTGCTATACAAGAATTACAAGTGTTACACAACACTCTAT
ACACTTTACAGTCACAACAAGTATTTCAACTTCAGTTAATACGTCAGCTTCAGAATCAGT
TATCTCTAACTCGACGGAAAGAAGACGATCCACACAGCCCACCGCCAAGTGAACCAGAA
CAGAATGCCCCGTCAACGCCGGCTCGATCACCGTCGCCGCCGCGTCCGCCACGGGAGCC
GTCGCCTGTTATACCCTCTCCTCCTACTAGCCAAAGTTTGCCGTCGACTCACACACATCAC
ACACCCAAAACTGAACAGATATCTATCCCTAAGATTCCAACTTCCTCACCATCTTTAATG
ACCCACCCACTTTATAGTTCAATTTCTTCGTCATTAGCATCTTCCATCATAACAAACAATG
ATCCTCCACCGTCCCTAAATGAACCAAACACACTTGAAATGCTTCAAAAACGGGCACAG
GAAGTACTCGACAATGCATCACAGGGCCTTCTAGCAAACAATCTTGCCGACGAATTAGCT
TTTCGAAAATCCGGAAAAATGTCACCTTATGATGGAAAAAGTGGTGGCCGTAACGAACC
TTTCTTTAAACATCGCTGTAGATATTGTGGAAAAGTGTTCGGTAGCGACTCTGCACTTCA
AATTCACATTCGTTCTCACACAGGGGAAAGACCTTTCAAATGTAACGTCTGTGGCTCTCG
ATTTACAACCAAAGGAAATCTTAAAGTTCATTTCCAAAGGCATACTTCGAAATTTCCACA
TGTCAAAATGAACCCTAATCCCGTTCCAGAACATTTGGATAAATATCACCCACCGTTATT
AGCGCAATTGTCGCCGGGGCCCATTCCTGGAATGCCGCCACATCCACTTCAGTTTCCCCC
AGGAGCCCCAGCTCCCTTTCCGCCAAACTTGCCATTATACAGGCCACCGCATCACGATTT
ATTGCCTCCACGCCCTCTGGGTGATAAGCCTCTCTCACATCACCCACTTTTTGCTATGCGA
GAAGAACAAGACGCACCAGCTGATCTCAGTAAACCTTCCGCACCAAGCCCTCCTCGACC
CGCGTCTGATATTTTTAAGTCTGAACCTCAAGACGAAGAGAGTCAACGAGATTCCAGTTT
TGAAGAGACTGATCGTATATCACCTAAGCGAGAAATCGAAGACAATGATATAGGACAAG
ATGCAGAACAAGATCGATACCCATCCACATCACCGTACGATGACTGCAGTATGGATTCC
AAATACAGCAATGAAGATCAAATCGGCAGAGATAGTCCACACGTGAAGCCCGATCCAGA
TCAACCGGAAAATCTCTCAAGTTCGGAGAGCGGGCGGAGTGCACGGGGGTCGCCACCGT
CGCCGTCGCCGTCGCCGTCGGCGCTGTCCACGCCGCCGCGTCTGCCGCACCACTCGCCGC
TGCCGTCGCCCCCGACGCCCCTGGCGGCGCTCGGCGCGCTCGGCGGATCGCCCTTCAGCC
CGCTCGGACTTGCCTTTCCTCCCGCAGTGCGCGGCAACACAACGTGTACCATCTGCTACA
AGACATTCGCCTGCAACTCGGCACTGGAGATCCACTATCGAAGCCACACCAAGGAACGG
CCATTCAAGTGCACCGTCTGCGATAGAGGCTTTTCTACCAAGAGCAGTGGCGGCGGTTGT
CAGTGCGGAAGGCGTGCGCGCGCACCCCGCCCGCCGCACGCCACTGCTTTGGACCTCTG
GAACGCCTTCGTCTACCCGGGCAACATGAAGCAGCACATGCTAACGCACAAGATCAGAG
ACATGCCGCCTGGTTTTGACAAGGGGCCGGGAGGACCTTCCGGACCCCCAAGCGAGGAA
GGGCGGGACCCCAGCCCGGACAGACGGTCGTCCCCAGAAAAGCTGGATCTGAAAAGATC
ACCCCCGGTGCATCCTCCACCGCCAATGTCACACCCACCTATTGACATGCCACCTCTACC
AAAAAGACCTACAGTGCCCAGTATCCCGAGTCACCCCCCACCGTCGCGTCGTCGAAGCA
CCTGTGCGGCGTGTGTCGCAAGAACTTCTCCTCATCATCAGCGCTGCAGATACACATGCG

CACGCATACCGGAGACAAACCCTTCCGATGTGCTGTCTGTCAGAAGGCGTTTACCACCAA
AGGCAATCTTAAGGTGCACATGGGCACGCACATGTGGAGCGGCGGCGCGTCGCGGCGCG
GGCGGCGCATGTCGCTGGAGCTCCCGCCGCGGCCGCTGCACGAGCCGCACGAGCTGCTG
CGGCGCCCCGACCTCTTCTACCCCTACCTGCCGGCGCCTTTCCTCAACGGCATGCAACAG
AAGCTGAACGAGATATCTGTAATACAGCAGAACGCCGGACAAAACGGCGTAGCTGGAA
AATTCCCCGGTCTGCTCGGCTTCGGAGCGTTCGGGGCCGGGAGACCGGGCGCCGCGTCCC
CGCTCGAGAGGCCTCCCTCGCTGGAGGGGGGAGACGAGCGACAGGCGGCGATGCGTGA
GCTGGCCGAGAGGGGACGGGAGCTGGCGGAGAGGAGTCGGCAGATGCGCGAGGAGAGC
GAGCGGGAGCACTACAGGGCCGCGGGCGGACTGCCCGCGCACGCGCACGCGCCCAACCC
CGCGCAGGCCTCGCCGCCCGCGCCGCACGCGCACCCGCACCCCCTCGCGTCGCTGCCGCC
GCCCGCGCGGACAGAAGGCCTCACCGTATAA

spalt1_HCR_P1B1: gAggAgggCAgCAAACggAAATCCGGCATGGCATTGCCAACATCT
spalt1_HCR_P2B1: GTGCGCCTCTGGCCTATCTCTCGCTTAgAAgAgTCTTCCTTTACg
spalt2_HCR_P1B1: gAggAgggCAgCAAACggAAGTGCATCCTCTCTTCACCCATTTGC
spalt2_HCR_P2B1: CGACACTACCATATCCTCTGGATCGTAgAAgAgTCTTCCTTTACg
spalt3_HCR_P1B1: gAggAgggCAgCAAACggAAATTTCGGCCTCGCCGTCTTCGAGAC
spalt3_HCR_P2B1: CCCACGGGGGGCATGTCGGCTTCAGTAgAAgAgTCTTCCTTTACg
spalt4_HCR_P1B1: gAggAgggCAgCAAACggAACGTTATTCGCATTATTTGCCATCGC
spalt4_HCR_P2B1: ACACTTGTAATTCTTGTATAGCAGCTAgAAgAgTCTTCCTTTACg
spalt5_HCR_P1B1: gAggAgggCAgCAAACggAAGTGTGGATCGTCTTCTTTCCGTCGA
spalt5_HCR_P2B1: CTGTTCTGGTTCACTTGGCGGTGGGTAgAAgAgTCTTCCTTTACg
spalt6_HCR_P1B1: gAggAgggCAgCAAACggAAGAGTCGACGGCAAACTTTGGCTAGT
spalt6_HCR_P2B1: GTTCAGTTTTGGGTGTGTGATGTGTTAgAAgAgTCTTCCTTTACg
spalt7_HCR_P1B1: gAggAgggCAgCAAACggAAAGGATCATTGTTTGTTATGATGGAA
spalt7_HCR_P2B1: TGTGTTTGGTTCATTTAGGGACGGTTAgAAgAgTCTTCCTTTACg
spalt8_HCR_P1B1: gAggAgggCAgCAAACggAAATTTTTCCGGATTTTCGAAAAGCTA
spalt8_HCR_P2B1: CCACCACTTTTTCCATCATAAGGTGTAgAAgAgTCTTCCTTTACg

Sequences used for Frizzled phylogenetic analysis

>frizzled2_Drosophila_melanogaster_FBgn0016797_flybase
ATGAGACACAATCGACTGAAGGTCCTGATCCTGGGACTCGTCCTCCTGCTGACATCTTGT
CGAGCGGATGGACCGCTGCACAGTGCGGATCACGGCATGGGCGGAATGGGCATGGGTGG
TCACGGCCTGGACGCGAGTCCCGCACCCGGTTACGGAGTGCCAGTCATACCCAAGGATC
CCAATCTGCGATGCGAGGAGATCACCATACCAATGTGTCGGGGCATTGGCTACAACATG
ACATCCTTCCCCAACGAAATGAACCATGAGACCCAGGACGAAGCGGGCCTGGAGGTGCA
CCAGTTCTGGCCCCTGGTGGAGATCAAATGCTCGCCGGACCTCAAGTTCTTCCTGTGCAG
CATGTACACGCCCATCTGCCTGGAGGATTACCACAAGCCGCTGCCCGTTTGCCGGAGTGT
CTGCGAGAGAGCCCGCTCGGGATGCGCACCCATCATGCAGCAGTACAGCTTCGAATGGC
CGGAGAGAATGGCGTGCGAGCACTTGCCTCTTCATGGTGACCCCGACAATCTGTGCATGG
AACAGCCCTCGTACACGGAGGCTGGCAGCGGTGGCAGCTCGGGCGGATCGGGTGGCTCT
GGCAGCGGTTCCGGCTCCGGCGGCAAACGGAAGCAAGGAGGCAGTGGCTCGGGCGGCA
GTGGGGCCGGCGGCAGCAGCGGTTCCACCTCAACGAAGCCGTGCCGCGGACGCAATTCA
AAAAACTGCCAAAATCCCCAAGGAGAAAAGGCAAGCGGAAAAGAGTGCAGCTGCTCGT
GCCGCTCCCCACTCATCTTCCTGGGGAAGGAGCAGCTGCTGCAGCAGCAGTCGCAGATG
CCCATGATGCACCATCCACACCACTGGTACATGAACCTCACTGTCCAAAGGATCGCCGGC
GTTCCAAACTGCGGCATACCGTGCAAGGGGCCCTTCTTCAGCAACGACGAAAAGGATTT
CGCCGGCCTCTGGATCGCCCTGTGGTCGGGACTGTGCTTCTGCAGCACGCTCATGACCCT
AACCACATTCATCATCGACACCGAAAGGTTTAAGTACCCGGAGCGGCCCATTGTCTTCCT
CTCCGCCTGCTACTTCATGGTGGCAGTGGGCTACCTGTCGCGCAACTTCCTGCAGAACGA
GGAGATCGCCTGCGACGGCCTGCTGCTCCGGGAAAGCTCCACGGGTCCGCACTCGTGCA
CCCTGGTCTTCCTGCTTACCTACTTCTTTGGCATGGCCTCGTCCATCTGGTGGGTGATCCT

CAGTTTCACCTGGTTCCTGGCCGCTGGTCTGAAGTGGGGCAATGAGGCCATCACCAAGCA
CTCGCAGTACTTCCACCTGGCCGCCTGGTTGATTCCCACTGTCCAGTCCGTGGCCGTACTC
CTGCTCTCGGCGGTGGATGGCGATCCCATTCTGGGCATCTGCTATGTGGGCAACCTCAAT
CCGGATCACCTAAAGACCTTTGTGCTGGCCCCGCTCTTCGTTTACCTCGTAATCGGCACC
ACCTTCCTGATGGCCGGCTTTGTGTCCCTCTTCCGCATCCGCTCGGTTATCAAGCAACAGG
GCGGTGTAGGAGCTGGTGTCAAGGCGGACAAGCTGGAGAAACTGATGATCAGGATTGGC
ATCTTCTCGGTGCTCTACACGGTGCCGGCCACCATAGTTATCGGATGTTACCTGTACGAA
GCAGCCTACTTTGAGGACTGGATCAAGGCCCTGGCCTGTCCATGCGCCCAGGTGAAGGG
TCCCGGCAAGAAGCCTCTCTACTCGGTCCTGATGCTCAAGTACTTCATGGCCCTGGCCGT
GGGCATCACCTCGGGCGTGTGGATCTGGTCTGGCAAGACGCTGGAGAGCTGGCGACGCT
TCTGGCGGAGACTCCTAGGAGCGCCGGACCGCACGGGCGCCAACCAGGCGCTGATCAAG
CAGCGGCCTCCGATCCCGCATCCCTATGCCGGATCTGGAATGGGAATGCCCGTGGGCTCG
GCGGCGGGCTCCCTGCTGGCCACGCCCTACACCCAGGCGGGCGGAGCCTCGGTGGCCTC
CACCAGCCACCACCACCTGCACCACCACGTTCTCAAGCAGCCGGCGGCCAGCCACGTAT
GACATGGAGAGTCGGGGGGAGCATCGACCATGGGCGGCGGTGGGGGCGGCGGTAGCAC
CCTTGGCGGCGGCACCCTGGGCCACGGCACCGCGATGAGCAGCAGCACGGTCGGCATGG
GCCCTCTCCCCGGCACCCTGATGCATGGCGCCTCCGGCGGAGGCGTGGGCGGCGGCAAT
CCCGGCAACGTTTACGGCAATGGCAACAACGGAGTGGGCGGACAGAATACCGCTCCCGG
CTCCGTGATCGACTCGCGGGCCGTGTCCGTGGTGGTCACGCTGCCCGGCGGCCCGGGTGC
CCAGCATCCTGGCCAGGCGCTCAGCGACTATGGTCCCATATAG

>frizzled_Drosophila_melanogaster_FBgn0001085_flybase
ATGTGGCGTCAAATCCTGTTTATTTTACCCACCCTGATACAGGGGGTCCAGCGCTACGAT
CAGAGCCCCCTCGATGCGAGTCCGTATTATCGCAGCGGCGGGGGATTAATGGCCAGTTCT
GGGACCGAACTAGATGGACTGCCACATCACAATCGCTGTGAACCCATCACCATATCGAT
CTGCAAGAATATACCATATAACATGACCATTATGCCAAATCTTATTGGCCATACCAAGCA
GGAGGAGGCGGGTCTGGAGGTCCATCAGTTTGCTCCGCTCGTGAAGATCGGCTGCAGTG
ATGACCTCCAGTTGTTCCTCTGTTCCCTGTACGTTCCGGTCTGCACAATTTTGGAGCGACC
CATTCCGCCTTGTCGATCTCTGTGCGAATCGGCGCGGGTATGTGAAAAGTTAATGAAAAC
CTACAACTTTAACTGGCCGGAAAATCTGGAGTGCTCCAAATTTCCCGTCCATGGAGGCGA
GGATTTGTGCGTGGCGGAGAATACCACATCATCGGCCTCCACGGCGGCCACGCCCACAA
GGAGTGTGGCTAAGGTCACTACCCGTAAACACCAGACGGGCGTAGAAAGTCCGCACCGA
AACATTGGATTCGTGTGCCCCGTGCAACTGAAAACGCCTCTGGGAATGGGCTACGAACT
AAAAGTTGGCGGAAAGGATCTGCATGACTGTGGAGCCCCATGTCATGCCATGTTCTTCCC
GGAGAGAGAAAGGACTGTGCTTCGATACTGGGTTGGATCCTGGGCAGCGGTCTGTGTAG
CCAGCTGCTTGTTTACGGTGCTCACCTTCTTGATTGACTCGTCGCGTTTTCGCTACCCGGA
GAGGGCCATTGTCTTCTTGGCCGTTTGCTACTTGGTAGTTGGATGTGCCTACGTGGCGGG
ACTGGGAGCGGGCGACTCTGTGTCGTGCCGCGAACCATTTCCGCCGCCCGTCAAACTCGG
CCGCCTGCAGATGATGTCCACCATCACCCAGGGCCACCGACAAACCACGTCCTGCACGG
TTTTATTCATGGCACTCTACTTCTGCTGCATGGCGGCCTTCGCGTGGTGGTCGTGTCTGGC
ATTCGCCTGGTTTTTGGCCGCTGGCCTCAAATGGGGCCACGAGGCGATTGAGAACAAGTC
GCACTTATTCCACCTGGTTGCCTGGGCGGTGCCCGCCCTTCAGACCATCTCCGTTCTGGCC
CTGGCTAAAGTTGAAGGTGACATCCTTTCTGGCGTTTGTTTCGTGGGCCAGCTGGACACG
CACTCCCTGGGCGCGTTCCTGATCCTTCCACTCTGCATTTATCTCTCGATCGGAGCACTAT
TCCTGCTGGCCGGATTTATTTCGCTTTTCCGGATCCGGACAGTTATGAAAACGGACGGAA
AGAGGACAGACAAACTGGAGCGCCTGATGTTGCGAATAGGTTTCTTCTCTGGACTTTTCA
TTCTGCCCGCCGTGGGATTACTGGGCTGCTTGTTCTACGAGTACTACAACTTTGACGAGT
GGATGATCCAATGGCACAGGGATATCTGCAAGCCCTTCTCAATTCCGTGCCCGGCAGCCA
GGGCGCCGGGATCTCCAGAAGCCCGCCCCATCTTTCAGATCTTTATGGTCAAGTACCTTT
GCTCCATGCTGGTGGGGGTCACTTCCAGCGTTTGGCTGTATTCCAGCAAGACGATGGTCA
GCTGGCGGAACTTCGTGGAGAGGTTGCAGGGCAAGGAGCCCCGGACCCGGGCGCAGGC
GTACGTCTAGTATGAGACGGGTCCGGCGGGCGGGGCCAAGTCCACGCCCCTTACTCCCG
ATCCGGAGGCGGCTAACGAAAGTTACTTAGAGTTTAGCACATAG

>frizzled9_like_Bicyclus_anynana_XM_024087045.1_ncbi

ATGATGGGGAGGATTTTGTGTGTTTTTCTCCTGGTCTGGATGGTCGCTGCTGGCCAGGAG
GAGGAGGGTGGGAAATGCGAGAGGATCACCCTGTCCCAGTGCCAGGATCTGGGGTACAA
TTGGACCGCTATGCCCAATCTCATTGGACATAGAGACCAGAAGGAGGCAGAAGAAGCGA
TGACTGCGTTCACGAGCATCCTTGCGAGCGAATGCTCGGTGCACGCTCGCTTCCTGCTGT
GCTCGGCCTTCGCCCCGCTGTGCTCCGAGCAGGTGTCGGGCTCCGTCAGCGCCTGCCGCG
CGCTCTGCGACAAGGTCGTCCGCGACTGCAAGAACCAGATCGCGGCCCTGCCCGACGGC
ATCAAGCTCGACTGCTCCGCCTTCCCGCTCCGCCCGGACTGGCGGCTCTGCATGCGGCCG
CCCAACGCCAGCGAGGAGCCCGAGCCGCCGCCGGTGCCGCGCTGGCCCTTCAACGAGCA
AGATCTGAAAGAGCACGCGTGTCCCCCGGGCTACGCGCACTCCCCCACGGGCTCGTGCT
GGCCCGCCTGCGACAAGCCCGCGCGCTACACGCAAGCTAACAAGAGAAAAGCGGAGAT
ATGGATGCTCACGCTCGCCTGGTTCTCTCTGCTCTCCACTTCTTTCGCGCTGCTCACGTTC
TGCGCTGAACCGTCGCGATACCGCTACCCCGAGAGGCCCGTCGTCTGGATGGCCGCGTGC
CACGCTGTCGTCGCGCTCGCCTACGTCACGAGAGGCTGGCTCGGTGCGAAGACCGTCTCC
TGCACTGGAAACCTGTTGGCCGTCGACGGAATGGGTTCGACGATTTGCGTCGCATTCTTC
TCTCTTACATACTACTTCACGTTAGCGGCGGACGCGTGGTTCGCGAACGCGTGCGTAGCG
TGGTACTTGACCGCTGCGAGCGAGTGGTCCACCGAGGCGTTGGAGCGGGCGGCGGCGTA
CCTGCACGCGGTGGCGTGGGGCTGGGCGGGGGCGTGGACGGCGGCGGCGCTGGCGCTGC
GGCGGGTGACGGCGGACGAGCTGACCGGGACCTGCGGCGTGTCGGACGAAGCGGCCGC
GGCGCTGATCGGCGTCCCGCGAGGCGCTCTGCTCATCACCTCAATAGCGTTGGCTATCGG
CGCGTGTCCGGGCATAATGAGAGTGCGCCGCGCGTTGGACTCGCGAGGAGCGAAACGAG
TGGGACGGTTGGCCGCTCGAGCGGCCGCTGGAGGCCTGCTCTACCTGTTCTTAGCGGCGT
GCGCGACCGGCGCGCGCGTGATTGAAGCCAGAAACAGAGCGGCGCAAAAGAGCTTGCC
GCTTCTGGCGGCGATGGGCGCGGGCAGTCTGGATGACTCGGCGGGGTCGCGCGCCGTGG
CCGTGAGCCTGTGGCTGTGCTGCGGGGTCGCCGCGGGGGCGTGGTCGTGGTCGCGCAAG
TCGGCGGTGGTGTGGCGCAAGGCGCTGTGTCCGCCGCGCAAGGCCCCGTGCTGCGCGCC
GCCGCTGCTCGGACCCCATCACCCGTACTATAAAAGACCTCTGCACGTGTCTAGAGTGTG
A

>frizzled_Bicyclus_anynana_XM_024099445.1_ncbi
ATGGTCTGGCGATGGATTTTGTTTGTATACGCCTTGTCTGTTATAACTAAAAGTTCACGTG
TTACAGTGAACCAAGGGGATACTTTACCTCATCATGGACGATGTGAACCAATAACAATA
CAGTTTTGCCAGCAGATACAGTACAATCAAACAATTTTTCCAAACTTGCTGAACCACGCT
AAACAGGAAGATGCTGGATCCGAAGTACATCAATATACACCTCTTATAAAAGTAAACTG
TTCGCCAGACTTGAAATTCTTTTTGTGCTCAGTTTTTGCACCAGTTTGTACAATATTAAAT
GAACCTATTCCGCCTTGTCGTCATTTATGCGAATCTGCAAGACACAACTGCGATGAGATA
CTTATACATTTTGGATTTGAGTGGCCAAAGGCGTTAGAGTGTTCACGATTTCCAGTGGTT
ACAGATGAAAATGTTATATGTGTTGGGGATAACAATGTGACTCATGAACCTCGTAAGGCT
ACAGAGACCAAACTTCGGAAGGTAGATTACAAGTCAAACAATGATAGAGACCCTACAAA
GTTACAAGGTGCTAAAGACCTTGGTTTTGTTTGTCCAGTACATTTTAAAATACCTAAGAA
TCTGGATTTAGAGTATTCCCTGAAAGTTGGAGATAGAACTGAACGTGACTGTGGGGCACC
ATGCAACGGAATGTTTTTCAGTGAAGAGGAAAAAAGTTTTGCACGATCATGGATTGTTAT
TTGGGCCACTTTGTGCTCAATCAGTTGCTTGTTCACTGTTTTGACATTTGTAATAGATACA
GATAGATTCCGCTATCCAGAGAGGCCTATAATTTTTTTATCAGTGTGTTATCTAATGGTTG
CCGCAGCCTATGTTATGGGATGGGGTGCAGGAGACTCTGTCAGCTGCCAAGGGCCCTTTC
CGTCAACTATTAGTGGAACAAGGTTGCCTAACATATCAGTTATTACACAAGGAACAAAA
CATGAGCCGTGTACAATCCTTTTTATGATTGTATATTTCTTCAGTATGGCATCTAGCATTT
GGTGGGTTATTTTAACACTCACATGGTTTTTAGCAGCAGGATTGAAGTGGGGGCATGAAG
CAATAGAAGCTAATTCACAATATTTTCATCTAGTGGCTTGGGCAGTGCCTGCCATTAAAA
CAATATTTATTCTAGCTATGGGCAAAGTTGATGGTGATGTACTCTCTGGTGTGTGCTATGT
TGGGCTTTGGGATGCAGAAACTTTACGTGGTTTTGTATTGGCACCATTATGTGTTTATTTA
GTCTTGGGCACTATATTTTTAATGGCAGGTTTTGTGTCACTATTCAGAATAAGAACAGTT
ATGAAACATGATGGTACCAAAACTGATAAATTGGAAAAGTTAATGATACGTATTGGTAT
ATTTGGTGTGTTATACACTGTTCCTGCTCTTATTGTGATTTCATGTTTATTTTACGAACAG
GCTTATTTTGACAAATGGATGGTGACATGGCACCGTGACATGTGCTCAACACCTCTCTAC
TCTATTCCATGCCCATTTACACATCAGGAGATGGAAAGGCCTAAATTTGAAGTGTTCATG

ATAAAATATCTTATGACAATGATTGTTGGCATCACATCAAGTTTTTGGATTTGGTCTGGTA
AAACTCTAGTTTCATGGCATCAATTTTTTGATAAAATAAGGGGAAGACGAGTTGAAGCAT
ATGTTTGA

>frizzled4_Bicyclus_anynana_XM_024081478.1_ncbi
ATGAAGTGTTTTGTGGTATTAATAACTGTGACCCTCGTCTACGAGATCGTAGCCGAGGCG
TCCGTGAGGACATGTGAACCGATCAAGGTGGCTATGTGCAAGAACATCGGATACAACCA
GACGGGAATGCCCAACTTGGCTCGGCACACTCTCCAAGCGGACGCCGACGTCACACTAC
AGACCTTCAGCCCCCTGGTGCAGTATGGATGCTCGTCCCAGTTGCATTTGTTTTTGTGTGC
GGTGTACGTGCCCATGTGCACTGATAAGGTCGCGTTACCGATAGGTCCGTGTAGGGGTTT
GTGCGAGAGTGTTTACGCCAGATGTTACCCCGTGTTGCGGGGTTTCGGGTTCCCTTGGCC
GGCGGAGTTGGACTGTTCTCTATTCCCGGCGGAGAATAACCATGAACACATGTGTATGGA
GGGTCCTGGGGAGCGCGCGCCGCCGATCGGTACGATACCGTTGGACACGACGAGGGAAG
GACGAGGGACTTGCAGACGTTTGGTGAAGCCGAACAGTTGGGTGTGGGTTAGGGGGTCG
GGGCGGTGCGCGCAGTTCTGTGACGCTGAAGTGTTGTGGGAGGTGGGGGAGAGGCGGGC
GGCTGAGGTGTGGCTGGCGACTTGGGCAGCGTTGAGCTTCGCGTGTACTCTTGCGGCGGT
GGCGGCGCAGCTGGCGTGTGGAGCCAGGGGCGGAGCTGGCGAGCGCGCGTTGGTGCTGG
TGGCACTGTGCCGGTGCGCGGCGGCGGCGGGCTGGGGCGTGCGCGCGGCTGCGGGCCGG
ACGGCGGCGGGCTGCGCCAAGGACTCCACGTCTCCCACCAGGATGCTGTTAGCGCATGA
CGGCTTAGCGAACCCCAACTGCGCGGTCGTCTTCTTGCTGCTGTATTACTTCGGGCTGGCT
GCGTCAGTTTGGTGGGTGGTAGTGACGGGTGCGTGGCGAGCGAGCGTGCTGCGCCCCCC
AGCCAGTAGCGGCGCCAGGAACGACCGCCACTCCTCCCTGCTGCAGCTGGCTGCGTGGG
GCGTGCCCGCCGCGCTGGCCGCCGCCGTGCTGGTCACAAGGGACGTGGATGCTGATGAG
CTTACAGGCACATGCTTCGTGGGCAATCAGTCTAGCAAATCTTTGCTAGCGTTGGTTATC
GTTCCCGAAGCGATATGCTTGCTTCTGGGCAGCGTGTTCCTCGCTTCCGGCCTTCGCGCA
GTGCTTCGTAAACCTGTACCGATTCCAGCTCCGGCAACGCTTCTGAATTCTGCACCGCAA
GCGCACCCTGATCAGAGTCTGCTGAGGCTAGGAGCCTTCGCTGCGTTGTACGCTGTGCCA
TCTGCCTGCATACTGGCGACATGGGTGTATGAATATATTCTGAGAGAAAATTGGTTGGCT
GCCCCCGTCCCTTCGACGGAACCTTCGACGCAACCGCGTCCAGCATTCTGGGTGTTCCTT
TTTAGGATATTCGCATCACAAATACTGGGTGTCATGGTGGCGGTTTGGATAGCCACGCCA
CGGTTGAAGGCGCTGTGGAGGAGAATCAGTGGGCCAAGAAAACCAGCGTTAGCAAAAT
GCCCACCTGGTCCGACGCCTACACCGTTAACATTGCATTGCTACGCTACGCATCCGCATA
CGTTGACGAGACACCCACAGAAATACGCCACATACCGACCACCACAGCAACAGTCGTAT
AGAAAACCTCGACATTATCATTATTCTGCTGGAGAAACTATCCTATGA

>frizzled2_Bicyclus_anynana_XM_024097661.1_ncbi
ATGAAAAAAGAAAAACCGACGTTTATGAGGCTGTTGTGTACAATTTTGAATCCGCGGCTC
GTGGCGCTGCGCGCGCGCAACGCCAGCGCGCCCGGCGTGCCGGCGCCCGCCTGCGGCCT
GCCGTGCCGCGGCGCCTTCTTCTCGCGCGAGGAGAAGGAGTTCGCCGCCGTGTGGGTCGC
GCTGTGGGCCGGCCTGTGCGCCGCCTCCACGCTCATGACGCTCACCACCTTCGCCATCGA
CTCGCAGCGCTTCAAGTACCCCGAGCGGCCCATCGTGTACCTGTCGGCCTGCTACTTCAT
GGTGGCGCTGGGCTACCTGGCGCGCCTGGCGCTGGGCCACGAGGCCGTGGCGTGTGACG
GGCCGCTGCTGCGGACGTCGGCGGGCGGGCCCGGCGCCTGCACGCTGGTGTTCGTGCTG
GTGTACTTCTTCGGCATGGCGTCGTCCATCTGGTGGGTGGTGCTGTCGTTCGCGTGGTTCC
TGGCCGCCGGGCTCAAGTGGGGCAACGAGGCCATCGCCGGGCACGCGCAGTACTACCAC
CTGGCGGCGTGGCTGGTGCCGGCCGCCAAGACCGTGGCCGTGCTGCTGGCGGGCGCCGT
GGACGGCGACCCCGTGGCCGGCGTGTGCTACGTGGGCAACTCGTCGCCCGAGCACCTGC
GGCGCTACGTGCTGGCGCCGCTCGTGGTGTACTTCGCGCTGGGCGCCTCCTTCCTGCTGG
CCGGCTTCGTGTCGCTGTTCCGCATCCGCAGCGTCATCAAGCGGCAGGGCGGCGCGGGC
GCGGGCTCCAAGGCCGACAAGCTGGAGAAGCTCATGATCCGCATCGGCGTGTTCAGCGT
GCTGTACGCCGTGCCGGCCGGCGTGGTGATCGGCTGCCTGGCGTACGAGGCGGGCGGGC
GCGAGCGCTGGCTGCGGCGCGTGGCGTGCGGCGCGGCGTGCGGCCCGCGCCCCGTCTAC
TCGGCGCTCATGCTCAAGTACTTCATGGCGCTGGCCGTGGGCATCACGTCGGGCGTGTGG
ATCTGGTCGGGCAAGACGCTGGAGTCGTGGCGCCGCGTGTGGCGCGGGGGGCGCGCGCC
GCCGCCCGCGCAGCGCGCGCTG

>frizzled7B_Papilio_machaon_XM_014502879.2_ncbi
ATGGGGAGTGTTTACGTGCCGTTTGTTCTTCCAGTGCTTTATTTGTTTGTTTTAACTGAAA
CTTCACGTGTTACTGTTTATCAAGGGGATTCTTTGCCGCACCATGGTCGATGTGAACCAA
TTACTATACCACTTTGCCAAAATATCAGATACAACCAAACAATTTTTCCTAATATACTAA
ACCATGCCAAACAGTCCGATGCAAGTCAAGATGTGTTAAAATTTACAGTGTTCATAAAA
GTTAATTGTTCTCCGGACTTGAAGTTTTTTCTGTGTGCAGTATATGCCCCTTTATGTACAA
TCTTAGAGAACCCAATACCGCCGTGCCGTCATTTATGTGAATCTGCTAAACACGGTTGTC
ACACTAGTATGTTTACTTACGGATTTCATTGGCCAGAAAATTTGAATTGCTCATCATTTCC
AGTAGCTGGGAAAGGTGTCTTATGTTTTGGCGAAAACAATGGTACGCAAGAAACCCGAC
AACGTGCGAAGACAAAACTTTCGAAGGTAGATTATCAGGCAAACATTGAACGAAATCCT
ACAAAACTGCATGGGGCAAGGGACATCGGATTTGTATGCCCCATTCAGTTCAAGATACCT
AAAAATTTGGACTTAGAGTACTCTTTGAAAGTTGGAGATAAAATGGAGAAAGACTGTGG
TGCCCCTTGCAATGGTATATTTTTCAGTCAAGATGAAAAGAATTTTGCAAGACTTTGGAT
TGGAATATGGGCAACATTGTGCACCATAAGTTGCCTATTTACTGTTTTAACTTTTCTAATA
GACACAGATCGTTTCCGTTATCCAGAAAGACCTATTATATTTCTCTCAGTATGCTATCTGA
TGGTTGCAGCATCATATGTAATGGGATGGGGAGCAGGTGACAGTGTAAGTTGTCAAGGA
CCATTTCCACCAACTATTAGTGGTACAAGATTACCAAACATATCTGTAATAACACAAGGA
ACCAAACATGAACCATGTACCATTCTTTTTATGGTTGTGTATTTCTTCAGCATGGCCTCAA
GTATTTGGTGGGTTATATTGACACTCACTTGGTTCTTAGCAGCAGGGTTAAAATGGGGTC
ATGAAGCAATTGAAGCAAACTCTCAATACTTTCATTTGGCTGCTTGGGCTGTGCCAGCCA
TAAAAACAATATCTATATTGGCAATGGGAAAAGTAGATGGTGATATATTATCGGGTGTCT
GCTATGTGGGACTTTGGGATGCAGATGCTCTACAAGGATTTGTTTTGTCTCCACTCTGTGC
TTATCTTGTCCTGGGAACAATTTTCCTACTGGCTGGATTTGTTTCTTTGTTTAGAATAAGA
ACTGTTATGAAACATGATGGTACAAAAACTGATAAACTGGAAAAATTAATGATCAGAAT
TGGAGTTTTTGGTGTTCTTTACACAGTACCTGCCTTGATTGTCATAGCATGTTTATTTTAT
GAATATGTTCATTTTGATAGTTGGATGGTAACATGGCATAGAGATTTATGTACAACTCCA
ATGTATTCAATTCCATGTCCTTTTTCTCATCAAGATGTAGTAAGACCTAAATTTGAAATGT
TTATGATAAAGTATCTCATGACAATGATTGTTGGTATCACTTCAAGTTTTTGGATTTGGTC
TAATAAGACTTTAGTTTCTTGGCATCAATTTTTTGACAAGATAAGAGGTAGAAGAGTTGA
AGCATATGTATGA

>frizzled2_Papilio_machaon_XM_045680138.1_ncbi
ATGTGTGCGGGCGTGCAGGGCGCGTGGCGCGCGCTGTCGGCGCTAGTTGCGTTGAGCTGT
CTGGTGGGATGCGGCGCGCTGCAGCAGCCACGATGCGAGGAGATCACCATCCCTATGTG
CCGCGGCATCGGCTACAACCTCACCTCATTCCCCAACGCCCTTGACCACGACACGCAGGA
GGAGGCCGGGCTAGAGGTGCACCAGTACTGGCCGCTGGTGGAGATCAAGTGCTCGCCGG
ACCTCAAATTCTTCCTGTGCTCGGTATACACGCCAATCTGCATCGAGGACTACGCCAAGC
CGCTGCCGGCGTGCCGCAGCGTATGCGAGCGCGCACGCGCCGGCTGCGCACCACTCATG
CAGAAGTACGGGTTCCAGTGGCCCGAGCGCATGGCCTGCGAGAAGCTGCCCAAGATGGG
CGAGCCCGACCAGCTGTGCATGGACGAGAACGAGCGAGCGCAGGAGCCGGAGCCGCCG
CGTCCGCCGCCGCGCCGTCCCCACAAGCCCTGCAAGGATCCAAAAAACTGCGAAAGCGC
TGCCGGCGGCGAGGGCGGCGGCGAGGAGTGCGCGTGCGCGTGCCGGGCGCCGCTCGTGA
GCGTGCGCGCGCACAACGCCAGCGTGCCCGGCCTCGCCAACTGCGCCGCGCCTTGTCGC
GGCCCCTTCTTCTCGCGCGAGGAGAAGGAGTTCGCGGCCGTGTGGATCGCGCTCTGGAGC
GGCCTGTGCGCCATCTCCACCCTCATGACGCTCACCACCTTCCTCATCGACTCGCAGCGC
TTTAAGTACCCCGAGCGGCCCATCGTCTACCTCTCCGCCTGCTACTTCATGGTGGCGCTG
GGCTACCTGGCGCGACTCGTGCTCGGCCACGACGAGATCGCATGCGACGGCGCCGTCAT
CAAGACCTCCGCCAACGGACCCAGCGCGTGCACCCTCGTCTTCATCCTGGTTTACTTCTTC
GGCATGGCGTCTTCGATCTGGTGGGTGGTGCTGTCGTTCGCATGGTTCCTGGCGGCCGGG
CTGAAGTGGGGCAACGAGGCGATCGCCGGCCACGCGCAGTACTACCACCTGGCGGCGTG
GCTCGTGCCCGCCGCCAAGACGGTGGCCGTGCTGCTGGCGGGCGCGGTCGACGGCGACC
CGGTGGCCGGCATCTGCTACGTGGGCAACTCCTCGCCCGAGAACCTGAAGAAGTACGTG
CTGGCGCCGCTGATCGTGTACTTCGCGCTCGGGGCGACCTTCCTGCTGGCCGGCTTCGTG
TCGCTCTTCCGCATCCGGTCGGTGATCAAGCGGCAGGGCGGCATCGGCGCCGGCTCCAA

GGCCGACAAGCTCGAGAAGCTGATGATCCGCATCGGCGTGTTCAGCGTGCTGTACGCGG
TGCCGGCCGGCGTCGTCATCGGCTGCCTGGCGTACGAGGCGGGCGGGCGCGCGGGCTGG
CTGCGGCGCGTCGCGTGCGGCCCCGCCTGCGGCCCGCGTCCGCTCTACTCCGCGCTCATG
CTCAAGTACTTCATGGCGCTGGCCGTGGGCATCACCTCGGGCGTCTGGATCTGGTCCGGC
AAGACGCTGGAGTCGTGGCGGCGCGTCTGGCGCGGCGGCCGCGGCCCGCACCGCGCGCT
CCCCAAGGGCGGCGTGTGA

>frizzled4_Papilio_machaon_XM_014502258.2_ncbi
ATGAAGTGCATTAACCAAGTTATTTATTGGTGACGCTCGTTATCGGAGCCAGCTCGGAA
GCTTCCGTGAGGACATGTGAACCCATCAAAGTGGCGATGTGCAAGAACATCGGTTACAA
CCAGACCGGCATGCCGAACTTGGCTCGGCACACGCTGCAAGTGGAAGCCGACGTCACGC
TCCAGACCTACAGTCCCCTGGTGCAGTACGGCTGCTCCTCGCAACTTCATCTGTTCCTCTG
TTCTGTTTACGTTCCGATGTGTACGGATAAGGTCGCGTTGCCGATCGGACCATGTAGGGG
ACTGTGTGAAAGCGTCTACGCGAGATGTTATCCCGTTCTGAGAGGGTTCGGATTCCCATG
GCCATCGGAGCTTGATTGTTCGTTGTTCCCGGCCGAAAACAACCACGAACATATGTGCAT
GGAGGGCCCGGGGGAGCGGGTACCGCCGGCGGGCCCGATCCCTCTGGACACAGCGGCG
ACGAGCGGGCGCGGCGCGTGCCGGCGGCTCGTGAAACCGAGCAGCTGGGTTTGGGTACG
CGGCTCGGGGCGGTGCGCGCAGTTCTGCGACGCGGAAGTGCTGTGGGAGGCTGGCGAAC
GGCGTGCGGCGGAGGTGTGGCTGGCGACGTGGGCCGCGCTCAGCTTCGCGTGCACGCTG
GCGGCGGTGGGCGCGCAGCTGGCGTGCGGAGCGGCAGGCGGGGCAGGCGGCGCGGGCG
AACGTGCTCTGGTGCTGGTAGCGCTATGCCGGTGCGCGGCGGCGGCGGGCTGGGGGGTG
CGCGCGGCGGCCGGTCGGTCCGCCGCGGGCTGCGCCAAGGACTCCATGTCTCCCACTCG
GATGTTGCTGGCTCACGATGGATTGGCAAATCCTAACTGCGCTGTCGTATTTCTACTCCTG
TACTATTTTGGATTGGCTGCATCTGTTTGGTGGGTGGTAGTAGCAGGTGCATGGCGTGCG
AGCGTGCTGCGACCCCCCGCGCCTGGCGCGCCGGGTACTGGGGGTGCTGGCGCATCTCGT
GGAGACCGGCACTCGTCGCTGCTGCAGCTGGCCGCGTGGGGGGTGCCCGCCGCGCTGGC
CGCCGCCGTGCTTGTCACCAGAGATGTCGATGCGGATGAACTAACTGGTACTTGCTTCGT
AGGGAACCAATCGAGCAAATCGTTGCTGGCTCTGGTCATCATACCGGAAGCAATTTGCTT
GCTGCTCGGCTGCCTGTTCCTGGCCTCGGGTCTGCGTACCGTCCTCCGACGACCGGCGCC
CGTGCCAGCGCCCGCAGCGCTTCTCAACGCTCCCCCACAACCACATACAGATCAGAGTTT
ACTAAGACTGGGTGCGTTCGCAGCACTATATGCTGTACCCTCAACGTGTATATTGGCAAC
TTGGATATACGAGTATACTTGGCGTGACGATTGGCTGGCCGCTCCCGAACCTTCAACTGA
ACCGTCGACGCAGCCACGGCCGGCATTCTGGGTGTTTCTATTTAGAATATTTGCAACTCA
AATTCTCGGCGTCATGGTCGCCGTTTGGATTGCGACGCCGCGTTTGAAAGCGCTTTGGAG
GAGAATTTCTGGTCCAACGAAGCCTACGCTAGCTAAATGTCCGGCGGGACCTACGCCTAC
CCCATTGACATTGCAATGTTACAGCACTCATTCGCACTCTCTACCACGACATACACACAA
GTATGCCACCTACAGACCACCACAGCACCAATCTTACAGGAAACCAAGGCATTATCATT
ATTCAGCCGGTGAAACCATACTATGA

>frizzled2_Tribolium_castaneum_XM_015977754.1_ncbi
ATGATGGGGTACACGAGGTTATCAATACTCGTGATACTCCTAGTGAACACCGCAACCGG
CGGCTCGCTAATATCCCACTTGGACGGCAGCGACCAAGAGGAGCGTTGCGAGGACATCA
CCATCCCCATGTGCATGGGCATCGGCTACAACCAAACTCGCATGCCCAACGAGCTCAACC
ACGAAACGCAAGAGGAAGCCGGCCTGGAAGTGCACCAGTTCTGGCCTCTGGTCGAGATC
AAGTGCTCCCCCGACTTGAAGTTCTTCCTCTGCTCCATGTACGCCCCCATCTGCCTCCCCG
GCTACAAGAAGCCTCTGCCCCCCTGCCGCGGGCTGTGCAAGCGGGCGCGCGAGGGCTGC
GAGCCCATCATGACCCAGTACGGCTTCAAGTGGCCCGAGCGCATGGACTGCGAGCAGTT
CCCCGTGTACGGCGCCTCCCCCGACCAGCTGTGCATGGACGAGAACAACGGCAGCAGCA
CCACGGCCAAGCCGCCGCCGCGCGCCAAGGCCCCCAAACAGTGCCGAGGGTCAAAAAAC
TGCACAAATCCCCCAGGAGACGGAAGAAAAAGCAGCGTGGCGAAGGGCTGCAAGTGCC
AGTGCCAACACCCGCTGGTCTCTCTGGGGAAGGACCCGTCACTGCTCAACCTAAGCGTCC
TCGACCCCGCCACCTGTGCCGCCCCCTGCCGCGCCACGTACTTCAACTCGGAGGAAAAAG
AGTTCACCACGGTCTGGATAACACTCTGGTCCAGCCTGTGCGCGGCTTCCACCTTAATGA
CACTTACGACCTTCTTCATCGAAACCGAACGGTTCAAGTACCCTGAGCGGCCGATTGTGT
TCTTATCGGCGTGCTATTTTCTCGTCTCGGTGGGTTATCTCATCCGGGTGGGGGTGGGGCA

TGAAGAAGTTGCGTGTGAAGGACCCGTTATCCGCTATTCCTCAACTGGGGCCAACCCCTG
CTCTTTGGTCTTCCTCCTAGTGTATTTCTTTGGCATGGCCTCGTCCATCTGGTGGGTGGTG
CTCTCCTTCACGTGGTTTCTAGCCGCCGGGTTAAAGTGGGGAAACGAAGCTATTGCCAGC
TATGCCGAGTACTTCCATGTCGCAGCTTGGCTTATTCCAACCTTGCAAACAGTCGCAGTG
TTTGTCTCTGGGGCCGTTGACGGCGACCCGGTCGCCGGTATTTGCTACGTTGGCAATATG
AACATGGAGAACCTTCGCACGTTTGTTCTGGCACCACTTTTCATCTACCTGCTCCTCGGTA
CCACTTTCCTAATCGCTGGATTTGTCTCCCTCTTCCGCATTCGCAACGTGATCAAGAAACA
GGGCGGCGCGGGCGCGGGCTCCAAAGCGGACAAGCTGGAGAAGCTCATGATCCGGATC
GGCATCTTCAGCGTGCTGTACACCGTCCCCGCGTCCATAGTCATCGCGTGTCACTTGTAC
GAAAACGCATTCCATAACGAGTGGATGCTCTCGGCCGCCTGCACCTGCCCCAGTGCGAA
AGTCCGCCCGATCTACTCCGTTCTAATGCTCAAATACTTCATGGCGTTGGCGGTGGGCAT
TACCTCCGGAGTCTGGATCTGGTCGGGCAAGACGCTCGACTCCTGGCGGAGGCTGTGGA
GGCGCCTCTTCGGCCGCCCCGACCCCACGGGGGCCAACGCCGTGCTGATCAAGAACCGG
CCGAAGGGGCTCGGGGGGCCGCCGCAGTACGTGGTGGCGGGCGCCGGGAGCGCGTCGCT
GCTGGGGCCCGCGGGCAGCGTGGCGTCGGCCAGTCAGCACCACCTCCACCATCACGTCC
TGAAGCAGCCCCCGTTGAGTCACGTATGA

>frizzled_Tribolium_castaneum_NM_001170776.1_ncbi
ATGAAGCCGCTGCTCCTGCTGGCGCTTTCGTCGATCATAAACGGCCTGCAGCACGAAATC
TCCCACATCGAAGACGCAATAATTCCACCCCATGACAAGTGCAAGCCCATCACTGTGCCT
TTCTGCATCGACGTCCCCTACAACAGCACCATTTTCCCCAACCTGGTGGGCCACAACACC
CAGGAGGACGCCGGCTACGAGGTGCACCAGTACTTCCCCCTGATCAAAATCAACTGCAG
CGCCGACTTGCACCTGTTCCTCTGCTCCGTGTTCGTGCCCGTGTGCACGATCTTGGAGAA
GCCGGTGCCCCCATGCCGCTCCCTGTGCCTCTCGGCCAAGTCGGGCTGCGAGGGGATCAT
GCGGAAATTCGGCTACAACTGGCCCGAAAACCTCGACTGCAACCAGTACCCCGAAAACT
CGAATCTCTGCGTGGAGAAACACAACATCAGTGTGACCCCCACACCGGCAAACGAGCCC
CCCGACTACCCCAAGAGCAAGTTCGATTTCAAGGGGGTACGGGCGAAGTATCCGCGGGA
TTTCGGGTTCGTGTGCCCCCTGCAGTTCGAGGTGAAGAACATGGACTACTCCCTTAAAGT
GGGGGACAAAGTGGTGAAGAACTGTGGGGCCCCGTGCCACATGATGTTCTTCAACGAAA
ATAAGCAGAAATTCTCCCGGGTCTGGATTGGGACTTGGGCTGTTTTGTGCTCGGCCAGTT
GTCTGTTCACTGTGTGCACCTTCTTGATTGACACCAATAGGTTTAGGTACCCCGAACGCC
CCATTATATTTTTATCGGTTTGTTATTTGATGGTCGCTTTGGCGTACGTTGTCGGTTTTATC
GCCGGCGATTCCATAGCGTGTAAGGAACCGCCGATTTCGGATGAAGGCATCAGGAAAGT
GAGCATTATCACACAAGGGACCAAATACGAATTGTGCACGATTTTATTCATGGTCTTATA
TTTCTTCAGTATGGCCTCGAGTATTTGGTGGGTGATTTTGACTCTGACGTGGTTTTTGGCC
GCTGGGTTGAAATGGGGGCATGAGGCGATTGAAGCCAACTCACAGTATTTTCATCTGGC
GGCATGGGCGATTCCGGCCGTTAAGACCATCACGATTTTGGCGATGGGAAAAGTCGATG
GTGACGTACTAAGCGGAGTTTGCTACGTTGGAATTTGGAACGTGGACGCCTTGCGAATCT
TCATTCTCATACCTTTATGCATTTATCTAGTCTTCGGGTTAGTATTTTTAACTTCCGGCTTA
ATCTCGCTATTCCGTATTCGTACGGTAATGAAACACGACGGTACAAAAACCGACAAATTA
GAAAAGCTAATGATCCGTATCGGCGTATTTTCTATTTTATACACAGTACCTGCAATGATG
GTCATTGCTTGCCTGTTTTACGAACAGTTTAATTTCGATAATTGGATGTTGACTTGGAATC
ACGACATGTGCGTCAAACAGACCTATGCCGTGCCTTGTCCGCAAGACATTAGGGAATATT
CACATCCGCTCTTTCCCATTTTTATGATCAAATATCTCATGTATATGATTGTGGGAATAAC
GTCAAGTGTGTGGATTTGGTCGGGCAAAACGGTGCACAGTTGGAAAGTGTTTCTTAACCG
ACTCAGAGGCAGACATGCGGCTTACGTTTAA

>frizzled4_Tribolium_castaneum_XM_962510.3_ncbi
ATGGTTTCGTTCGTGCTGTTCGCGCTCGTGTCGGCGGCCTGGGCCGAGCCCCTGGTGCGC
ACCTGCGAGCCCATCCGCGTGGACATGTGCACCAACCTGGGCTACAACATGACGGAGAT
GCCCAACCTCGGCGGCAACGACATCCAGCAGGAGGCCGACTACACGCTCAAGTCCTTCT
CGCCGCTGATCCAGTACGGCTGCAGCTCGCAGCTGAAGCTGTTCCTGTGCTCGGTGTACG
TGCCCATGTGCACGGAGAAGGTGGCCAACCCCATCGGGCCCTGCCGCGGGCTGTGCGAG
AGCGTGCGGGCCAAGTGCTACCCCGTGCTCAAGGGCTTCGGCTTCTCCTGGCCGGACGCC
CTCAACTGCTCGCGCTTCCCGGTGGAGAACAACCACGAGCACCTCTGCATGGAGGGCCC

CAAGGACCGGGGGGTGGACGTGCTGGCGCCCGTGGACCCCGCCGTGCAGAAGTTCGACT
GCGGGAGGAATTTCGTCAGGAATGAGTTGGGGAACTGCGTGCCGGCCTGCGATACCAAT
CTCATGTTTGACGATTCGGAGAAGAACTTTGCCGAGGTGTGGGTGACCGTGTGGGCAATG
ATTTGCTTCGTGATCAGCTTAGCCTCGGCCCTGACGCTGACAATAGGCGGAGGCCGCGTG
AAGGCTCGCCCTTTGATCAGCATAGCCCTTTGCTACTGCATGGTGAGCGCTGGCTGGGCC
CTCCGCATGTTCTCGGGCCGCATGTCGCCCTTCTGCCCGCCCGAGGACGGCCTTTCGAAC
GTCAACTGCGCGTTCGTCTTCCTCCTGATCTACTATTTCGGGATGGCAGCGAATGCTTGGT
GGGTCTGTTTATGCGCTTGGTGGGTCGCACGTGTGGGACTGGCTTGGCCTCCGGAAAAGC
TCCGAAGCCTGGGCTCGGTGCTGCATGTGTGCGCTTGGGGCTTGCCGGCAGCGCAGACTG
TCGCCGCCTTGGTGCGGCGGGACGTGGACCCGGACGAGCTCACTGGGACCTGTTACATC
GGCAACCGCAACTCGGCCACCCTCCTCGGCCTGGTGCTCATCCCCGACTTCATCTACTTT
ATGCTGGGAATATTTTTCTTAATCCTGGGCTGCGTGCGCGTGATCCGCAAGCCGCGCCCC
TCGGCGGCGGCCCCCTTGACGGCGGCGACGCCCCGCAAAGAGAGCGACTTCCTGGGGGC
CGTGTGCACCCTCTACGCCATCCCCACGTTCTGCGTCATGGCCAGCATCTACTACGAGTA
CAACAACCGCGACCAGTGGCTGAACCGCGAGTCCAAACCGGCCTTATGGGCCTTCCTCCT
GCGCCACCTCATGTCGCTCTTCATCGGCGTCAGCACCATCTTCTGGATCTGGTCGATGAA
GACTGTGACGGCCTGGCGGGCGGTTCTGCGCCGGCTGGGCCCCAGGAAGCAGCTGCCGG
TGAAGGTGCAGACGATGCCGGTGCTGCGGTACGTCCCGGCGCACCCAGTGCCCAGCACC
TTCAGCACGAGCAGCAGGCACTCGACCAGGTCGCACCCGCACCGGAAGCCGAGAGTGCA
CCACATCCGGACCGGCGGGGAGACTGTTATTTGA

>frizzled2_Bombyx_mori_XM_038013768.1_ncbi
ATGTTATATGCGGTAAACGTAGCGCAGTCCAGGATGTGGGCAGCGGGCGTGGCGGCGTG
CGCGCTGCTGGCCGCCGCCGGAGCCCTGCAGCAGCCGCGCTGTCAGGACATCACCATCC
CCATGTGCCGCGGCATCGGCTACAACCTCACTTCCTTCCCCAACGCGCTGGACCACGACA
CGCAGGAAGAAGCCGGACTAGAGGTTCACCAATATTGGCCCCTCGTCGAGATCAAGTGC
TCGTCGGACCTAAAGTTCTTCCTGTGCTCCGTGTACACGCCGATCTGCATCGAGGACTAC
GCGAAGCCGCTGCCGGCGTGTCGCAGCGTGTGCGAGCGCGCGCGGGCCGGCTGCGCGCC
GCTCATGCAGCAGTACGGGTTCCCGTGGCCCGAGCGCATGGCGTGCGAGCAGCTGCCGC
GCGCCGGCGACCCCGACCAGCTGTGCATGGAGGAGACGGAGCGCGCGCACGAGCCCGA
GCCGCCCCGCCCGCCGCCCCGCAAGCCGCCAAGAACAACTGCAAGGATCCAAAAAACT
GCGGCGGCGCGGCGGCTGCGGGCGCCGGCGACGCGGCCGAGTGCGAGTGCGCGTGCCGC
CCGCCGCTGCTCGCCGCGCCCAACACCAGCGCCGCGCCCGGCCTGCGCCCCCCCGCCTGT
CTGCAGCCCTGCCGCGGCGCCTTCTTCACCGCCGACGAAAAACACTTCGCGGCCGTCTGG
GTGGCGCTGTGGAGCGGCCTCTGCGCCGCCTCCACGCTCATGACGCTCACCACCTTCCTG
ATCGACTCGCAGCGGTTTAAGTACCCGGAGCGCCCCATCGTGTACTTGTCGGCCTGCTAC
TTCATGGTGTCGCTGGGCTACCTGGCGCGCCTGGCGCTGGGGCACGACGAGATCGCGTGC
GACGGGCCGGCGCTCAAGGTGTCCGCCAGCGGACCCAGCGCGTGCACGCTCGTCTTCAT
CTTGGTCTATTTCTTCGGAATGGCGTCGTCGATCTGGTGGGTGGTGCTGTCCTTCGCGTGG
TTCCTCGCCGCCGGACTCAAGTGGGGCAATGAAGCCATCGCCGGTTACGCACAGTACTAC
CACCTGGCCGCGTGGCTCGTACCGGCTGCGAAGACGGTCGCCGTGCTGCTGGCCGGCGC
CGTTGACGGCGACCCGGTGGCCGGGATCTGTTCAGTCGGCAACTCTTCTCCGGAAAATCT
CAAGAAATTCGTGCTGGCGCCGCTGGTCGTGTACTTCGGTCTGGGCGCGACGTTCCTGCT
CGCCGGGTTCGTGTCGCTGTTCCGCATCCGGTCGGTGATCAAGCGGCAGGGCGGCGTGG
GCGCGGGCTCCAAGGCGGACAAGCTGGAGAAGCTGATGATCCGCATCGGCGTGTTCAGC
GTGCTGTACGCGGTGCCGGCGGGCGCGGTGCTGGCGTGCCTGGCGTACGAGGCGGCGGG
GCACGCGGGCTGGCTGCGGCGCGCCGCGTGCGGCTCCCGCTGCGGGCCGGCGCCGCTGT
ACGCCGCGCTCATGCTCAAGTACTTCATGGCGCTGGCGGTGGGCATCACGTCGGGCGTGT
GGATCTGGTCGGGCAAGACGCTGGACTCGTGGCGCCGCGTGTGGGCGGGCGGGCGGCGC
GTGCCCCCGCCCTCGCAGCGCGCGCTCGTCAAGGGCGCCGTGTGA

>frizzled7B_Bombyx_mori_XM_004929223.4_ncbi
ATGATCAATATGGCAAAATTTTGGAAACATTATGTTATTATATTCAGTCTGTTATGTCTTT
TTGCGTTAACAAAAGGTTCTCGAGAGATTGTTTATCAAGGCGATAGTCTACCTCAACATG
GACGTTGTGAACCCATTACCATTCAGTTTTGTCAGAAATTGCGTTATAATCAAACCATTTT

TCCGAATATTTTAAATCAAGCACGACAAGAAGATGCAGCCGCAAACATGCTTCTGTTTAC
AACTCTCATTAAACTGAATTGCTCTCCTGATCTAAGATTCTTCTTGTGTTCAGTTTATGCA
CCCGTTTGCACGATACTAGATTCTGCGATACCGCCTTGTCGTCATTTATGTGAAGCTGCA
AAACAAAGCTGTGATGTCGTCATACGAAAATTTGATTTTCCTTGGCCCCCTGAGCTTGAA
TGTTCTGAATTTCCTGAAGTATCTGATAATAACATATGTGTTGGTGACAATAATACTGCC
CATGAATCTCGAAAACGCACTGATACCAAGTTTCGTAAGGTTGACTTCAAATCTAACATG
GAGAGAGATCCGACTAGATTGCATGAGTTTGTGTGCCCAGTGCAGTTTAAAGTACCAAG
ACATTTAGATCTTGAATACTCTTTTAAAGTTGGAAATAGAGTTGAACATGACTGTGGAGC
CCCATGCAATGGAATGTTCTTCAGTAAAGATGAAAAGAATTTTTCAAGGCTCTGGATTGG
AATCTGGGCAGTCCTGTGTGCACTCAGTTGCCTTTTTACTGTGCTAACATTTTTAATAGAT
ACAGATAGATTCAGGTATCCAGAAAGACCCATAATATTTTTGTCAGTTTGTTATCTAATG
GTAGCTATTGCCTACATAATGGGTTGGAGTGCAGGTGATAATATAAGCTGCCAAGGGCCT
TTTGTTTCTGCTGTCAGTGGAACAAGATTACCCAACATATCAGTTATCACACAAGGTACA
AAACATGAGCCATGCACAATACTGTTTATGATAGTATATTTCTTTAGTATGGCTTCGAGT
ATATGGTGGGTCATATTAACCCTGACATGGTTTTTGGCTGCTGGCCTAAAATGGGGACAT
GAAGCAATAGAAGCAAATTCCCAATATTTTCATCTTGCTGCTTGGGCAGTACCAGCTGTG
AAGACAATATCTATTCTAGCAATGGGGAAAGTAGATGGTGATGTACTGTCAGGTGTATGT
TACGTTGGATTATGGGATGCAGAAGCTTTAAGAGGATTTGTGTTGGCACCTTTATGTGTC
TATTTAGTGTTTGGAACAATCTTTCTTCTAGCTGGCTTTGTCTCACTGTTTCGGATCAGAA
CTGTCATGAAACATGATGGGACTAAAACTGATAAATTAGAGAAACTGATGATTCGCATT
GGTGTGTTTGGTGTACTATACACCGTGCCTGCCCTCATAGTCATAGCATGTTTGTTCTATG
AACAAGTTAATTTTGACAAATGGATGATTGCATGGCAACGAGACATGTGTTCGATACGA
CAATACTCAATTCCTTGTCCGATAATTCATGAAGAGATAGAAGGACCAAAGTTTGAAATG
TTTATGATTAAATATCTAATGACAATGATTGTTGGTATAACATCTAGCTTTTGGATATGGT
CTGGTAAAACTTTGATATCATGGTCTCAATTTTTTGACAAAGTTAAAGGTCGCAGAGTAG
AAGCCTATGTGTAA

>frizzled4_Bombyx_mori_XM_012690483.3_ncbi
ATGTTGTGCATTATAGCGATTCTGTTTACTATTTGCCACGTTCCGCGCGCCATCGCCGAGG
CCTCCGTGCGGACGTGCGAACCCATTAAAGTGGCAATGTGCAAGAACATCGGCTACAAC
CAGACCGGTATGCCGAACTTGGCCCGGCACACCCTGCAAGCTGATGCCGATATCACGTTG
CAAACATTCAGTCCGCTGGTCCAGTATGGCTGCTCTTCCCAGCTTCATTTGTTTCTGTGTT
CGGTCTACGTTCCCATGTGTACGGATAAAGTGGCGCTGCCGATCGGGCCCTGTCGAGGAC
TGTGTGACAGTGTGCACGCCAGATGTTTCCCTGTGCTTCATGGTTTCGGATTTTCTTGGCC
CCCGGAGCTAGATTGCACACTGTTTCCAGCGGAAAACAATCACGAACACATGTGTATGG
AGGGGCCCGGCGAAAGAGCCCCACCGGTCGGGAGTATCCCGTTGAACGCAACGGGTGCG
TGTAGGAGATTAATCAAACCAAACAGTTGGGTGTGGGTTAGAGGATCAGGCCGCTGCGC
CCAGTTTTGTGATGCCGAAGTCCTGTGGGAAACTGGTGAACGCAGAGCGGCGGAAGTAT
GGCTCGCTACGTGGGCAGCATTAAGCTTTGCTTGCACTTTGGCAGCTGTCGCTGCGCAAT
TAGCATGCGGAGATCGCGGAGGAGCTGGAGAACGAGCACTAGTGCTAGTGGCTCTGTGC
AGGTGTGCTGCAGCGGCTGGATGGGGCGTTCGCGCTGCTGCAGGTCGAGCTGCAGCCGG
CTGCGCCAAGGACTCCACATCCCCGGCACGCATGCTTCTAGCACACGACGGACTTGCGA
ATCCAAATTGCGCTGTTGTGTTCTTGCTACTCTACTATTTCGGTCTTGCTGCATCAGTATG
GTGGGTGGTAGTGGCGGGCGCTTGGCGAGCAAGCGTACTCCGACCACCTAATCCACCGG
GTACAATGGCGAGAAACGACCGCCACTCATCCCTGCTTCAGCTGGCGGCGTGGGGCGTG
CCGGCAGCCCTAGCAGCAGCCGTTCTCGTCACCAGAGACGTAGATGCCGATGAACTTAC
AGGTACGTGCTTCGTCGGCAATCAATCGACCAAGTCCCTCCTTGCGCTCGTAATCGTACC
TGAAGCAATATGTCTGCTACTCGGCTGCGTGTTCCTTTCGTCCGGACTACGCGCTGTTCTT
CGCAAACCAGTTCCCGTGCACACACCAGCCCTATTATTAAACGCTCCACAGCCGCACACA
GATCAAAGCGTTTTGCGTTTAGGTGCTTTCGCAGCTCTATACGCTGTTCCGTCGGCGTGTA
TTCTCGCTACTTGGGTTTATGAATACGCGTGGAGAGACGAATGGCTGGCTTCACCGGTTC
CGTCTTCGGAGCCATCGACACACGCCCACCCAGCATTCTGGGTGTTCCTATTTAGAATAT
TCGCTATTCAAATACTCGGTGTGATGGTGGCCGTTTGGATAGCAACGCCCCGCCTTAAAG
CTCTGTGGAGAAGGGTGACGGGACCTAGAAAGCCAGTGCCTCCCAAATGTCCGACGGGG
CCGACACAAGCGCCCCTAACTCTTCATTGTTACGCAACACATCCTCATACGATAGCGCGA

CATCCTTATAATCCAACAAAGTACGCAACATACCGACCTCCGCAGCAGCATTCGTACAGG
AAACCTAGGCATTATCATTATTCAGCGGGTGAAACTATACTATGA

>frizzled9-like_Athalia_rosae_XM_012395440.3_ncbi
ATGAACCCCAACATATTTTTTCTACTACTCGTAACTACAACTCTGTTAGACACCTTTTTCG
GTGGTTCATTAGGACCAACCGTTACCATGGCGTGGGGAATTAACCACGGAACAACTGGT
TTATCATCTTCATCTGGAACGTTGGGTTCATCTGGTGGTAACAACAATTACGCAAAGTGC
GAAAGGTTAACCGTATCAATGTGCAGAGGACTTGGTTACAACCTTACGGCTATGCCAAAT
TTTATGGGGCACGAAGACCAGCTCCAAGCCGAACGTGGGCTTGCAGCATTCATGCCATTG
GTGCACTACAATTGTTCTCGTCACTTGAGGCTCTTTCTTTGCGCAGTTTTTGCGCCAGTTT
GCTCCGAACATGTCGCGATGCAAATACCTGCCTGCAAACCCCTCTGTTTATCGGTGAGAC
GAGATTGCGAGCCCGCTTTATCCAGTCTAAGCTTACCATGGCCTAATATGCTCGACTGCG
AGCGATTTCCCGACGGAAAAACCGTTACGCTTTGCGTCCAACCGTTTGCCGAAGAATCCT
TATCCGCAGAACTTCCGGCAGTTCCACAGGTAATTCCTTCGTTACAAACAGCACCGATTC
TTCAACCACAATGGCCACCTGCACTGATGCAACCGATCCAGTCTGCACAGACGATTTTAC
CGGCAGTTCGAACAAATCAAATCCATCATCAATGCCCTCCACATTTTGTTCAGACGCCCG
ATGTTGAAGCGGTCACTTGCGCACCTCATTGTGGATCGGACGCATATTACAGATCAGAAG
ATAAAAAGTTTGCCGAACGTTGGATGACGGGTTGGGCATGGTTGTGTTTCCTCTCGACCT
TATTTACGTTATTAACATTTTGGGTAGAACCATCGAGATTTCGTTACCCAGAACGGCCTA
TCGTCTTCTTAGCACTTTGCTACAATCTTTTATCGGTAGCTTACACGATTCGCGGTGCGAT
TGGCGCTGAAAATCTCAGTTGCACCACGCAGGACGATGGCATTAGCTACGTACCGGTCA
ATGATGGACTCAGGAGCGTCCCATGTACACTTTGGTGGCTGGCAAGACATTATCTTGGAC
TGGCCAGCAGCGCCTGGTGGGCGGTTCTTTGTGGCTGTTGGTTACTGAGCGCCAGAAATG
AGTGGAGCAGCGAAGCTCTTCACAATATTGCACCATATCTTCACGCAACTGCTTGGGGAA
TACCAGCCCTGATAACTGGAGGTAGTTTATTATCGCGCAGTGTATCGGCCGACGAGTTGA
CCGGCCTTTGTCAAATATCGGACGATTCAGCTCTATGGCTTGAAGTTCTTCCGCACGCAA
TTCTCCTATTATTGGGTTGCATTTTTGCGAGTGTGGCAGGGCTAGCTTTAGTCCGTGTAAG
ACGTGCGGTTCGATTAGCCGGTCGTTCTGCTACTAAACTCGAGCGTCTAATGACTCGCTT
GGGAATATTTGCACTTTTATATGCTTTACCTGCTCTCGGAGGGCTAGCGTGCGTTCTGCAC
GAATCTTCAGTCAGACCAAGATGGCGAACGCTCGCGTTACTTGCAGCTCTGGATTGCAGA
GCCACCCAAAATTGTTCACCGGGTCCAGTTTACAGAGCAGCTGGACTTGAAGTCGTACTT
TTGAGATTGTTCCTTTCGCTAGTCGTCGGCGTTACTTCAGGAATGTGGGTATGGTCAGGA
AAAACTTGCAGAGCTTGGAGCAGACTTTTAGCTGCACCTAGTAAACCGCTTAGGCCGCA
ACAAGGCATGCAACATATCGGACAAAATGTTTATCAAGGTTATAAACCAGAAAGCGCAA
ATATGGCGTAG

>frizzled2_Athalia_rosae_XM_048651491.1_ncbi
ATGGGACCTTGTCGTCGGACGGCGGGGTCGCTGGTCTTCGCCCTTTTTCTACAGTCAGTC
CTTCTCCACAATGTCGAATCCGAGTCAGCGGTGGGCAGTCCGTCGAACGGCAGTCCAGG
GGTTGCGGGTTCTTCCGGATCGTCAAGTGTCGGGGGCGGTCATCCAGCGTTGAGCGGTTC
GCCTGGTAACGGGAGATGCGAGGAAATAACGATACCGATGTGTAGATCCATCGGTTATA
ACTTGACCGCGATGCCGAACGAGCTCAATCACGATACACAGGAGGAAGCTGGTCTCGAA
GTCCACCAGTTTTGGCCTTTGGTGGAAATAAAATGCTCACCAGATCTAAAATTCTTCCTA
TGCTCAATGTACACACCGATCTGTCTTCCGGAGTACACGAAACCACTTCCGGCATGCAGG
AGTGTATGCGAAAGGGCCAGGGCCGGTTGCGCCCCTCTTATGCAACAGTACGGGTTTTCG
TGGCCCGAAAGAATGGCTTGCGAACGTCTACCGTATCACGGTGATCCTGAAAATTTATGC
ATGGAACAAGACAACCACACGAGCAGCGCTGCCAGTAGCGCACCGATGCCGGCTCCGCC
GCGTCCAACTAAACCATCGAAACCGTCGCAGATTCCGAGGTGTAAGCCGGGTAAAAACC
AAAAAAACTGCCAAAATCCCCCAGGGGACAGGGCGAGGGAGTGCGCGTGCCGATGCAG
GGCGCCTCTCGTGCCTCTGGGGGCAAACGGGGTAGTCAGCAGCGTGGTCGTCGCGGCGG
GTCCGGGACTGGGAGTGGGTCTGGGACTCCCTCAGGACATTGCAGGGGTACCGGATTGC
GCCCTTCCGTGCCACGGCGCGTTTCTAACACACGAAGAGCGGGGATTCGCGGCGGTATG
GCTCGCCCTTTGGAGCGGTCTGTGCGCCGCAAGCACACTCATGACCGTAACCACCTTCCT
CATAGACACCCAGCGCTTCAAGTACCCTGAAAGACCGATAGTATTTCTGTCGGCTTGTTA
CTTCGTCGTATCCCTGGGATACCTCGCTCGCAGTGTATTGGGACATGAGGAGATAGCTTG

CGACGGACTCGCCCTGAGATCCGGAGCGAAAGGACCCGGAGCTTGCGTGACCGTTTTCC
TGATGATCTACTTCTTCGGTATGGCATCTTCGGTATGGTGGGTTATCCTGGCTTTCACCTG
GTTCCTTGCCGCTGGTCTGAAATGGGGCAACGAAGCGATAGCTTCGTATTCTCAATACTT
TCATCTAGCGGCTTGGTTGGCACCAACAGCTCAAACAGTGTGGGCACTTCTCGCCGGCGG
AGTAGCCGGTGACCCTGTAGCCGGGGTTTGTACCGTCGCTCCGGAAGGCGTAAGGACAT
TTATTCTCGCACCGCTGCTGGTATACCTATTGTTAGGTACTAGTTTTCTGCTTGCCGGTTTT
GTAAGCCTCTTCAGAATTCGTTCAGTGATAAAAAGACAGCCCGGTGCTAAGGCTGACAA
ACTTGAAAAACTGATGATTCGTATCGGCGTTTTCTCCGTACTCTACACCGTCCCAGCTAG
CGCTGTATTAGCGTGTCACCTTTACGAATCCACGCTACGAGACGAGTGGTTAGGATCACT
GGCGTGTCCCTGCAGACCAAGATCTCGACCATTGTACTCCGTACTGATGTTAAAATACTT
CATGGCTCTGGCAGTCGGTATAACATCTGGCGTTTGGATATGGAGTGGTAAGACATTGGA
TTCGTGGAGGAGACTTTGGCGGAGGCTTTTCGGTACGGGACCAGGAAATACGGGAATCA
AGGGTGGAGTTGGCGTCGGCAGAGCGGGTCCGCAATATCCACCTCCCCCGGCCGGAAGC
GCTCTTTTACCTCCTGGAAGCGTCGCCAGCGCTTCTCAACACCATTTACACCATCACGTTC
TCAAGCAACCGCCTCTCTCGCACGTATGA

>frizzled7_Athalia_rosae_XM_012400624.3_ncbi
ATGTTCGCGTACCTTTTCCTGAGTTTTTACGTAGCTAACGTGAATGTGAACGTGAACGCA
CTCCATGCACGGGACCCATTGGCACATCATGGACGTTGCGAACCGATTACGATTCCTCTC
TGCATGAACATTCCTTACAATGAGACGATTATGCCAAACTTAATGAACCACCAGAAGCA
GGAGGACGCAGGTCTCGAAGTTCATCAATTCCTGCCACTGGTTAAAGTCAAATGCAGTCC
CGACCTGCAATTCTTTTTGTGCTCAATGTACGTTCCTGTCTGCACGATTTTGGAGCAGGCA
ATTCCGCCCTGCAGATCCCTTTGCGAGAGTGCAAAAGCTGGTTGTGAGTCATTAATGAAT
CGTTTTGGGTTTTTGTGGCCTGACAATCTGGAGTGTTCGAAATTCCCAGTTTCTGGTGGTA
CTGAAATTTGCGTTGGTGATAACAACACAGCTTCCACCGAAGCCCCTGTTCTAGTTGATC
CTCCACCTCGAATACCAATAGCAGAGTTTCATCCTGGATGGAATGGTGGGATCAAATCTT
ACAGTGGAGATTTACTTGGAGCTAGAGATTATGGATTCGTCTGCCCTGTCCAATTCAAGG
TACCTCGAGAGCTCGGCTATAGTCTGAAAGTTGGGGACAAGGTAGAGCCTGACTGTGGT
GCTCCTTGCGACGACATGTTCTTCACAGGCATCGAGCGTAGGTTCTCGAGGATCTGGGTT
GGGGCTTGGGCCTCAGTATGCGCAGTATCCTGCTTCTTCACTGTTTTAACTTTTCTCATCG
ACACTGATCGCTTCCGCTATCCAGAAAGGCCTATTATATTTTTATCTGTCTGTTACTTGAT
GGTGGCTCTGGTTTATGTGATCGGATGGGCTGCAGGCAACTCCATATCATGCAGAGAACC
TTTTCCCCCACCTGTAGATCTCAGGATTGAAATGGCATCAACTATAACCCAGGGAACAAA
GCATGAACTTTGCACCGTACTTTTCATGGTGCTTTATTTCTTCAGTATGGCATCGAGCATA
TGGTGGGTCATCCTCACTTTGACGTGGTTTCTTGCCGCCGGACTCAAATGGGGACATGAA
GCTATCGAATCTCATTCCCAGTACTTTCATCTTGCTGCATGGGCCGTTCCTGCAATCAAAA
CCATCACCATACTTGCAATGGGGAAGGTTGAAGGGGATATATTGTCAGGAGTGTGCTAC
GTTGGATTATGGAATGTTGAGGCACTGAGAGCCTTTGTCTTAGCGCCGCTGTGCTTCTAC
CTGGTCCTTGGAACAGCCTTTCTTCTCGCAGGATTTGTATCACTGTTTCGTATTCGCACAG
TCATGAAACATGATGGTACAAAAACAGACAAACTAGAAAAACTAATGATACGTATTGGA
ATATTTTCTGTACTTTACACGGTACCTGCTCTGATTGTTATCGCTTGTTTATTCTATGAACA
AGCCTACTTTGATCGTTGGATGCTGCAATGGAACATGGAAATGTGTTCTCGGCCCGGGCC
GCAATCTCTTTACTCAATTCCTTGCCCTGTTGGCGAACGTACACGTGACCTGGGACGCCG
TCCAGAATTTGAGGTTTTTATGATTAAGTATTTGATGGCGATGATAGTTGGGATAACAAG
CAGCTTTTGGGTGTGGTCAAGCAAAACCCTCACTAGCTGGCGTCAATTCATTCATCGTAT
CCAAGGACGTCGTGCCGAAGCTTATGTTTAG

>frizzled4_Athalia_rosae_XM_012395305.3_ncbi
ATGTCGTTTAAAAATGTTATCTCGTTTTCGTTCTTGTCTATATTATTGGTGTTGATGAACTT
TTCGGTGCCAATTTACTCTACTCATGGTGTATGCGAACCAATAAGGATTGAAATGTGCCG
TGGTGTTGGTTATAACGTAACGGCGATGCCAAATTTAGTTGGAAATGAAATTCAAAGGG
AGGCTGATTTTACCCTGCAAACTTTCAGCCCTTTGATTCAATACGGATGCAGCGCACAGC
TTCATTTGTTCCTGTGTTCAGTGTACGCCCCGATGTGTACCGAAAAGGTACCTTCGCCGAT
CGGTCCTTGCAGGGGTCTTTGCGAGCAAGTTAGAGCCAGATGTTACCCAGTTCTATTGGG
CTTTGGTTTCCCATGGCCAGCCGCTTTGAATTGCTCGAAATTCCCACCCGAAAACAATCA

CCAACACATGTGTATGGAAGGACCTGGCGAACCCGGACCCGCCAATCCAGTCCAGGCGA
TCGGCGACGGGAACGGTCCCTTGGGATGTTCCTGGTATGCGAAATCAGGTCTCTACGTAT
TTCTGAATCGTTCCGGTCGTTGTGCCGCAACTTGTGACGCGGATGTGTTATGGTCTCAAA
AGGATAAGAGATTAACAGAGGGTTGGATAGCGGCATCGGCCACCGTCTGTCTCATATCA
GTGGCAGCGGCGATCCTGGCTCTCCTGAAACCGTACAAAAATCAACACCCTCGGGGCGT
CGCGGACTCCGTAGAAAGAGCAATAGCGTGTCTCGCCCTATGTCACGCGGCGGTGGCAG
TTGGTCATGTGATACGTTTGGCAGCCGGGAAATTTGCGGTCGCTTGCACACCAGCGATGT
CTCTTCACCCTCCAGAACTAGTGGTAATAGCGCAACAACAGCATCAGTACCTCACTCAGG
ACGGACTCTCTAACCCCTACTGCGCTCTTGTATTCCTCCTCAGATATTACTTCGGAAACGC
CGCCAGTGTATGGTGGGTAGTCGTCTGCGCATGGTGGTGTATTTCGACAAGAAGATGGAC
TAAATCGAAGTCCTTGGGATTGGCTGGAGGCAATTCAACCTCTAGGGAATTACAAGATC
AAGGTTTTGGATTACAACAAGGATGTTCGACACTCGGTACAGTAGCTTCCTGGGGATTAC
CTGCCGTTCACACTATTGCCGTACTTGTAACGAGGGACGTCGACGCGGACGAGCTAACTG
GAAGTTGTTTCGTCGGTCAGCAAAGCACGAGATCACTCTTGGTCCTTGTATTAGCACCTC
AGTTCGTCTACATATTTTTCGGGCTGTCTTTTCTGTTGACCGGAATGATGACTCTGCTTTT
ACCTCGACCATCGGTGACCCCCTCACCAGTCTTGAACCCTTTGACCCCTGCTAGCACCCT
GGGTAGCAGACAACAGCGCAGTAACAATTTTCTTGCTAAAATTCCTCCAGTCATTGACTC
TCAACAAAAACAAAAATCAGCTTTAGTGCGAATGGGAATATTTGCCTGCGTTTACGCTGC
AGTAATTATAACTGCTGCTGGAATCAGTTTTTACGAATGGTGGAACAGAGATTCTTGGCT
TAGGGCACCAGAGCCATCGACGTCACCTAAAGCACCATCAAAGCCGTTGCTCCAACTTTT
TCTTATACGATCGGTCGCAACTTTGGTAGCTGGGGTAATGGCAGCGGCATGGATTTGGTG
GCCTAATGTCGTATATATTTGGCGACGTTTACCACCCTGCAAGCAGCCACCTCATAAATG
TCACCCTCACACCGGACTTCAAGTTCCTGTTGTCAGATGCTACAGCGCAGGATCTACGAC
CACCAACTCACACCAAATTCATCACCTCAATCACCTAGCGCCGCAACATCCCCTACTCAG
GACTACCACTCCTAATGTTAGTAATGGACAAATTATTCAAGGTCCTTACAGAAGCCATAA
AAAACATAGAAAACATAGGAAACATCACAGTGGTAGCGAAACTCAAGTCTAA

>frizzled9-like_Solenopsis_invicta_XM_011169335.3_ncbi
ATGCCATCGACATTGAAAATGGTGCTACGCATCGTGGTGCTCATCCTTTCGGCAGCGTCG
ATCCGAGGATGGGGCATCAACCACGCGCATCCTGGGACCGGTGCCAAATGCGAACGACT
GAACGTGTCCTTCTGCCGGGGATTACGTTATAATCTGACGGCGATGCCAAATTTCATGGG
TCACGAAGATCAGCGGCAAGCAGAACGAGGGTTGGCTGCCTTCATGCCATTGGTCCACT
ACAACTGTTCGAGACATCTCAGGCTCTTCTTATGCGCTGTCTTCGCCCCAGTCTGCTCGGA
GCATGTGGCCATACCGGCCTGCAAGTCTCTCTGCCTTTCCGTAAGAAGAGACTGCGAGCC
GGCCTTAACCAGCCTCACGCTGCCGTGGCCGCACATGCTGGATTGTGATCGCTTCCTTGA
TCGCGGAAATACATTGTGCGTGCAACCGCCGGAGGAAACATTGGATGACACTAGTTCAC
CGATACTGCCAACTGTTCAGCAGCAGTGGCCGATGATACACGAACAATCACTGAAAATT
TCTACTCGATTACAACAGCAACAGCATCATCATCAATGTCCTCCTCATTTCGTGCAGATA
CCCGATATGGAAATGATTTCTTGCGCACCACGTTGCGGTACCGATGCGTATTATCGCGCG
GAAGACAAAAAATTCGCCGAGCGTTGGATGACTGGCTGGGCATGGTTATGTTTCCTGTCA
ACACTCTTCACGCTATTGACATTCTGGGTAGAACCGTGCAGATTTCGTTATCCCGAGAGG
CCGATCATTTTCCTAGCACTCTGTTACAATCTCCTTTCCGTAGCTTACATCGTTCGCGGTG
TGGTTGGTGCTGAAAATCTCAGCTGCATCAGTCAGATTGATGGACCGAGTTACGTTCCAG
TTCGTGATGCTTTGAAGAGCATTCCCTGCACAATTTGGTGGCTGATTAGGTACTATCTAG
GACTGGCCAGCAATATGTGGTGGACGGTGCTGTGCGGTTGTTGGCTGCTGAGCGCCAGG
AACGAATGGAGCAGCGAAGCTCTTCACAACATAGCACCCTATCTTCATGCGACCGCATG
GATTCTCCCAATATTTTTTACAGGAGGAAGTTTACTGTCGCGGAACGTAGTAGCAGACGA
GTTGACTGGACTATGCCAGATTTCTGACGAGACGGCGCTATGGCTAGAAGTGCTACCGCA
CACCGTGCTGCTATTGTTGGGCTGTGTATTTGGCAGCGTCGCTGGAAATGCGTTAATCCG
CGTGCGTAGAGCTGTGCGCTGCGCCGGCCGCAGTGCAACGAAACTAGAGCGCCTGATGA
CGCGGCTGGGTATCTTCGCGCTTTTGTACGCACTGCCGGCGCTGGGCGGCCTTGTTTGCG
TACTGCACGAGGCCTCCGTGAGGCCGCGATGGCAAAAACTGGCGCTGCTGGCCGCACTG
GACTGCCGCGCGGCACAGAATTGCGCCCCGGGCCCGGTGTACCGAGCCGCCGGTCTCGA
AGTTGCCCTTCTCAGGCTCTTCCTGTCCCTCATGGTTGGCGTCACCTCCGGTATGTGGGTA

TGGTCCGGTAAGACCTGCCGTGCCTGGAGCAGATTGCTCGCTACGCCCAGCAAACCCGTC
AGGATACAAAATCCTTTTCAAGGCTTCAAGCAGAACGATAACATTGCTTAA

>frizzled4_Solenopsis_invicta_XM_039457558.1_ncbi
ATGCGTTGGATTCTGTTACCGTTGTTGACGTTGGTGCGCGCAATATGGTGCGCGCACGGC
GCCTGCGAGCCGATCAGAATCGAAATGTGTCGCGGTCTCGGCTACAATGTCACTGTCATG
CCGAATCTAGTTGGTCACGAGATCCAAGGCGATGCTGATTTTACTTTGCAAACGTTCAGC
CCGCTTATTCAATATGGATGCAGTGCGCAATTGCATCTATTTCTGTGCTCGGTGTATGCTC
CAATGTGTACCGAGAAGGTGCCCGCACCTATCGGCCCCTGTCGAGGCTTGTGCGAGCAA
GTGCGAGCCCGTTGTTTCCCAGTTCTTCAGGGATTTGGCTTCCCCTGGCCCGCCGCGCTGA
ACTGCTCCAAGTTTCCACCGGAAAACAATCATCAACACATGTGCATGGAAGGACCGGGC
GAGCCAGGACCGGCTAATCCTATCCAGGCGGTGAGCACCAGCAACGGACCCTGGGGCTG
CTCCTGGTACGCCAAATCCGGATTATACACCTTCTTAAATCGCTCTGGAAAATGCGCCGC
CGCTTGTGACGCCGATATCCTTTGGTCCCAAAAGGATAAGAGGCTCGCGGAGGCTTGGAT
GGCCGTGTTCGCGACGGTGTGCCTGGTCTCCGTCGCGGTCGCCATTCTGACACTTTTGAA
GCCGCGCAAGCGGCCTATCCTCACCACTACCGCAGAGCGTGCAATAGTCTTCTTGACAAT
CTGTCATGCGGCAGTCGCAATTGGCTACGTAATTCGATTGGCTGCCGGTCGGCTGAACGT
CGCCTGTACTCCCGCGATTCCGTTACATCCGCAGGATCAAGCTGTCTTGGCCCAACAGCA
GCAGCAGCAGCAACAGCAACAATATTTAACGCAAGACGGCCTAGCTAATCCGTATTGTG
CTGTTGTCTTTTTGCTGCTCTATTACTTCGGAAACGCAGCGATCGTTTGGTGGGTCGTGAT
ATGCGCGTGGTGGTGCATAATGGCCAGAAAATGGACGAGGACGGCCGGTAACTACAACA
AGGAGGATAGTTTCGGGCCGCAACAGGGATTCTCAACCGTCGCCGCCGTCGCGGCCTGG
GGCCTTCCCGCCGCGCACACCATCGCCGTGCTGGTCACGAGAGATGTTGACGCCGATGA
GTTAACTGCAAGCTGCTTTGTTGGCCAGCAGAACGCACACTCCCTCCTCGTATTAGTACT
GGCGCCGCAATTCGTCTACCTATCGTTTGGCACGACGTTCCTCTTGGTCGGCTTGGCGAC
GCTGGTGTTACCGCGACGACCGGCGGTGACCCCGACGTCGACGTCGACGTCGTCGTCCTC
GTCGACGGCGGCTGCAGCGGCGGCGGCAGCGGTTCTCACCGCCCTAACGGCGTCCCACG
CAAATCCGCTGATGCGCCCGCAGCGCGAGCTTCCCGGCAAATCGTCCCCAGCCGAGATC
CACCGGCGGCAGAAGCAGTTGCTGACTCGCGTTGGTATCTTCGCCTGCCTCTACGCCAGC
CTGATCGTCTGCCTCGCCAGCACGACCTTCTACGAGTGGTGGGGCAGGGAGACGTGGCTC
CGGGCGCCGGAACCGTCGACGGCGCCGCGCATACCGGCACGACCTCTCCTGCAGTTCTTC
GTCCTGCGGCTGGTGGTGACCCTCGGTGCCGGCATCATGGCCGCCGCGTGGATCTGGTGG
CCAGAGGTGACGAGCGCGTGCCGCAAGCTGCCACACTGCAGGCAGCCGCCGTACAAGTG
CCACCCGGTGCCCGTCGTGCACTGTTACAACGCCGGCACCGCCGGCAACCCCATATCTCA
TCATCAGATTCACCATCTCGCCCATCTCACGCCGCCGCAGCATCCCCTGTTGCATCAGCA
CGCGACGCTAACGAATTCGCAGATACCCCATAGGAATCACAAAAAGCATCGGAAGCATC
GGAAACATTATCATTCCGGTAGCGAAACGCAAGTCTGA

>frizzled9-like_Vespula_vulgaris_XM_050988907.1_ncbi
ATGCCCCTGGTACATTACAACTGTTCGAAACACTTGAGGCTCTTTCTTTGCGCCGTCTTCG
CCCCTGTATGCTCCGAACATGTGGCCATGCAGATACCCGCTTGCAAGTCTCTCTGTCTTTC
GGTTAGGAGAGAGTGCGAGCCGGCACTGACCAGCCTCACTCTTCCTTGGCCGCATATGCT
CGACTGCGATCGATTTTTGGATCGAGGCAACACACTTTGCGTTCAACCACCCGAGGAAAC
TCTCTCGGACGCTGTTCCGTCCGTTCAACAGCAATGGCCAACGCAAGAACAAATTCAGCC
TGTTCAAACGTCCACGGGATTGCAAAATCACCATCAGTGTCCACCTCACTTCGTACAAAC
GCCGGATGTACAAACGATCTCTTGTGCACCGCGTTGCGGTATCGACGCTTATTATCGCGC
CGAGGATAAAAAATTTGCTGAACGATGGATGATAGGATGGGCATGGCTCTGCTTTCTATC
CACGCTTTTCACTCTTTTAACGTTCTGGGTCGAGCCCTCCAGATTCCGTTATCCAGAGAGA
CCCATAGTATTTTTGGCGCTCTGTTACAATTTATTATCGGTAAATTATATCGTCAGAGGTG
CGATCGGTACCGAAATCCTCAGTTGTGTCAGCCAACTCGATGGACCCAGCTATGTGCCGG
TTCACGACGGATTAAAGAGTATTCCTTGTACGAGCTGGTGGATCGTCAAGCATTATCTGA
GTTTAGCCAGTAGCACTTGGTGGGCGATACTGTGCGGTTGTTGGCTTCTTAGCGCCAGAA
ACGAGTGGAGCAGCGAAGCTCTGCACAATATTGCTTCGTATCTACACGGAGTTGCTTGGG
GATTACCGCTCTTTGCTACCGGAGGCAGCTTACTCTCTCGTCAAATAGTTGCCGACGAAC
TTACCGGTCTCTGTCAAATCGCCGATGAATCCGCTTTGTGGCTCGAAGTTTACCACACG

CGATTCTGATCTTTCTCGGTTGCATTTAGCAGCAGTGGCAGGCGGCGCGTTGATTCGCG
TACGACGAGCTATACGTTCGGCCGGTCGTAGCGCGACGAAACTGGAGAGACTGATGACT
CGTCTTGGAATCTTTGCGTTGCTTTACGCCCTGCCAGCCCTTGGTAGCTTAACCTGTATTC
TACACGAATCTTCGACGAGACCTCGCTGGCGAACGCTCGCGCTTCTCGCTGCTCTTGATT
GTCGATCAGCGGAGAACTGTGTACCAGGTCCCGTTTATAGAGCGGCAGGACTCGAAGTG
GCCCTTCTCAGGCCGTTTTTGTCTTTGGTCATTGGCGTGACCTCGGGTATGTGGGTCTGGT
CTGGTAAAACTTGTAGAGCTTGGAGTAAATTACTCGCAGCTCCTACTAAACCTACTAGAT
CTATCCCAACTGCCCAACCTCTGACGCAAAATGTTCTTCGGAATTTTAAAGCCGATGTAA
ATAATCTGCCATGA

>frizzled4_Vespula_vulgaris_XM_051011549.1_ncbi
ATGTGGTGGTTCTTGTTATTGATATTCTTGCAAAGGATGCAACACGTTCGTGGTGCGTGC
GAGCCCATAAGGATAGAAATGTGTCGTGGTTTGGGTTACAACGTGACCGCCATGCCGAA
TTTGGTGGGCCACGAGATTCAAGGAGACGCCGACTTCACCCTACAAACGTTTAGCCCGTT
GATTCAGTACGGATGCAGTGCTCAACTTCATTTATTCCTTTGCTCGGTATACGCACCAATG
TGTACAGAAAAGGTACCATCACCGATCGGTCCTTGCAGAGGTTTATGCGAACAAGTTAG
AGCTAGATGCTTTCCAGTTCTTCAAGGTTTTGGTTTTCCTTGGCCAGCAGCTTTAAATTGT
TCCAAATTTCCACCGGTGAACAATCATCAACATATGTGTATGGAAGGACCAGGTGAACC
AGGTCCTGCTAATCCTATCCAGGCAGTCGGTGCCGGTGGTGGACCTTGGGGCTGTTCGTG
GTACGCCAAGTCTGGCTTATACGTGTTTCTAAATCGTTCTGGAAGATGCGCAGCAGCTTG
CGATGCAGATATACTTTGGTCGCAAAAGGATAAGAAAGCTACGGAAGCTTGGATGGCAG
CATTTGCTACGGTTTGCCTGATTTCCGTCACTATTGCGATCTTATCATTGTTGAAGCCAAG
AAAACAATCTACTGTTATGAGTACAGCTGAGAAAGCAATTACATGTCTCGCCGTTTGTCA
TGGGATCGTTGCAGTAGGTTACGTGATTCGATTAGCTGCTGGCAGATTGACCGTAGCTTG
TACCCCTGCAATTCCGTTACATCCCCAAGATCAAGTGGTTATATCTCAACATCAACAACA
ATATCTGACCCAGGATGGATTAGCTAATCCTTATTGCGCTGTTATCTTTCTCTTGTTATAT
TATTTTGGGAATGCTGCTATCGTATGGTGGGTCATCGTCTGTGCATGGTGGTGCATAGTC
GCGAGAAAATGGTCGCGAACAGCAGGTAATTGCAAGGAGGATAGTTTCGGTCTACAGCA
GGGATTCTCAACCGTGGGAGCGGTTGCAGCATGGGGACTTCCAGCTGCTCATACGATCGC
TGTTTTGGTCACTAGGGATGTCGATGCCGATGAGCTAACGGGAAGTTGCTTCGTAGGACA
ACAAAATGCCCGATCTCTTTTGGTTCTCGTATTAATACCACAATTCGTTTATCTATGTCTG
GGTACAACGTTCCTTCTTGTCGGTATAACGTCTCTTTTATTACCAAGGCCATTGGTCACAC
CAAGCTCCGTTGTAAACGCACTTCAACAACAACAACATCAACAACAACAACAACAACTA
CAACAACAACAACATCCCAATCCATTGATCAGACAAAGAGATTCGTCAAAGTCACCGGG
TGAAATACATAGGAGACATAAAATTTTGTTAACTAGAGTCGGTCTCTTTGCTACATTTTA
TGCAGTGACAATGTTTTGTTTAACCAGTACAACCTTTTACGAATGGTGGGGTCGTGAAAG
ATGGCTCAGAGCACCAGAACCATCGAGTTCACCCAGAGTACCGTCGAAACCTCTCGTTCA
GTTTTTCGTTCTTCGATTAGTAGCTTCTTTAGGAAGTGGCATAGTTACAGCTGCTTGGATT
TGGTGGCCTAATTTAATGAACGTTTGGAGACGTTTACCACCTTGCAAACAACCACCTCAC
AAGTGTCATCCTCATGGAGTACCTGTACCCGTAGTACATTGTTTCAATCCGGGTTCTACA
AGTCCAACGCCACATCAAATTCATCATCTCGGCCATTTGGCTCCACCGCAGCATTCACTT
CTACATCAACATCAAATCGTTAGTAATTCGTCGTCTAGTAGTCATAGAAGTCATAAGAAA
CATAGGAAACATAGAAAGTATCATAGTGGTAGCGAAACACAAGTATAA

Sequences used for Wnt phylogenetic analysis

>Wnt1_Bicyclus_anynana_BANY.1.2.t03758_lepbase
ATGTCGGGTCCGCCCATAATGAAGTGGTTGTGCTTGTTTGTGCTGTTTCTGTGTATGAGGT
GCGAGGCCAACAAGCCGAGGCGAGGCCGAGGCAGCATGTGGTGGGGCATAGCAAAGGC
AGGCGAACCAAATAACTTATCACCCTTGTCTCCAAGTGTCCTATACATGGACCCGGCTGT
TCACGCCACCTTGAGGAGGAAACAGAGAAGGCTAGCGAGGGAGAACCCTGGGGTCCTCG
CAGCAATATCCAAGGGAGCCAGCATGGCTGTGGCCGAATGCCAGCATCAGTTCAAATAC
AGGAGATGGAACTGTTCTACAAGAAATTTTTTGCGAGGGAAGAATCTATTTGGAAAAAT
TGTTGACAGAGGTTGCCGTGAAACCGCCTTCATCTACGCCATCACAAGCGCAGGGGTGA
CGCACGCGGTGTCGCGCGCATGCGCCGAAGGCTCCATCGAGTCCTGCACGTGCGACTATT
CCCATGTGGACCGCTCGCCGCACCGCGCGCGCGCCGCCGCCGCCGCCAACGTGAGGGTC
TGGAAATGGGGCGGCTGCAGCGACAACATCGGCTTCGGCTTCAAGTTCAGCCGAGAGTT
CGTTGACACCGGGGAAAGGGGCAAGACGCTTAGGGAGAAGATGAACTTGCACAACAAT
GAGGCCGGCAGGATGCACGTGCAAACGGAGATGCGCCAGGAGTGCAAGTGCCACGGTA
TGTCTGGGTCCTGCACGGTGAAGACGTGCTGGATGAGGCTGCCGACGTTCCGGTCTGTAG
GCGACGCCCTGAAAGACAGCTTCGACGGGGCGTCGCGGGTCATGATGCCCAATACCGAG
GTGGAGGCGCCGTCGCAGAGGAACGACGCCGCACCTCACAGGGTCCCGCGCCGTGACCG
CTACAGGTTCCAACTTCGGCCGCACAACCCTGACCACAAAACACCCGGGGTCAAGGACC
TTGTATACTTGGAATCTTCACCAGGTTTCTGCGAAAAGAACCCCAGACTGGGCATCCCGG
GTACGCACGGGCGTGCCTGCAACGACACTAGCATCGGCGTCGACGGTTGCGACCTGATG
TGCTGCGGGCGCGGCTACCGGACCGAGACCATGTTCGTAGTGGAACGATGCAACTGCAC
GTTCCACTGGTGCTGCGAGGTCAAATGCAAATTGTGTCGCACGGAAAAGGTAGTTAACA
CGTGTTTATAG

>Wnt5_Bicyclus_anynana_BANY.1.2.t00712_lepbase
ATGGGCCTCATAGAGTTCAAGGAATGGTCGAATCAGACGGTGGTATTCTCCGGAAAATC
ACGGGTGTGTGCCCACCTGCGCGGCCTGACTCCGGGCCAGCGGCGCGTGTGCCGCCGAC
ACAAGGACCACATGCCGGCGGTCGGCGACGGCGTGCTGCGCGGCATCAGGGAGTGCCAG
CACCAGTTCCGGCACAAGCGGTGGAACTGCACCGTCACTGCTGACGAAACTGTCTTTGGG
CCCTTGACATTGATTGCGTCAAGAGAAACCGCGTTCACCCACGCGATCACAGCGGCGGG
CGTGTCTCTGGAGATCAGCCGCGCGTGTCGCGACGGACGGCTGGCGTCGTGCGGTTGCA
GCCGCGCGGCTCGCCCGCGGCATTTGCACTCCGACTGGGTGTGGGGCGGCTGTGGGGAC
AACCTCGAGTATGGATACAAATTCACGGAAGGTTTCGTGGACATTCGGGAAAGGGAACG
CAAAGTGAAAAGAGGCAGCCGCGAACAGGGCAGGCAGCTTATGAATAGACACAACAAT
GAAGCTGGCAGGCGGGCTGTCATAAAGAAGTCGCGAGTCACGTGCAAGTGCCACGGCGT
GTCCGGCTCCTGCAGCCTCATCACCTGCTGGCAACAGTTGGCCACATTCAGAGAGATCGG
AGACTATCTACGCGATAAATATGAAGGTGCCACGGAGGTGAAGGTGTCTCGACGTGGAA
AACTGAGGCTGAGCAACCCAAACTACAGTCTACCCACTGCGCAAGATCTCGTATATTTGG
AGGACTCACCAAATTACTGCATTCGAAATATTTCTTTGGGTGCACCTGGAACAATGGGCA
GAGAGTGCAACAAAACTTCAGCAGGCATTGACGGGTGCTCACTAATGTGCTGCGGACGG
GGCTACAACACCAAGAAGATTGTAGTTAAAGAGAGATGCGAGTGCAAGTTCCACTGGTG
CTGCCGTGTGGATTGCAATACTTGTGTTACCACTATGGAAGTGTATACTTGTAAATAA

>Wnt6_Bicyclus_anynana_BANY.1.2.t03759_lepbase
ATGGATAATACAAGAGAGACGGGGTTTGTGAACGCGATCACTGCAGCCGGAGTGACATA
CGCGATCACCCGCGCCTGTACTGCGGGCTCACTGCTCGAGTGCTCATGTGAAAAGGGTGT
ACCAAAACCGCGTCGTGGAAGAACTCAAACGCCCCAGCCCCCAGCACCAACTCAGACAG
AGCAGTGGCAGTGGGGCGGATGCAGTGACAACGTCCGCTTCGGCCTGCAGAAGTCCAGG
GAATTCATGGACAGTAGATACAGGAAGAGGAGCGACATCAAAACGATGATAAAGCTGC
ATAACCACAACGCTGGGAGGTTGGCAATCAAAAATAACATGAAAGTAGACTGTAAATGT
CACGGCCTATCTGGCTCATGCACACTGCGAACTTGTTGGTGGAGAATGCCCACCTTTAGA
GAAGTGGGGGACCGATTGAGAGACAACTTTGAAGGTGCTGCTAAGGTGATCTCAAGTAA
TGATGGCGACAGTTTTATGCCCGAAAGTCCTAACATCAAGCGACCTGGGAAAAAAGATA
TCATATACTCTGAAGAATCACCCGATTTCTGCGGACCTAACATGAAGACAGGGTCACTCG

GCACTGAAGGGCGCCAGTGCAATATAAGTTCTGCGGGAACTGACAGTTGCGATCAACTT
TGTTGTAGAAGAGGGTACATACAAACATCTATAAAGGAGGCTGAAAATTGCAATTGTCA
ATTTAAGTGGTGTTGCGAAGTCATTTGTAAAACATGCTATGTGAAGCGAGACATACAAAC
GTGCCTTTAA

>Wnt7_Bicyclus_anynana_BANY.1.2.t05290_lepbase
ATGGTTAGTTGCATAATATCAAGTACGTGCGTTGAAAAATATCCCAACCTTAATGCTAAC
TTCAAGCAGAGGCGTCCCAAAAACGTCCTTTACGATCCAAACGATGACATGACCGACAA
TGCTTCCCGGGCAGCACAATCAGAAACTACACAGCGTCTTCGCCGGCGAAGACGAGGTC
CCCGATATATAACGAACAGCGCGTCGGGCGGAGTGTCTCTGGGCGCTCACCTGGTGTGC
GCGCGCGTGGCCGGCCTCACCGACAAGCAGCGCGCCATGTGCCGCGCCTCGCCCGCCGC
CATCGCCGCTGTGGGCGACGGACTGAGAATGGCGTACACAGAGTGTCGGTCTCAGCTGG
GCGGCTACCGGTGGAACTGCACCGGCATCGGAGACGGGAACGACTTCGGACATGTCATG
CCTTTAGCGACCCGGGAAGCAGCGTTCACGTACGCGATAACGTCGGCCGGAGTCACTCA
CGCGCTGAGCACGGCGTGCGCGCGCGGCGACCTGCCCGCTTGTGGCTGCTCCTCCAACAG
ACGGCGGTCTCCAAGTCCAACGGAGCAGTTCCAGTGGGGCGGGTGCGGGGAGGCGGCGT
ACGGCGCGCGGTTCGCGAGACGCTTCCTGGACTCGCGGGAGATGGAGGCAGACGCGCGC
AGCCTCATGAATCTGCACAACAACCGCGTCGGCAGGAAGATGGTAAAAGACTTAGTGCG
ACGAGAGTGCAAGTGTCACGGCGTGTCGGGCTCCTGCGCCTTGCGAACTTGCTGGCGCGC
GCTCCCGCCCTTCCGCGCGGTGGGCGCCGCCCTACGCGACAAGTACCACCGAGCGAGGC
TCGTCGCACCGCACCCGCCGCCGGCCACGCACGCGCCACAGACGCATCTCGTCATACGC
AGGTCAAGACAAAACGCTGGTGTGGGCAGACAACCCCGCAAGTCCGAGCTAGTGTTCCT
GGACCCGTCGCCGTCGTACTGTGAACCTGATCCTGTCTCTGGTTCCATGGGCACGCATGG
GCGACATTGCAACAGGACCTCAAGGGGAGAGGAAGGCTGTGAAACTCTTTGCTGTGGTC
GCGGCTACAATACTGTGCGGACCGAAGAAGAAACAAAATGTAATTGCCGTTTCCATTGG
TGTTGCCGTGTTTCGTGCGATAAGTGCATCACTCGTACCGAGTTACACGTTTTGCAGTTCC
AACGATTCATTGCTTTGGCGAAGTGCAGAGAACGGACAAATATGTACAAGGCAAAGACA
CCAGCTCCTGCCCCTTCAAAGCCTGCAGCCCCACGTGGCCGAGGCAGACGGAGCATCTTC
AAGAGGCAGCCATTAAAAGCTCCCACTGCCACAGCAACTGTAGTCACAAGTGACTCTTT
GTTTTTTAAGGGCTCATACATGCAAGTTGGAGATATAGTATCTATGTTAGATGTAGACGG
TGGCACATTCTATGCTCAGATCAGAGGTTTCCTCACCGACCAGTACTGTGAGAAGAGTGC
GGTAGTGACCTGGCTGTTGCCTACTAAAGCTAGCCCTCCGCCGGAGAAGGGATTTGATCC
AGCTACTTACATTATTGGACCAGAAGAAGATCTACCTCGGAAGCTGGAATATATGGAGTT
TGTAATGCATGCACCGTCTGACTATTATAAAGCAAGCAATAGTCCCTACCCACTCACAGA
CAATGAAGTGAACAATTACAATGGATTCATTTGGACAAGCCTTGAACCTAAAGATAGAA
CATAG

>Wnt9_Bicyclus_anynana_nBa.0.1-t07006-RA_lepbase
ATGAAAATCCACACCCCTTTCGGTTTCTACACGACATCGTACCGAAATGCTAAATCGCTT
GGCGTTTCTATATTGGCTTTGAAAAATGTTGAGGGATCTCGACCGGTTCTAGAGAAGACA
GTTGAAGCCTTATTCGCTGACAGATCTGAAGTTATTCATCCAGCCACGTGTAAAGTATTC
ACATCGTCTACACGTCAGTCAAAAATGTGCAAAAGAGAGCCAGGTTTGCCGAATATACT
AGTCAAAGCGAAACAGCAAGCAATTAAGGCTTGTGAAGAGACTTTTCAATACGACAGAT
GGAACTGTTCACTGGTTTTCAATAAGAAGCCGAAAAGGAGTATTTTCAAAAAGATATAC
AGAGAGACTGCGTTTATACACTCTCTTGTGGCAGCTTCTATCACTCACGCAGTAGCAAAA
GGATGTGCTTCTGGTGAGCTATCCAGATGTTCTTGTATCGGTAGTTTTAGAAACGCCTCG
AACGTTCAAATGAGAGGAGGCTGTGGCGATGATTTTAAATTCGGTAAACGATTTGCTAA
GAACTTTCTCGAATGGAAAACAGCGGGGAACGATCAAATAGCGGATGCTTTAAAACAAG
ATGTCAACATAGGTATTGATTCCATAGGCGAACAGTTGAAAGAAGTTTGCAAATGCCAT
GGATTTTCCGGTTCGTGTACAACGAAGACTTGCTGGAAAAGACTGGGTCCTTTCAACTCA
GCTATGGGTTTATTGAAGAAGCATTACCACCACGCGGTGAAGAAAAAACTAGTGAATTT
TACATCTAAAAGAGCTGTTACGATGTAG

>Wnt10_Bicyclus_anynana_BANY.1.2.t03771_lepbase
ATGAGGAAGTTGAAAGTTGCAGTTGGAAAACGCAGAATGCACAGGACGTACCCGGGGG

CGATATATTTTGTGCTGATTGCATTTTTTGAGGTTGTGAGCTCAAGAGACAACATGCTGC
CACACCACCTGAAGCTCAGTTCTACTCTAACTTGTCGACTCATCGGTGGTCTGACCAGAG
AACAGAGATCTGTCTGCCACGACGCGGCCGATACAGCAGCCATCGCCTTCGAGGGTCTG
CAGATGGCGGTCAAGGAGTGCCAGCATCAGTTCCGCTGGCACAGGTGGAACTGCTCCAG
TCTGCTGGTCAAGAGTTCCAATCCTCACGCCAGTGCTATTATGAAGAGAGGATTCCGGGA
AACCGCGTTCCTGTACGCCCTAACAGCGGCAGGAGTAGCTCACGCAGTGGCCCGGGCGT
GCGCCCAGGGCCGGCTCATATCCTGCGGCTGCGACCCCCTGGGGTACCGCGCAGCCCAT
GAGAGGGGCCGCACGAGGACCAACAAGTGGGAGTGGAGTGGCTGCTCCCACAACCTGG
CCTATGGCGTCGAGTTCTCCAAGAAATTCCTCGATGTACGGGAAAAGGTGGACGATCTGC
AGTCGAAGATCAACGTACATAATAACAATGCTGGTAGATCGATTCTATCATCTCACATGG
AGGTGCGGTGCAAGTGCCACGGGCTGTCAGGAAGTTGTCAACTGCGAACGTGTTGGCGC
GCCACGCCCGACTTCAGGGCTGTGGCTTCTACTATTAAGAGACAATACCGCAAAGCTTTA
GTAGTAGCCCAAGAAGAGCTCAATAACAGCCCTTCAGTGTTACGAGGGCGGCCACGAGG
AAGAAGGAGGAGTCGAGCAAGACCTGCACCGAAGTCTAGCTTGCTGTTTTTGAGAAGT
CCCCAAGTTTTTGTGAAGCAGACCCCAAATTTGATTCCGCGGGTACATCAGGAAGAGTCT
GCCGCATCGGAAGGACAACAAGGACTGGATCCTGTGACCTGCTGTGCTGTGGACGAGGA
CACGCCCTCATCAGAAAGTCAAGTATCAAACCATGTAACTGCACCTTTCACTGGTGCTGT
AGAGTCGATTGCCAGAGGT GCCAGGATGATAAATGGATTTCAATTTGCAAGTAA

>Wnt11_Bicyclus_anynana_BANY.1.2.t04594_lepbase
ATGCGTTTATTTATTGTGATTTTTATTTTGTTCTTTGTGTTTTTACCTAAATCTACTAAGGC
GATTCGATGGCTAGCGCTTCATGAGAACGAAGGCAACTGGACGGAGGCGGAGTGCGGCG
ACGCGCGGCGGCGGGGCGAGCTGTGGGCGGGCAGGACGCGCGTGTGCCGCCGCCAGCC
GGCAGCCATGCCGCACGTGGGCAGCCGCCGCGCCCTGGCCCGCGCAGCCTGCCTCGCCG
CGCACGCCGGCGAGCGCTGGAACTGCAGTTCCATCGATNTCGCGCCGAGATACACGCCC
GACTTACTCACAGGTTCTCGAAGCAAGCCTTACGTTGTATGCGATGTCGGCGCGGCGCTT
GCGTGGTCAGTTTTGAGCGCAGCGCGTGCGCGTCGGGGCGCGCTGGCCGCGTGCTCGTGC
GCCGCCCCGCCGCGTGTCCGCCGCGCCCGCCCGCGCCAGGGCCGCGCGCCCGAGCCGCA
CGCGCGGTTCAAAGTGGGGCGGACTGCGGAGACAAACTAATTCAGTGCGGCTGAGAGAT
TCGCGAAACAATTCTTGGACACACACGAGATAGACGTGCGAGACGGTAGAATAGAAGAC
GATATCATCGAGTGGGAGCCGACCACCGAGAAGACCACCACCCTGGAGCCGACCACGAT
GCCTCCCGTCGTCATATTGGTGGACGACCAGCCGGCGCCGCCGAACACCACCGCACCTCC
GAGGAAAAAAGGTCGACGGGGACGCAAACGTTTGCCGAGGTCACCGAGACGAGGACGG
CCCACAAGGAAAAGATTCAGATCCAGATACGACTACGATAACGAGAAAGAATTTGAACA
TCGAAACATAGAGTACCGCATGGCGGCCGACGACCCGCGCTTCGATCCTCAGGTAGACC
TGCACACTCGGCTGCTACATCTGCGGCCGCTCATTGCTGCTGCCAACCTCATCAATAGCC
GCTTCGGAAGAAAGGTGGTATCGCAAGGCATGCGCACCAAGTGCACATGTCACGGCGTG
TCGGGCTCGTGCTCCGTGCGCACCTGCTGGCGCGCGCTGACGCCGCTGGCGCGCGCGGCC
GAGGCCCTGGCGCATGAAGCCGCGCGCGCTGCTCCCCTTGCGCCGCGCACCCCGCCGCG
CCGCCACCGCCGCACGAAGACGCGGCTGCGCTACGTCACGCCCAGCCCAGATTACTGCG
AGCCCGATCCCGCCGCTGGTTCATTGGGCACACACGGCAGGAAGTGCAACGCGACGCTA
GGCGGGGCAGCGGGCGGGTGTGGGCGGCTGTGCTGCGGGCGCGGGCGGCGCGCGGTGC
GCTCGGCGCGGCTGGAGCGCTGCCGTTGCCGCTACCACTGGTGCTGCCGCGTCGACTGCC
AGC TGTGCCGCGTTACCAGCGAGGACCACTATTGCAACTAG

>Wnt1_Drosophila_melanogaster_FBgn0284084_flybase
ATGGATATCAGCTATATCTTCGTCATCTGCCTGATGGCCCTGTGCAGCGGCGGCAGCAGT
CTCAGCCAAGTCGAGGGCAAACAGAAATCCGGAAGGGGCCGGGGCTCCATGTGGTGGG
GCATTGCCAAGGTCGGCGAACCCAACAACATTACGCCCATCATGTACATGGACCCAGCG
ATCCACTCTACGTTGAGAAGGAAACAGCGACGCCTGGTCAGGGACAATCCCGGTGTACT
GGGAGCCCTGGTCAAGGGCGCCAACTTGGCCATTAGCGAGTGCCAACACCAGTTCAGAA
ATCGCCGCTGGAACTGCTCGACGAGAAACTTCTCGAGGGGCAAAAATCTATTCGGCAAA
ATCGTTGATCGAGGCTGCCGAGAGACGAGCTTCATTTACGCAATCACCAGCGCGGCGGT
GACCCACTCGATTGCCAGGGCCTGCAGTGAAGGAACGATAGAGTCCTGCACCTGCGACT
ACAGCCACCAGTCGAGATCTCCACAAGCGAACCACCAGGCGGGCAGTGTGGCCGGCGTG

CGGGATTGGGAGTGGGGCGGCTGCTCCGACAACATCGGATTCGGGTTCAAGTTCTCCCG
GGAATTCGTCGATACCGGCGAGAGGGGTCGCAATCTGCGCGAGAAGATGAATCTGCACA
ACAACGAGGCGGGTCGAGCGCACGTCCAAGCGGAGATGCGACAGGAGTGCAAATGCCA
TGGCATGTCCGGATCGTGTACAGTGAAGACCTGCTGGATGCGACTGGCCAACTTCCGTGT
GATTGGCGACAATCTGAAGGCCCGCTTCGATGGAGCCACCCGCGTGCAAGTGACCAACA
GTCTCCGGGCCACCAACGCTCTGGCCCCAGTTAGTCCGAATGCAGCCGGCTCGAATTCCG
TGGGCTCCAACGGCCTGATTATTCCGCAGTCTGGTCTGGTCTACGGCGAGGAGGAGGAG
CGTATGCTGAACGACCATATGCCGGACATCCTGCTAGAGAACAGCCACCCGATCAGCAA
GATCCATCACCCGAACATGCCGTCGCCCAACAGTTTGCCCCAGGCTGGTCAAAGGGGCG
GACGAAATGGACGTCGTCAGGGACGCAAGCATAATAGATATCACTTCCAACTGAACCCG
CACAATCCCGAGCACAAGCCACCCGGCTCGAAGGACCTTGTCTATCTGGAGCCTTCGCCG
AGCTTCTGCGAGAAGAACCTGCGGCAGGGCATCCTGGGAACCCATGGCCGCCAGTGCAA
TGAGACCTCGCTGGGCGTCGACGGCTGTGGGCTGATGTGCTGTGGGCGTGGCTATCGGCG
AGACGAGGTCGTCGTTGTGGAGCGGTGCGCCTGCACCTTCCACTGGTGCTGCGAGGTGA
AGTGCAAGCTGTGTCGGACCAAAAAGGTCATCTACACGTGTCTGTAA

>Wnt5_Drosophila_melanogaster_FBgn0010194_flybase
ATGAGTTGCTACAGAAAAAGGCACTTTCTATTGTGGCTCTTGCGTGCTGTGTGTATGTTG
CACTTAACCGCGAGAGGGGCATATGCCACAGTTGGGTTGCAAGGAGTGCCGACATGGAT
ATATCTCGGCCTCAAGTCCCCCTTCATCGAGTTTGGCAACCAGGTGGAGCAGCTGGCCAA
TTCCAGCATACCACTGAACATGACCAAGGACGAGCAGGCCAATATGCATCAAGAGGGCC
TACGCAAGCTCGGTACGTTCATAAAGCCAGTGGACCTGCGGGACTCGGAGACTGGCTTC
GTCAAGGCCGATCTCACCAAGAGACTGGTATTCGATAGACCGAACAACATTACATCTCG
CCCTATTCACCCGATACAGGAGGAGATGGATCAGAAGCAGATAATCCTGCTCGACGAGG
ATACCGACGAGAATGGCCTGCCAGCCAGTCTCACCGACGAGGATCGCAAGTTTATAGTG
CCGATGGCGCTCAAGAATATATCGCCCGATCCACGCTGGGCGGCCACTACACCGAGTCC
CTCCGCTTTGCAGCCGAACGCTAAAGCCATCTCGACCATTGTGCCCTCGCCTCTGGCCCA
GGTCGAGGGGGATCCCACGTCCAACATCGATGACCTGAAGAAGCACATACTCTTCTTGC
ACAACATGACCAAGACCAATTCGAACTTCGAGTCGAAATTCGTTAAATTCCCGAGCCTGC
AAAAGGACAAGGCCAAGACATCGGGAGCTGGCGGTTCGCCGCCCAATCCCAAGCGGCCC
CAGCGGCCGATTCATCAGTATTCCGCGCCCATAGCCCCACCAACACCCAAGGTGCCCGCG
CCAGATGGCGGCGGCGTAGGAGGAGCAGCTTACAATCCCGGAGAGCAGCCAATTGGTGG
CTACTATCAGAACGAGGAACTAGCGAATAATCAATCCCTTCTTAAACCAACAGATACCG
ACTCCCATCCAGCGGCCGGCGGTAGCAGCCATGGCCAGAAGAATCCCAGCGAGCCCCAG
GTGATACTGCTCAACGAGACACTCTCCACGGAGACCTCAATCGAAGCGGATCGCAGTCC
ATCGATAAACCAGCCCAAGGCGGGATCGCCTGCGCGCACAACAAAGCGACCACCTTGCC
TGCGCAATCCCGAGTCCCCGAAATGCATACGTCAGCGTCGGCGGGAGGAGCAACAGCGG
CAGCGGGAGCGGGACGAGTGGTTCCGCGGTCAGTCGCAGTACATGCAGCCCCGGTTCGA
GCCGATCATACAGACGATTAACAATACGAAGAGATTTGCCGTATCAATCGAGATTCCAG
ACTCCTTTAAAGTATCCTCCGAGGGATCGGATGGGGAGTTGCTTTCGCGAGTCGAACGCT
CGCAGCCCAGCATTAGTAGTAGTAGTAGTAGCAGTAGTAGCAGTAGTAGGAAAATCATG
CCAGACTATATTAAGGTATCCATGGAGAACAACACATCCGTCACGGATTATTTTAAGCAC
GACGTTGTGATGACATCGGCAGATGTCGCCAGCGATAGGGAATTCCTTATCAAGAACAT
GGAGGAGCACGGAGGCGCTGGCTCCGCGAACAGTCATCACAATGATACGACTCCAACTG
CAGACGCATATTCGGAGACAATCGATCTTAATCCCAATAACTGCTATAGCGCAATAGGTC
TAAGCAACAGCCAAAAGAAGCAATGTGTTAAGCACACCAGCGTGATGCCGGCCATAAGT
CGTGGTGCCCGTGCCGCCATCCAGGAGTGCCAGTTTCAGTTCAAGAATCGCCGCTGGAAC
TGCAGCACAACGAACGACGAGACCGTATTTGGTCCCATGACCAGCCTGGCTGCTCCCGA
AATGGCCTTCATCCACGCCCTGGCCGCGGCCACGGTGACCAGCTTCATAGCTCGCGCCTG
CCGGGATGGCCAACTGGCCTCCTGCAGCTGCTCCCGCGGCAGTCGACCCAAACAGCTCC
ACGACGACTGGAAGTGGGGCGGCTGTGGCGACAACCTGGAGTTCGCCTACAAGTTCGCC
ACGGACTTCATCGATTCGCGGGAGAAGGAAACCAATCGCGAGACGCGTGGCGTTAAGAG
AAAACGCGAGGAGATCAACAAGAATCGCATGCATTCCGATGACACGAATGCTTTTAACA
TAGGTATTAAACGTAACAAAAACGTAGATGCTAAAAACGATACAAGTTTGGTAGTGAGA
AACGTTAGGAAAAGCACTGAGGCTGAAAACAGTCACATACTCAATGAGAACTTTGATCA

GCACCTATTGGAACTAGAGCAGCGCATTACGAAGGAGATACTTACATCCAAGATAGACG
AGGAGGAGATGATTAAGCTGCAGGAGAAGATCAAACAGGAGATTGTCAACACCAAGTTC
TTCAAGGGTGAGCAGCAGCCGCGCAAGAAGAAGCGAAAAAACCAGAGAGCCGCCGCCG
ATGCGCCCGCCTATCCGAGGAACGGCATCAAGGAGAGCTACAAGGATGGCGGCATATTG
CCGCGCAGCACGGCCACTGTCAAGGCCAGGAGCCTGATGAACTTGCACAACAACGAGGC
CGGACGTCGGGCGGTGATCAAGAAGGCCAGGATAACGTGCAAGTGCCACGGCGTGTCCG
GCTCCTGCAGCCTGATCACCTGCTGGCAGCAATTGTCCTCCATCCGGGAGATTGGCGACT
ATCTGCGCGAGAAGTACGAGGGCGCCACCAAGGTGAAGATCAACAAGCGTGGCCGCCTC
CAGATCAAGGACTTGCAATTCAAGGTGCCGACCGCTCACGATCTTATTTACCTAGACGAA
AGTCCCGACTGGTGCCGCAATAGCTATGCGCTGCATTGGCCGGGAACGCACGGACGTGT
GTGCCACAAAAACTCGTCGGGATTGGAGAGCTGTGCCATCCTCTGCTGCGGCCGGGGCT
ATAATACGAAGAACATTATAGTTAACGAACGCTGCAATTGCAAATTTCACTGGTGTTGCC
AGGTTAAATGTGAAGTTTGTACGAAGGTACTCGAGGAG CACACATGTAAATAG

>Wnt6_Drosophila_melanogaster_FBgn0031902_flybase
ATGCGTTTGCTCATGGTAATTGCAATTTTAATATTCGCAATGCCAATGACTGGATTCGGC
TGGGCGGAGGGCACCAACATCCTTCTCGATCCAAATCTAATGTGCAAAAAGACACGTCG
TCTGCGCGGCAAGTTGGCCGAAATCTGTCGGCACGATTCGGCCCTGCTCAAAGAGATCAT
CATCAATGGCATCAACCTGGGCTTCCGCGAATGCGAGTTTCAATTCCGCAACCGCCGGTG
GAACTGCACTGTTCTGCGCAAGAGCATGAGGAAAATCTTAATGCGCGATTCCCGAGAGA
CGGGTTTCGTGAACGCGATCACAGCCGCGGGAGTGACCTACGCCGTGACGAAGGCCTGC
ACGATGGGCCAGCTGGTGGAGTGCTCCTGCGACAAGGCGCATATGCGCAGAAACGGCGG
CCAGCCACAGATGGTGACGGCGGCCACCGCAGAGGCGGCACTGGAGCGGCAGCAACAG
GCGGCGATGCTGCGACAACAAATGCCGCTGCAGGATCAACATCCCAGTCAGCGGCTGAG
TCGCATGAACAACGCCAGCACCATGACCGATATAGCTCCAGTGGAGCATAGAGGCGGGC
GAAATCGCAGGCCCGGCGGAAGGCGGGGGCGCCGCAAGTTCTGGGACAACATTAAGTTT
CCCGAGGGGCAGTGGGAGTGGGGCGGATGCAGTGACAACGTTAACTTTGGCCTTCGTCA
CTCGCGCGTTTTCCTTGACGCCAAGCAAAGGCAAAGGCGTAGCGATCTGGGCACGCTGG
TTAAGTTCCACAATAATAACGCAGGTCGATTGGCCATTCGCGATGCCATGCGGCTGGAGT
GCAAGTGTCACGGGCTTTCCGGCTCCTGCACGGTGAAGACCTGCTGGCTGAAGATGCCTC
CGTTCCGAGAAGTGGCAGGACGACTGCGAGACCGGTATGACAGTGCTAGGAAGGTGACG
TTGCGCAACGACGGGAACAGCTTCATGCCGGAGAGTCCGCACGCGAGGCCAGCGAACAA
GTACCAGTTGGTCTTCGCAGACGATTCGCCCGACTTTTGCACACCCAACTCCAAGACGGG
AGCACTCGGAACTCAGGGCAGGGAGTGCAATGTGACCAGTTCCGGATCGGATCGATGCG
ATCGCTTGTGCTGCAATCGAGGACACACCCGCAGGATTGTGGAGGAGCAAACCAACTGC
AAGTGCGTCTTTAAGTGGTGCTGCGAGGTGACCTGCGAAAAGTGCCTGGAACACCGGGC
GGTCAACACCTGCCTCTGA

>Wnt10_Drosophila_melanogaster_FBgn0031903_flybase
ATGAAAATTTATGCAAATCAAAGCCGAACGATGACTGCGTGGCGAGCAACATCAAAAGG
CCACGAGCAGCAGCAACTGCCGCAGCAGCAGAAGCAGCAGCAAGAAGCAGGAAGCAGC
AGCAGCAGCAACAGCAGCAGCAACAACCTGGTTGCCACACCGGCCACATCGCGCCATTG
CAATTTGCATTTAATTGTTATGATTATCTTGGCCTGCTGCACACGCTGGCTCTATGGCTTG
CCGGATGGTCGTGCCACCTGCCGTTCAGTGCCTGGATTGACCAAGGATCAAGTGGAGCTC
TGCTACAAGGCCAGTGATGTGACGGCGGCAGCTCTCGAAGGACTCGACATGGCCATACG
AGAATGTCAAATTCAGTTTCAATGGCATCGGTGGAACTGTTCGTCGCTGAGCACAAAGA
GCCGCAATCCGCATGCCTCCAGTTTGCTGAAGAAAGGCTACCGAGAGAGTGCGTTCGCCT
TTGCCATCTCGGCTGCCGGGGTGGCCCACAGTGTGGCTCGCGCCTGTAGCCAAGGTCGTT
TGATGTCCTGCGGCTGCGACCCCACCATCAATCGCAAGACGCTGAACAAGAACCTGCGC
CAGTCTCTGGACAAGGAGAAGAAGCAGTTTCTGCAGTACTTGGAGACCAATCAGATTCT
AACGCCCGAGGAGGAGAAGAAGTACGAGCGCTCCAAGATCGCCAGTCGCTGGAAGTGG
GGCGGCTGCTCCCACAACATGGACTTTGGGGTCGAGTACTCCAAGCTCTTCCTCGACTGC
CGCGAGAAGGCCGGCGACATTCAGTCGAAGATCAATCTGCACAACAATCACGCCGGCCG
GATAGCCGTCTCCAACAACATGGAGTTCCGGTGCAAATGCCATGGAATGTCCGGCAGCT
GCCAGCTGAAGACGTGCTGGAAGTCCGCGCCCGATTTTCACATTGTGGGCAAGGTGCTG

AAGCACCAGTTCCGCAAGGCCATTCTGGTGGATCAATCGAATCTGGGCAACGGGGAGCC
CGTGGTCGTTTTGAAACGGGCGCGCAATAAGAAATCGAACGGTGGCAGCGGCTCCGGAT
CCACGTCGCCCGATCTGGACAGCACGGATGCATCTGGTGGCCACGATGATGGAGGGACA
GGTGACTCTGAGACGCGGCGGCATGACGAACTCGGTGTGGAGCGGGGCACGCGGCAACC
GAGCGCCGATAAGAATGCGGCACGAATGGCCCGAAAACTGGAGACATCGCTGTTCTACT
ATCAGCGCTCGCCCAACTTTTGTGAGCGCGATCTGGGAGCTGATATACAGGGCACCGTGG
GACGCAAGTGCAATCGGAACACCACGACCAGCGACGGATGCACCTCCCTCTGCTGTGGC
CGTGGCCACAGCCAGGTCATCCAGCGGAGGGCGGAGCGGTGTCACTGTAAATTCCAATG
GTGCTGTAATGTGGAGTGCGAGGAGTGCCACGTAGAGGAATGGATTAGCATATGCAATT
AA

>Wnt1_Papilio_machaon_ XM_014507234.2_ncbi
ATGAAGTGTCTGTGGTTGTTTGTGCTCATTGTTCTGTGTACTCGGTGCGAAGCCGCCAAC
AAGCCAAGGAGAGGACGCGGCAGTATGTGGTGGGGCATAGCGAAAGCAGGGGAGCCAA
ACAACATATCTCCGATGTCACCTGGCGTCCTATACATGGACGCTGGGGTCCACTCCACAT
TGAGGCGGAAGCAGCGGCGGCTCGCCAAAGAGAATCCGGGAGTGTTAACAGCTGTCGCG
AAAGGCGCCAACATGGCGGTCGCTGAGTGCCAACATCAGTTCAAATATAGAAGATGGAA
CTGCACCACGAGGAATTTCTTAAGAGGGAAGAATTTATTTGGAAAAATTGTTGATAGAG
GATGCCGTGAGACGGCGTTCATATACGCCATAACGAGCGCCGGGGTGACGCACGCGGTG
TCGCGCGCCTGCGCCGAGGGCTCCATCGAGTCGTGCACGTGCGACTACTCGCACTTGGAG
CGCACGCCGCATCGCACCCGCGCCGCCGCTGCCGCCAATGTCCGCGTCTGGAAGTGGGG
CGGCTGCAGCGACAATATCGGCTTCGGCTTCAGGTTCAGTCGGGAGTTCGTCGATACCGG
CGAGCGCGGCAAGACCTTGAGGGAAAAGATGAACCTGCACAATAACGAAGCCGGCAGA
GCGCATGTCCAGTCAGAGATGCGTCAAGAATGTAAGTGCCATGGAATGTCTGGATCGTG
CACAGTGAAGACTTGCTGGATGCGCTTACCGAGTTTCCGTTCCGTTGGCGATGCGTTAAA
AGACCGTTTCGATGGTGCATCAAGAGTTTTGATGAGCAACTCTGATGTCGAAACTCCCGT
ACTAAGGAATGATCCTGGAGCGCACAGGATGCCGAGAAGGGATCGATACAGATTCCAAC
TCCGGCCGTATAACCCCGACCACAAAGCCCCAGGTGCGAAAGATCTCGTGTATTTGGAGT
CCTCGCCGGGCTTCTGCGAGAAGAATCCTCGGCTGGGCATTCCCGGCACGCATGGGCGC
GCCTGCAATGACACGAGCATTGGCGTTGACGGTTGCGACCTTATGTGCTGCGGCCGCGGC
TACAAAACCGAGACCAAGTTTGTGGTGGAGAGGTGCAATTGTACCTTTCACTGGTGCTGT
GAAGTTAAATGTAAAACGTGTCGTACGGAAAAAGTGGTCCATACATGTTTATAG
TTGTCGGTACAAACGAGATCATCATGTCTGCAACTAG

>Wnt5b_Papilio_machaon_ XM_045684312.1_ncbi
ATGGGTCTACTAGAATTCGAAGAGTGGTCGAATCAGGACTTATTTTCTGGTCGATCTCGC
ATGTGCTCCAAACTGCACGGCTTGACCCCTGGTCAACGACGAGTCTGTCGGCGGTACCAG
GACCACATGCCTGCGGTGGGGGCTGGTGCAAAGCAAGGCATCACTGAATGTCAGTACCA
ATTCAGAAGTCGACGGTGGAACTGCACGGTGACCGGTGATGAAACAGTGTTCGGACCTT
TGACGTTGATTGCATCAAGAGAGACAGCGTTTACAAACGCGATAACCTCAGCGGGTGTA
TCTCTGGCGATATCACGCGCGTGTCGGGACGGCCGGCTGGCGAGCTGTGGCTGCAGTCGT
GCAGCCCGGCCTAAGAACCTGCACGATGACTGGGTGTGGGGCGGCTGTGGAGACAATCT
GCAGTATGGATACAAGTTCACCGAAGGTTTTGTGGACATTCGTGAACGAGAGAGAAAGG
TGAAGAGAGGCAGCCGGGAGCAAGGCAGACAACTGATGAATAGACATAACAATGAGGC
TGGAAGACGGGCAGTCATAAAGAAGTCAAGGGTGACGTGTAAATGCCACGGCGTGTCGG
GATCTTGCAGCCTCATTACGTGCTGGCAACAACTACCGTCGTTCAGAGAGATCGGTGATT
ATCTGAGAGATAAATATGAGGGCGCCACAGAAGTAAAAGTATCGAGACGCGGTAAACTA
CGACTTGGCAATCCACATTACAGTCTTCCCACTGCTAATGACTTGGTCTACTTAGAGGAA
TCGCCAAACTATTGTATCAAGAATGAAACACTCGGTTCCCCCGGCACAACAGGTCGTGCG
TGTAACAGGACGTCGGCGGGTATGGACGGGTGTGCGCTGCTGTGTTGTGGCCGCGGCTA
CAACACTAAGAAAATCGTAATCCGGGAGCGCTGTGAGTGCAAGTTCCACTGGTGCTGCA
GAGTTGATTGTAATACATGCGTACGGACAGT CGAATTATATACCTGCAAATAA

>Wnt6_Papilio_machaon_XM_045678092.1_ncbi
ATGCGGCCGGTGGTCATCGCCGCATGTTTGCTGCTGCTAGCGCCCATCACCGACTCCTGG

TGGGCGTCCGGCAGCGCAGTGATTCTCGACCCGCGAATGGTTTGCAAGAAAAATCGCCG
AACGAAAGGACGTTTGGCGCAAATTTGCAAAAACGAAACGGGTTTGTTGAAGGAAATCA
CGAAGGGCGTTACGCTCGGGGCAACGGAGTGCGCCTTTCAGTTTAGAAACCGTCGGTGG
AACTGCACCACACAGAGACGCAGCATGAGGAAAATATTATTGAGAGATACTCGAGAAAC
GGGCTTCGTGAATGCGATCACCGCCGCTGGGGTGACATACGCCATCACACGAGCCTGCA
CTGCGGGAGCCTTGCTAGAGTGTTCTTGTGAAAAGGAAGTACCCAAACCACGTCGTGGA
AAGGTTACGCCGGTACCCATTCCTCCTTCCCCGGTGGAAACGGAGCGATGGCAATGGGG
AGGCTGCAGTGATAACATACGCTTCGGCCTGATGAAGTCCAGGGAATTTATGGACAGTC
GTTACAAAAAACGAAGTGATAACATAAAGACTTTGATAAAACTCCACAACCATAACGCT
GGGAGGTTGGCGATCAAAAATAACATGAGGGTAGAATGCAAATGTCATGGACTGTCAGG
TTCATGTACTCTCCGCACCTGCTGGTGGAGAATGCCTGCTTTTAGGGAAATTGGAAATCG
ACTCCGTGACAGATTTGAAGGAGCTTCAAAGGTAATATCGAACAACAATGGGGAAGGTT
TTATGCCAGAGAGTCCCAACCTAAAGAGTCCTGGCAAGAAAGATATAATTTACTCCGAG
GAATCGCCAGATTTTTGTACACCTAACATGAAGACTGGATCTTTAGGTACAGAAGGTCGT
CAGTGCAACATCAGTTCTGCCGGAACGGATAGCTGTGACCAACTTTGTTGCAGACGAGG
CTACAAACAAACTACAGTAAAAGAGTCAGAAAACTGTAATTGCCAATTTAAATGGTGTT
GCGAAGTAATTTGTCAAACTTGTTACATTAAGCGAGATATACAA ACGTGCCTATAA

>Wnt7b_Papilio_machaon_XM_014506034.2_ncbi
ATGTTGGAATGCCTGCCCCGGTGGAGCGCGGTACGCAACAATAGTGCTCGTGACATCGG
AAAGAGAACACGATTGTTTTTTGTTCTTCTGCCCCTCTTCTTGCTTCTGTGTGTACGAAGC
AGTGAATCGGGCGGAGTGTCGTTGGGAGCACATCTTGTGTGCGCAAGAGTCGCCGGATT
AACAGATAAGCAACGAGCAATGTGCAGATCTTCACCAGCCGCTATAGCCGCTGTTGGAG
ACGGCTTAAGAATGGCGTATGCGGAGTGCCGGGAGCAACTCAGTGGGTACAGATGGAAC
TGTTCAGGAGTTGGAGATGGAAATGACTTTGGACATGTGATGCCTTTAGCGACAAGGGA
AGCAGCTTTCACATACGCGATAACATCTGCGGGCGTGACGCACGCACTAAGCGCGGCGT
GCGCGCGCGGCGACTTGCCAGCCTGCCACTGCAACTCTAACAGGAGAAGAATGACAGGT
CCATCAGAGCAGTTCCAGTGGGGCGGGTGCGGAGAAGCAGCATACGGTGCACGCTTTGC
TCGCCGGTTCCTTGATGCCCGCGAATTGGAGTCTGATGCAAGAAGCCTCATGAACTTGCA
CAACAATAGAGTCGGCAGGAAGGTGAGCGAGATAGTAAAAGACCTAGTGCGTCGCGAG
TGCAAATGCCACGGCGTGTCCGGTTCATGCGCGCTCCGGACGTGCTGGCGAGCACTGCCG
CCATTCCGGACGGTCGCCGCCGCGCTAAGGGAGCGATACCACCGCGCTAAGATGGTCAC
CCTCCACCCGCCGCCGGACACTAATGCACCGCATTCGCATCTCGTTATACGAAGGGCGCG
ACAGAACGCGGGCGTGGGCCGTCAGCCGCGCAAGTCTGAGCTTGTATTCCTGGAGCCTTC
GCCCTCGTACTGCGAGCCCGACACTGCGGCAGGCTCCTTCGGCACGCATGGACGGCATTG
TAACAGGACTTCTAGAGGGGAAAACGGTTGTGAAACTTTATGTTGCGGTCGCGGCTACA
ACACAATACGCACTGTTGAGGAGACAAAATGTCGGTGCCAGTTCCACTGGTGCTGCCGA
GTTACCTGCGACAAATGTATCACTCGTACTGAAACTCATGTTTGCAAATAA

>Wnt9_Papilio_machaon_XM_045679891.1_ncbi
ATGGTTACAGGAACAAAACCAGTATTGCAAAAAACAGTAGAAGCCATTTTTAATGACAG
ATCTAAAACTATATACCCTGGCGCGTGCAAAGTACTCAATTCAACAGCGCGACAAATAA
AAATTTGCAGGAAAGATCCAGGATTACAGATAGTGTTGGCAAACGCAAGGTCTCAAGCG
ATCGATGCGTGCGAACAAAATTTTCAATACGATCGATGGAATTGTTCTTTGTTTTATAAT
AGAAAAGCGAAGAAAAGTATATTTAAGAAAATTTATAGAGAAACTGCATTTGTCCACGC
GTTAGTTGCAGCTTCGTTGACTCACGCTGTTGCTAAAGGATGTGCTTCCGGGGACTTATA
TAGATGTTCATGTTTAAGAAGAAGAAATAATACAACCCATTGGAAAGGAGCTGGATGTG
GGGATGATTTTAAATATGGAAAAAGGTTAATTAGAAATTTTCTCGGTCTTAAAGAAACTG
GTAACGATCAAATAGCAAATATTCTAAAGCAAGATGTTCTTATTGGTGTTAATTCTATTA
AGCAACATCTGAAGGAAGTTTGCAAATGCCACGGATTTTCTGGGTCGTGTACAACAAAG
ACTTGCTGGAGGAGACTTGGGCCTTTCAACTCTGCAATGGGTTTGTTGAAGAAACATTAT
CATCATGCAATTAGAAAGAAAATTGTTAATGTCACAATCGGTCAGCCTGTAACTAAAAAT
ATGCTGGATTTTGATAAATTGGTGTACTTACAGCGTACTCCAAATTTATGTGTAAGCACT
AAAGGAAGGGTTTGTAAGGATAGAAACAACTGTGCCACTTTGTGCTGTGGAAGAGGTTT

TACTATTTCTAAGAAGTCTATTAAAACCAAATGCAGATGTAAGATGGCGAAGTGTTGTTT
GGTCACATGCGATACGT GTGTAGAAGACATAGACGTTTTACATGTAAATAA

>Wnt10b_Papilio_machaon_ XM_014507228.2_ncbi
ATGAAATTTAAAATTTTCACTGGTGAAGATTGCAGGATGTTTCTGAAGTCTTCGGTCACT
ATAGTTTTTCACTGATACTCCTGTCTAATATGGCGAGTTCTCGCGACAACATCCTGCCAT
CGCATTTCAAGCTAAACTCGGCTCTAACGTGTCGTCTAGTCGGAGGACTGACCAGGGAAC
AGAGAGCTGTCTGCAACGAGGAACCAGATGCTGCTGCGATCGCGTTCGAGGGTCTACAA
ATGGCGGTCAAGGAATGTCAACACCAGTTCCGGTGGCATCGATGGAACTGCTCTAGTCTG
ATCGCTAAGAGCACTAATCCTCATGCTAGCGCTATTATGAAGAGAGGTTTTCGTGAATCG
GCGTACCTTTACGCCCTAACATCAGCAGGGGTAGCGCACGCCATCGCCAGGGCGTGTGC
CCAGGGCCGGCTTCTGTCGTGTGGTTGCGATTCCTTAGGGTATCGCACCTCACACGATCC
CAGAGGAAGAACAAGGGCAAACAAATGGGAATGGAGCGGTTGCTCCCATAATCTGGCAT
ACGGCATAGAGTTCACGAAAAAGTTTCTAGATGTCAGAGAACAGGTGGACGATCTGCAG
TCCAAGATTAATGTGCACAATAATAACGCAGGGAGAACGATCCTTTCATCGCACATGGA
AGTGCGATGCAAGTGTCACGGGCTGTCGGGCAGCTGTCAGCTGAGGACCTGCTGGCGCG
CTACCCCAGATTTCCGGGTCGTCGCCTCTACTATCAAAAGACAATATCGGAAAGCACTTC
TGGTGGCACAAGAAGAGCTAAACAACGGTATGTCAGTACTAAGAGGAAGACCGCGTGGT
AAGAGGCGGAGTCGAGCGAAACCAGCGCCAAAAACCAGCCTACTGTTCTTTGAAAAATC
ACCAAGTTTCTGCGACGCAGACCCACGTATGGACTCTTCGGGCACATCTGGCAGAGTTTG
CAGGATCGGGCGCACGTCCCGCACGGGTTCCTGTGACCTGCTCTGCTGCGGACGAGGTCA
CGCCCTAATACGCAGGTCCAGCATCAAACCCTGCAACTGCACCTTTCACTGGTGCTGCAG
AGTCGACTGTGAGAAGTGCCACGACGATAAATGGGTTGCCATGTGCA AATAA

>Wnt11b_Papilio_machaon_ XM_014504890.2_ncbi
ATGCGTTGTTTTTTCGTAATTTTAGTTTTATTCTTTGTGTTTTTACCAAGATCAACTGAGGC
GATTCGATGGTTGGCCTTGCACGAGAGCGACGGCAACTGGACAGAGAGCGAGTGCGGCG
GTGCGCGGCGTGGCGGAGCGCTGTGGGGTGCGCAGGCACGAGCTTGCCGCCGGCAGCCG
GCTGCCATGCCGCACGTGGCAGCCGCTGCTCGCCTCGCCCGGAACACCTGCCTAACGGCG
CACGCCGGCGAACGCTGGAACTGCTCTTCTATCGAGCTGGCGCCCAGGTTCACGCCAGAT
CTACTCACAGGTACTCGTGAACAAGCCTATGTGTACGCGATATCGGCGGCAGCGTTATCG
TGGACGATGGCGCGAGCGTGTGCGGCAGGCACGCTGGCCGCCTGCTCGTGCGCCACGCC
CCCGCGCGCACCGCCCCGCCCGCCGCGCCATGCCGCCCCGCCCGAACCGCACGCCAGCTT
CAAGTGGGGCGGTTGCGGAGATAACTTACAATGGGCTGAACGGTTTGCTAAACAATTCC
TTGACGCTCATGAAATCGACATCAGAGACGGTAGAATTGAAGATGTAATCGAAGTGGAA
ACGACGACAACGGAGAAGCCGAGGACGACGACGGAGACTATCACAGCGCCGCCTGTCG
TGATACTGGTGGACGACGAGCCCGCGCCTGCGCCCGCCGCTAACCTCACCGCCACGCAG
AGGCCACGCAAGGGCCGTCGCGGACGGAAACGGCTGCCCAGGTCTCCGCGACGAGGCA
GGCCCACGAGAAAAAGGTTCAGATCGAGATACGAGTATGAAGACAGAGGGTCTCGCCAC
CGCAACATCGAGTACCGCATGGCGGCGGACGACCCTCGCTTCGACCCGCAAGTGGACCT
GCGCACAAGACTCGACACGCTGCGCCCGCTCATCGCGGCCGCCAACCTCATTAACTTGCG
CTTCGGCAGGAAGGTAGTATCAAGCGGCATGCGGACGAAGTGCACGTGTCACGGCGTGT
CAGGGTCGTGCTCGGTGCGCACGTGTTGGCGCGCGCTGCCCTCGCTGACGCGCGTGGCCG
CGGCGCTGGCGGGCGAGGCGGCGCGCGCTGGCCCGCTCAAGGCGCACGCCCGCCGCCGC
GCGAGGCCTCGCCCCCGACTGCGCTACGTCACGCCCAGCCCCGACTACTGCGAGCCCGA
GCCCGCCGCCGGCTCCCTCGGCACACACGGCAGAAAGTGTAACGCGAGCCTGGGCAGCG
GGGCGGGGGGGTGCGCGCGGCTGTGTTGCGGGCGCGGGCGGCGCGCGGTGCGCTCCAGC
CGACTCGAGCGGTGCCGCTGTCGCTACCACTGGTGCTGCCGCGTCGACTGCCAGCTCTGC
CGCGTCACCACCGAGGAACACTACTGCAACTGA

>Wnt1_Apis_mellifera_ XM_026444306.1_ncbi
ATGCACACCACCCCCATGATTGCATTCGATCGTCAACAGAAGATGAGGCTTTGGGTGATT
ATCTGTGCCCTGGTGGTGGCGATTCACGCGTCCATCGCCGAAATGCCAAGGAACAAGAA
TCGTGGTAGAGGATCGATGTGGTGGGGAATCGCGAAGGCAGGAGAGCCGAACAATTTTT
TGCCAATGTCTCCAGCCTCCCTTCACATGGATCCCACAGTGTACGCGACCTTGAGGAGAA

AGCAACGCAGGTTGGCCAGGGAAAATCCAGGAGTGTTGATGGCCGTGGCCAGGGGCGCG
AATCAAGCGATCGCAGAGTGCCAACATCAGTTTCGCAATCGTCGATGGAATTGTTCCACC
AAGAATTTCCTCCGTGGGAAAAATCTGTTCGGCAAGATCGTGGACAGAGGTTGTCGAGA
GACCGCGTTCATCTACGCCATCACTAGCGCTGCAGTGACTCACAGCATCGCGAGAGCGTG
CAGCGAAGGCAGCATCCAGTCCTGTTCCTGCGACTATACTCACCAATCGCGACCACCATC
CACCACGCGGGATTGGGAATGGGGTGGTTGCTCGGACAACATCGGCTACGGTTTCAAAT
TCTCCCGTGAATTCGTGGACACGGGCGAACGTGGTCGAAATCTACGCGAAAAGATGAAT
CTTCACAATAACGAGGCAGGTAGAGCGCACGTGTCCTCGGAGATGCGGCAGGAGTGCAA
GTGTCACGGCATGTCCGGCTCCTGCACGGTGAAGACCTGCTGGATGAGGCTACCCAACTT
TCGCGTGGTCGGGGACAACCTGAAGGACCGATTCGACGGCGCGTCCAGAGTTATGGTGA
GCAACTCGGATCGGGTGCGTGGCAATGGGAACGCGATCGTGAGCAATTCGGCGAGCAAT
TCGGTGCACGGGCATCGGGAAGGTCTGGGTCGTCGACACCGGTACAACTTCCAGCTGAA
GCCGTACAATCCGGAGCACAAGCCGCCCGGACCGAAGGACCTCGTCTACCTGGAACCCT
CACCCCCGTTCTGCGAGAAGAACCCGAAACTCGGCATTCTCGGCACCCACGGTAGACAG
TGCAACGACACGAGTATCGGTGTCGACGGCTGCGACCTGATGTGCTGCGGCAGAGGCTA
CAAAACGCAAGAGGTGACCGTCGTCGAGAGATGCGCCTGCACGTTTCACTGGTGCTGCG
AGGTCAAGTGTCAGCTCTGCAAAATCAAGAAGACGATACACACGTGTCTCTAG

>Wnt5b_Apis_mellifera_ XM_397473.6_ncbi
ATGGGCATTCAGTTTCTGCTATATCGCAAGATGGCGGCCTGCAGCGGCTTACTCATTATT
CTGCGATTACTTCTCATCGCCGTGGCCTCGACCGTGCCAGGAACATGGATAAATATCGGT
TTGCAACATTATCCACAATTGGACACAGCTCAGATGATGGAGACTTATGACGTAGCCGCG
TCTAGCATTTGTCACGGGTTGAAAGGACTTTCGCAAGGCCAAGGAAAGCTTTGTCAATTA
TCGGTGGATCATATGCCGAGCGTGGCTAAGGGTGCAAAATTTGGGATTCTTGAGTGCCAG
CATCAGTTCCACGACCGAAGATGGAATTGTTCCACTGTCTCTAATGAATCCGTGTTTGGA
CCTATGCTTAGAATAGCGAGTAGAGAAACTGCATTCGTTCATGCAATCACAACTGCAGG
AGTGGTGTATTCAATAAGTCGATCTTGCAGAGACGGACAATTATCTTCGTGTGGTTGTTC
TAGAAGTAGCAGACCCAGAGATCTAAAACGAGATTGGATCTGGGGTGGATGCGGGGATA
ATCTCGAATACGGTTACAAATTTACACAAGCATTTGTTGATGTAAAAGAACGTGAACGA
AGCTTTAAAAGAGGTAGCAGAGAACAAGGTAGAAGTCTGATGAATCTTCATAATAACGA
AGCTGGACGTAGGGCGGTTATAAAACGATCCAAAGTAACATGCAAGTGTCATGGAGTTT
CTGGAAGTTGCAGTTTAATTACTTGCTGGCAGCAACTTGCATCATTTCGAGAAATTGGTG
ATTTTCTTTTAGATAAATACGATGGAGCAACAGAAGTCAGAGTAAACAGACGTGGCCGT
CTATCTATGAGAGATCCAAGATTTTCGTTACCTACAGCGAATGATTTAGTTTATTTGGATG
ACTCTCCAAACTATTGTCTTCCTAATGAAACACTTGGATCATTAGGTACACACGGAAGAA
TTTGCAACAGAACATCGTCTGGCATGGATGGATGTAATCTCCTTTGTTGTGGTAGAGGTT
ATAATACACAAAAATCAACGATCAAAGAAAGATGCGAATGCAAATTTCGTTGGTGTTGT
TTTGTTGAATGCAAAACTTGTGTCAAAAGCGTAGATATTTATACTT GCAAATAA

>Wnt6_Apis_mellifera_ XM_396945.7_ncbi
ATGGAGATGCGCCTCGTTCTCGTGGCGATCTGCCTCCTTCTCGTTACCCCCATCGCCGGCT
CTTTCTGGACCGTTGGAAATCAGGTGGTGATGGATCCGATGCTTATCTGCAAGAAGACTA
GGAGGTTGAGGGGCAAGATGGCGGACATCTGTCGCAAAGAGCCTTCGCTGTTGAAGGAG
ATCGCGAGAGGGGTACAGGTCGGGACCAAAGAATGTCAGTACCAATTTCGAAATCGCAG
ATGGAATTGTACCACGATCAGGAGATCCCTGAGGAAGATCTTGTTACGCGATACCAGGG
AGACAGGTTTCGTGAACGCCATCACCGCGGCCGGGGTAACATACGCGGTCACCAGAGCC
TGCACTATGGGACATCTCGTCGAATGTTCTTGCGACAAGATGACGTCGAAAGGTAACAA
GCTTGGCAAATTGAGCCGCACCGTCGAAATGGAGAAGAGTTTGCCGACCGAGGGCGACT
GGGAGTGGGGAGGTTGCGGCGACAACGTGAAATTCGGCTTCAAGAAGTCGCGAGACTTC
ATGGACGCTCCTTATCGAAAACGCAGCGACATCAAGACTCTGGTCAAGCTGCACAACAA
TAACGCCGGTCGTTTGGCCATCAGAGAGTTTATGAGCACGGAATGCAAGTGTCACGGTCT
CTCCGGATCGTGCACGGTTCGAACCTGTTGGCGGAAAATGCCACCGTTCCGGGACGTGG
GGAACAGGCTGAAAGAGTCGTTCGACGGCGCGGCCAAGGTGATACCGAGCAACGACGG
GCACAGCTTCATCACCGAGGGGCCGACCATCAAGCCGCCGGACAGATTCGATCTGATTT
ACAGCGAGGACTCGCCCGACTTCTGCAAGCCGAACAGAAAAACGGGCTCGCTGGGTACG

CAGGGACGACGATGCAACTCCACCAGTCAGGGAGTGGATGGATGCGAGCTCCTCTGTTG
CGGCAGGGGATACGACACGAGGGTCGTCAAGGAGAAGATCAGTTGCGAATGTAGATTCC
GATGGTGTTGCGAGGTCACTTGCAACACTTGTCTGGTCAAGAAAACCATCAACACGTGTC
GTTAA

>Wnt10b_Apis_mellifera_XM_396944.7_ncbi
ATGCCTCCTTCTCGATCAGGACGCGTCTACGAAGGACCACCGTCTAGGATCGCCGTTAAA
ATCGGACCCACTATCGCCCTGTTGCTGATTCTCTTCGAGAATCGTCGCGCGACCTGCTTA
ATGAGCAATTCCGTGGACGATTGGGTAAGCGGTAATGCCGTGGTCTGCAAAGGTATTCC
AGGGATGACGAAGGAACAGCGCGAGCTATGCCACAGAAATCCGGACGTGACGGTGGCC
GCGATCAAAGGTCTGCAAATGGCGATATCCGAGTGTCAGCATCAATTCATGTGGCACAG
GTGGAATTGTTCATCCTTGACGCCTAGTAGCAGGACGCAGCAGAGCAGCGTCCTCCTTCA
GAGAGGTTACAGGGAGACTGCGTTCGCGTTCGCGATTTCCGCAGCCGGGGTGGCGCACA
GCGTGGCCCGGGCGTGCAGCATGGGACGGTTGCTCTCCTGCGGATGCGACCCGTCGAGTT
ACAAGGGTAAGCCGCCTTCCAAGGCCAGGGGCACGCAATGGAAGTGGGGCGGGTGCTCG
CACAATCTCGACTACGGCATGGAGTTCTCGAGACAGTTCCTAGATACGCGCGAGAGGGC
CGGGGATATACAGTCGACGGTCAATCTTCACAACAATCAAGCTGGCCGCTTGGCGGTGG
CCAGCAATATGCAAGTGCGATGCAAGTGCCACGGTATGTCGGGTTCCTGCGAGCTCAAG
ACTTGCTGGAAAGTGGCGCCGGATTTCCGAATAGTCGGGAAGACGCTCAAGGATCGATT
TCGCAACGCTGTGCTGGTGGCGCAAAGCAACCTGGGCAGCGTGACGCCGTTGACCAGAG
TGAGAGGATCGCGGAGAAGGAGACCGGATCGGCAGAGGCAGAGGAAACATCGCGGTGG
ATCGGGCGGGAATGGGCGCAAGAGGAGGCCTCGGGATCTCGCCAAGCAGCTGTTTTATT
ACCAAAAATCGCCAAACTTTTGCGAGAGAGATCCAAGCGCGGACATCCCTGGAACAGCG
GGGCGTAGATGCAACAAGACCAGCTCAGGTGGGGACGGGTGCGGCAACCTGTGTTGCGG
AAGAGGTTACAACGTGGTGAGACAACGACGGGTCGAGAGGTGCAAATGCAAGTTTCATT
GGTGTTGCATCGTGCAATGTCAGAACTGCACTGTGGAAGAGTGGATCACTGTTTGCAAGT
GA

>Wnt7_Apis_mellifera_XM_016912433.2_ncbi
ATGAAGGGTATCGGCTGTTTGTTACTTAGGGTGGTCGGAGCAATGGTGATCGGGGCTGCC
GTCTGCGGCCGTATTCCCGGCCTGGCGAAGAGTCAACGAGAGCAGTGCAGAAAAGCGCC
GCACGCGATGCCCGCGGTTGGAGAGGGCGCGGAACTGGGACTTCGCGAGTGCCGGCATC
AGTTCAGACATCACCGTTGGAACTGTTCCCACGTGGCGAACGATCAGGTCTTCGGCCACG
TGGTCGTAGTAGGAAGCAGGGAAGCAGCTTTCACGTACGCAATAAGTTCAGCAGGTGTA
ACGTACGCCGTAACCGCAGCTTGCAGCAGGGGCAACATCACCGATTGTGGTTGCGAACC
CACCGTAAGGACGAGAAAGGAGTTGCCACCAAATGGTTGGGAATGGGGCGGTTGCAGTG
CAGACGTCACGTATGGGATGAGGTTCGCGCGTAGGTTTCTGGATGCGAGGGAAGTCGAG
GGTGACGCGAGAAGCCTGATGAATCTTCATAATAATAAAGCTGGTCGAAAGATAGTTAA
AGCTCTCCTGCGAACAGAATGCAAATGTCATGGCGTGTCCGGCTCTTGCACCGTGAGAAC
CTGCTGGCGAACTTTGCCTAGTTTTCGTCAGATCGGCGATGCATTGATGAAAAAGTATTA
CAGAGCCAGGCCTGTTATAGCGATTACTCCACCGCCACCCCCTACGATGCAGAGTTTAGA
AACGTTACACTCGACATCGGCAGAAATGGTGCCGATTTTAGGAAACGATGCGAAAACCC
AAGGGAAACCGAACGATGTTGGAAAGTCCCGACATGATCGACAACCCCCCAAAAAAGC
GACGAAACCAAGAAGGCCGCACTTAGTGTTAAAAAGAACGAAATCGAACGGTGGATCG
AGCGTGGGACAGAAAAGGATTCCAAAGAGAAACGAGTTGGTTTTTCTACAACCATCCCC
TAATTATTGCGAGCCGGATTTGGTGCAAGGAAGCCTGGGAACACAAGGCAGATATTGTA
ATCGTACAAGTAAAGGTACCGATGGATGTGATTTAATGTGCTGTGGACGCGGTTATAATA
CACATCAATTTACAAGAACGTGGCAGTGCAAATGCAAATTTCATTGGTGCTGCCGCGTGC
ATTGTGAAACGTGTACCGAGCGTACAGAGGAATACACGTGCAAATAA

>Wnt9_Danaus_plexippus_XM_032674190.1_ncbi
ATGTTTAAATCTGGTGTTCTGACCTTTCTGATCATTTTTACAGTTTCAATATTCGCCGCGA
AGAATGTAAATGGGTCTCAGCCAGTATTACAAAAGTCTATCGAGGAGCTGTTTACTGACC
GGGCTGATGTCATTTATCCTGATTCCTGTAAGACATTTAGATCCTCAGCCCGACAGTCCA
AAATGTGCAAAAAAGAACCAGGTCTTTCAAATATTATTCTCACGGCCAAGCAACAGGCA

CTCACAGCTTGTGAAGATACATTTAAATATGATAGATGGAATTGTTCGCTTGTTTTAATA
AACGACCGAAAAAAAGTATTTTCAAGAAAATCTATAGAGAGACTGCCTTCGTCCACGCC
TTGATTGCAGCATCTATAGCTCATGCTGTTGCAAGAGGTTGTGCCTCTGGCAACCTATCC
AGATGTGCATGTTTTGGAAGCTTCAAAAATACATCATGGCATGTTCGTGGCTGTGGAGAC
GATTTTAAATTTGGAAAAAGATTCACAAAAAACTTTCTAGAATGGAAACAGGCAGGGTC
TGATCAAATCGCTGAAATTGTTAAACAAGACATAATCGTCGGTATGGACTCTGTTGGCGA
ACAGATGAAAGAGATTTGTAAATGTCACGGATTTTCTGGATCGTGCACCACTAAGACTTG
TTGGAAGCGACTAGGGCCATTTAATTCAGCTATGGGATTATTGAAGAAACATTACCACCA
TGCTGTGAAAAGGAAATTAGTGAATTTTACAACGAAAAGAGCAGTTACGCCGAAAGCGA
GGAAAAGAATGCAACTTGACAAGAATAATTTGATTTACCTACAGAAGACTCCGAATTTA
TGTGTTAGTACAAAGGGAAGAATATGTAAAGATATTCATAATTGTGCTACTTTGTGTTGT
GGAAGAGGTTACGTTACAGGAAAGAAAATAGTTAAGTCTAGATGCAAATGTAAAATGGT
GGACTGTTGTTTCGTTAAATGCGATACTTGTGTTGAAACAGCGGACTTGTTTACTTGCAA
GTGA

>Wnt11b_Danaus_plexippus_ XM_032663556.1_ncbi
ATGCGTTTTTCTGTCGTGATTCTTATTTTTATATTTGTGTTTTTACCAAAATCAACAAAAG
GGATTCGATGGCTAGCGCTGCACGAAAGCGTTGGCAACTGGTCGGAGAGCGACTGTAGC
GAGGCGCGTCGTAGCGGCTGGTTGTACGGGGTGCAGGCGCGTGTGTGCCGCCGGCAGCC
GGCCGCCATGCCGCATGTCGCCGCTGCTGCACGACTCGCTCGAGCGGCGTGTCTCGCAGC
ACACGCTGGCGAGAGATGGAACTGCTCCTCCATTGAACTAGCTCCCAAGTACACCCCTGA
TTTACTTACAGGAACTAGGGAGCAAGCTTACGTGTACGCAATGTCTGCTGCGGCTCTGTC
ATGGTCGGTAGCGAGGGCGTGTGCTGCGGGCGCTCTGGCCGCCTGCTCGTGTGCGGCGCC
GCCCCGAGCACCACCGCGACCTCCCAGACAAGCCGCTCCCACGGAACCCCATGCTAGAT
TCAAGTGGGGAGGATGCGGAGATAATTTTCAGTGGGCCGAAAGATTCGCGAAACAATTT
TTGGACGCTCACGAAATTGATGTCAGAGACGGAAGAGTAGAAGAGGAGACAGCGGAGT
TGGAACCGACCACGGAACGAACGACTACGACTAGCACAGAACCGACAACAGTTCCTCCG
GTGGTGATCCTCGTGGACGACCAGCCGGCGGCGGGGAACACCAGCGTGGCTCCCAGGAG
GAAGGGACGGCGAGGAAGGAAACGACTGCCTCGCTCGCCAAGACGAGGACGGCCCACC
AGAAAAAGATACGACTACGAGGAGGATAAGGAAATACAATACCGAAACATCGAATATC
GCATGGCGGCGGACGACCCGCGGTTCGACCCGCAAGTGGACCTGAACACTCGACTCCAG
TACTTACGACCTCTCATCGCCTCGGCCAATCTCATCAACAACCGCTTCGGCAGAAAGGTG
GTGTCACAAGGGATGCGTACAAAGTGCACGTGTCACGGTGTGTCTGGTTCGTGTTCAGTC
CGCACCTGTTGGCGCTCCTTGTCGTCTTTGTCTCGGGCCGCGGCCGCCTTGTCGCAGCAGT
CCGCCCGCGCTTCCGTGCTTCCCGCACATTCGTCTCCCCGCCGCGCTCACCGGCCTCGGC
GCGCAGCTCGTCTCAGATACGTCACCCCCAGCCCGGACTACTGCGAGCCAGACCCGGCC
GCCGGTTCGCTTGGGACGCATGGAAGACGGTGTAACGCTACCCTGGGGGCGAGCGCGGG
CGGGTGTGGTCGTCTGTGCTGCGGTCGCGGTAGACGCGCGCTGCGCTCGTCGCGACTGGA
GCGCTGCCGTTGCCGCTACCACTGGTGCTGCCGCGTGGACTGTCAGCTGTGCCGCGTCAC
CACCGAAGACCACTACTGCAACTGA

>Wnt7b_Danaus_plexippus_XM_032666072.1_ncbi
ATGTCGGTATGTGTACATCGCGCGAGCCACGTAGTGCGCGCGAGCCTATCAGCTTGGATA
CTTATTCTGTTGTGTTTTGTGTTGCTTGCTGTAAGTGTAACGGGGGCAGCGTCGCTGGGTG
CACACCTCGTCTGTGCGAGAGTATCTGGTTTGACGGACAAGCAAAGAGCTATGTGTCGA
GCCTCGCCAGCCGCGATAGCAGCTGTTGGAGACGGATTAAGAATGGCGTACGCAGAATG
CCGTTCACAACTAAGTGGGTACAGATGGAATTGTTCTGGAGTAGGTAATGGAAACGACT
TTGGACACGTCATGCCTCTAGCTACGCGTGAGGCTGCATTTACTTACGCCATCACATCCG
CGGGGGTCACGCACGCTTTGAGCACGGCGTGCGCTAGAGGGGACTTACCCGCCTGTGGA
TGTTCGTCGAACAGACGTAGACCTTCGAGTCCATCAGAGCAGTTCCAATGGGGAGGTTGT
GGAGAGGCAGCCTATGGTGCGAGATTCGCGCGAAGGTTCCTTGATTCCAGAGAAATGGA
AGCGGACGCCAGAAGTCTCATGAATCTTCACAACAACCGAGTAGGAAGAAAGACAGTGA
AGGACCTAGTTCGACGTGAATGCAAGTGCCATGGCGTATCAGGCTCGTGTGCGCTGCGC
ACCTGCTGGCGGGCGTTACCCCAATTCCACGTGGTGGGCTCTGCTCTAAGGGAGAAGTAC
CAGAAAGCTAGATTCGTTGTACCTCACCCACCAGCTGATACACACGCGCCACAAACACA

TCTTGTTATACGCAGAACGAGACAGAACGCAGGTGTCGGCCGCCAGCCTCGTAAATCCG
ACCTGGTGTTTTGGAGCCGTCGCCAACGTATTGTGAACCCGACGCTCTAGCCGGATCAT
TCGGTACTCACGGACGACACTGCAACAGAACCTCTAGAAGTGAAGAAGGCTGTGAAACT
CTGTGTTGCGGACGCGGTTACAACACAGTGAAGACTCAAGAAGAAACAAAGTGCCACTG
TCGTTTCCATTGGTGTTGTCGAGTGTCGTGCGACAAATGCGTCACTAAGAGCGAAATTCA
TGTTTGCAAATAG

>Wnt5b_Danaus_plexippus_XM_032666013.1_ncbi
ATGGGGTTATCTATCGCACTGATGTTGCTAGTGATTGTTGTGAGCGGCGACGCCAGGCGG
AGAGACACCTGGACTAACGGCAGCTGGTTCAACATGGGTCTCTTGGAGTTTGAGGAATG
GTCGAATCAGACGGAGCCGTTCTCAGACAGGTCCCGGATGTGTTCCAAAATAGTTGGACT
GACCCCGGGACAGCGTCGGATTTGCAGACGACACAAGGACCATATGCCGGCCGTCGGCC
TTGGTGTCAGGAAGGGAATCCAGGAATGTCAGCATCAATTCCGGGATAGAAGGTGGAAC
TGTTCCATCACGCGGGACGAAACCGTATTCGGTCCACTGACGTTGATCGCATCAAGAGAG
ACAGCCTTCACACACGCTATAACAGCAGCTGGTGTAAGCTTATCCCTGTCTCGAGCGTGT
CGCGACGGCACTCTCAGTTCCTGTGGATGCAGCAGAGCTAACAGGCCCAGACACCTGCA
CAAGGACTGGCTCTGGGGAGGGTGTGGAGACGACCTGGAATACGGATACAGATTTACAG
AAGACTTCGTCGACATCAGGGAAAGAGAAAGAAAAGTCAAGAGAGGCAGCAGAGAGCA
GGGCAGGCAGCTCATGAATAGACATAACAACGAGGCCGGGAGACGGGCTGTTATAAAG
AAGTCCCGGGTGACGTGTAAATGTCATGGCGTTTCCGGTTCCTGCAGCCTCATCACGTGC
TGGCAGCAGTTGCCGAGTTTTAGGGAAATAGGTGATTACTTGAGGGATAAATATGAAGG
TGCTACCGAGGTAAGAGTATCGAGACGGGGCAAGCTGCGTCTCGGCGATCCGAACTACA
GTATGCCGACGGCTCAGGATCTCGTTTATTTAGAGGAATCCCCAAATTATTGTATACGCA
ACGAAACTATGGGATCTCTGGGAACGACGGGTCGGGAATGTACCAGGACTTCATCAGGG
CTGGAGGGGTGTGCGCTAATGTGCTGCGGCCGCGGATACAACACACACAGAACCGTCCT
GAGGGAGAGATGCGAGTGTAAATTCCACTGGTGCTGCCGCGTTGATTGCAACACTTGCGT
TAGAACCGTCGAGGTCCACACTTGTAAATAG

>Wnt1_Danaus_plexippus_ XM_032674365.1_ncbi
ATGGCGGGTCCGCCCATCGATATGAAGTGGCTGTGTTATATTGTGATCATTATGTGTCTC
AGGTGCGAAGCCGCTAATAAACCGAGAAGAGGACGCGGCAGCATGTGGTGGGGTATAG
CGAAAGCGGGTGAGCCGAACACCCTTTCGCCTCTTTCACCAGGGGTCCTCTATATGGATC
CGGCAGTCCACGCAACCTTGAGGAGAAAACAACGAAGGCTGGCTAGAGAGAATCCTGG
AGTGCTTGCTGCTGTTGCCAAGGGAGCCAGTATGGCTGTAGCAGAATGCCAACATCAGTT
CAAATACAGACGATGGAACTGTTCTACCAGAAATTTCTTACGAGGCAAAAACATGTTTG
GCAAAATAGTCGATCGAGGTTGTCGGGAAACAGCATTCCTATATGCAATAACCAGTGCT
GGGGTAACCCATGCGGTATCGCGAGCCTGTGCCGAGGGCGCGATCGAATCTTGCACTTGT
GACTATTCCCATGTCGATCGTGCGCCGCATCGCTCCCGAGCTGCAACCGCAGCCAACGTC
CGCGTCTGGAAATGGGGTGGCTGCAGCGACAACATCGGCTTCGGATTCAGATTTAGTAG
AGAATTTGTGGACACCGGCGAACGGGGCAAAACGTTAAGAGAAAAAATGAACCTGCAT
AATAACGAAGCAGGCAGAATGCACGTGCAAACAGAGATGCGGCAGGAATGCAAATGTC
ATGGTATGTCTGGATCTTGTACTGTGAAAACATGCTGGATGAGGCTACCAAGTTTCCGAT
CTGTAGGAGATGCATTGAAAGATCGTTTCGATGGCGCTTCAAGGGTAATGATGCCAAAC
ACAGAAGTTGAGGCACCAGTACAGAGGAATGACGCAGCGCCCCACAGAGTACCCCGGA
GAGATCGATATAGATTCCAACTTCGGCCTCACAATCCAGATCATAAGACTCCTGGGGTGA
AGGATCTGGTGTATTTAGAATCATCGCCAGGATTCTGTGAAAAGAATCCTAGGCTAGGCA
TACCCGGGACGCATGGACGGACCTGCAACGACACCAGCATCGGAGTGGATGGCTGCGAC
TTAATGTGTTGCGGTCGTGGTTACCGCACAGAGACCATGTTTGTTGTGGAACGGTGCAAT
TGTACGTTCCACTGGTGCTGCGACGTCAAATGCAAACTGTGTCGCACAGAAAAAGTAGT
ACATAC GTGTTTATAG

>Wnt6_Danaus_plexippus_XM_032654741.1_ncbi
ATGCGGCCGGTCTTTATAGCCGCCTGCCTACTGCTGCTTGCGCCCATCACCGATTCTTGGT
GGGCGTCTGGCAGCGCGGTGATCCTTGATCCACGAATGGTCTGTAAAAAGAACCGTCGT
GTTCGTGGTCGGTTGGCTTCCATCTGCAAGAACGAGACTGGCCTGCTGAAGGAGATAAG

CCGCGGCGTCACCCTCGGAGCCACGGAGTGCGCTCACCAGTTCAGGAACAGAAGGTGGA
ACTGTACCACACAAAGACGCAGCATGAGGAAAATCCTTATGAGAGATACAAGAGAAACT
GGGTTTGTGAATGCAATAACAGCCGCCGGCGTTACTTACGCTATTACACGGGCGTGTACA
GCTGGTTCCCTCCTTGAATGCTCATGTGAAAAGGAAATTCCAAAACCTAGAAGAGGACG
TGTTACTCAAGTACCCCAGCCGCCATCTCCAGTGCAAAAGGATAAATGGCAATGGGGTG
GTTGTAGCGATAATGTTCGCTTTGGTCTACAGAAATCCAGAGAGTTTATGGACAGTCGAT
ATAGGAAAAAAAGCGATATAAAAACATTGATTAAATTACACAACCACAATGCTGGGAGG
TTGGCAATTAAAAATAATATGAAAGTGGAATGTAAATGTCATGGACTATCAGGGTCTTGC
ACGCTTCGTACATGTTGGTGGAGAATGCCAACGTTTAGAGAGGTGGGCGACCGTTTGAG
AGATAAATTTGAGGGTGCAGCTAAGGTGATTTCAAATAATGACGGTGATAACTTTATGCC
AGAAAGTCCAAATATCAAACGGCCTGGTAAGAAGGATATCATTTACTCTGAAGAATCAC
CCGATTTTTGTACATTTAATATGAAGACTGGATCTCTGGGAACAGAAGGGCGACAGTGTA
ACGTAAGTTCCGCTGGAACCGACAGCTGTGATCAACTTTGTTGTAGGAGGGGATACGTGC
AAAATACCATCAGAGAAGCCGAAAATTGTAATTGTCAATTTAAATGGTGTTGCGAAGTG
ATTTGTGAGACTTGCTACGTTAAAAGAGATATACAAACG TGCCTTTAA

>Wnt10b_Danaus_plexippus_ XM_032674070.1_ncbi
ATGAAAAAGCAGAGAGTCGCTATTGGAGAAAAACACAGAATGCACATGCATCTAGCTGC
CATATATTTTTTGCTAATACTTATCTTCAAAATCGCAAGTTCTAAAGACAACATTCTTCCT
CATCAACTTAAACTCAGTTCTACACTCACTTGTCGTCTTATCGGAGGGCTGACCAGAGAA
CAAAGATCCGTGTGTCACAGCTCTCCAGATACTGCCGCTATTGCTTTAGAAGGTCTACAA
ATGGCGGTTAAAGAATGTCAACATCAATTTCGATGGCATCGCTGGAACTGCTCCAGCCTT
TTGACCAGAAGCACTAATCCACATAATAGTGCTATAATGAAAAGAGGTTTTCGTGAAGCT
GCTTTTCTGTACGCTCTGACAGCGGCAGGGGTGGCACACTCTGTGGCACGCGCATGCGCC
CAAGGACGCCTCATATCCTGTGGCTGCGACCCCCTGGGATACCGCACCACTCACGAGAG
AGGCAGAGCAAGGGTAAACAAATGGGAGTGGAGTGGCTGTTCCCATAACCTGGCCTTCG
GGATCGACTTTTCCAAGAAATTTCTAGACGTGCGCGAGCAAGTTGACGATCTGCAATCAA
AGATCAATGTTCATAATAACAACGCGGGAAGATCGATACTCTCATCTCATATGGAGGTAC
GATGTAAGTGTCACGGGCTGTCTGGGAGCTGTCAATTAAGGACGTGCTGGAGGACCACG
CCCGACTTCAGAATCGTAGCGTCTACAATTAAGCGAGAGTATCGTAAGGCATTAGTCGTG
GCGCAAGAGGAGTTAAACAATAGCCCTTCAGTACTTCGTGGGCGTCCTCGTGGTAGAAG
GCGAAGTCGTGCTAGACCGGCACCGAAAACAAGCCTAGTCTTCTTGGAGAAGTCCCCAA
GTTTCTGCGAAGCAGATCCTAAGATGGATTCCGCGGGTACATCGGGAAGGATTTGTCGTA
TGGGAAGAACCTCTCGTACGGGCTCATGTGATCTGCTCTGTTGCGGTAGAGGTCACGATT
TAATAAGAAAGTCCAGTATCAAACCATGCAATTGCACGTTTCACTGGTGTTGCAGAGTCG
ACTGCCAGAAATGTCAAGATGACAAATGGATTGCTATGTGCAAGTGA

>wntA_Bicyclus_anynana_XM_052886643.1_ncbi
ATGGATGACATAAACAAGATCCCTTCCCCCCATAAACAAGTTGAATGCGCTTCCCCGATG
CCGCAGTCTCTGCACGTGCGGCACTCAAGGAATTTAGCAGCACCGAATAGGCCTGTACA
ATCGTCTAACACCTCTGTGGAAACCTTTACAATACTGCACAAAGAAAGCTGCCATAGATT
AGAGTATCTCGTCGAACGACAAAAGCAATTATGTATGCTTTCTGATAAAATGGTACAGGT
GATACAAACAGGAGCGCAACAGGCAATTGATGAATGTCAGCATCAATTTCGGAATAGCC
GTTGGAACTGTAGTACCGTCGACAATTCCACTGATATATTCGGCGGAGTGCTAAAATTTA
AATCTCGCGAGTCTGCATTCGTCCACGCTCTGTCAGCAGCAGCATTGGCTCACACAGTTG
CTCGCGCGTGCAGTCGGGGCGAACTAAACGAGTGTTCCTGTGACGCTCGTGTTAGAAAG
CGAACGCCGCGGCATTGGCAGTGGGGTGGTTGTTCTGAGGATATAAGATATGGAGAAAA
GTTCAGTCGTGACTTTGTAGATGCTAAAGAAGACAAGGATAATGATGAAGGTCTCATGA
ACTTACATAACAATGAAGCTGGCCGCAGAGCAGTCCGCGGCAGGATGCAGCGCGTGTGC
AAATGCCACGGCATGTCGGGCTCGTGCTCCGTGCGCGTGTGCTGGCGCCGCCTGCCGCAG
CTGCGGCTGGTGGGCGACGTGCTGAGCACCAGATACGAGGGCGCCTCTCATGTTAAGGT
TGTAGAGAGGAAGAGAGGCAAGAATATAAGAAAACTGCGACCGCTGCATCCTGATATAA
AGAAACCGAACAAAACCGATCTAGTCTATCTCGAGGACTCTCCCGATTACTGTGAACCG
AACGACGAGTAA

>wntA_Heliconius_himera_JN944589.1_ncbi
ATGTTATCCGATAAAATGGTACAGGTAATACAGACAGGTGCTCAACAGGCGGTTGAAGA
GTGTCAATACCAATTCCGTAACAGCCGCTGGAACTGCAGCACTGTCGAAAACAGCACTG
ATATATTTGGAGGAGTACTTAAATTTAAATCTCGTGAATCTGCATTCGTGCACGCCCTCTC
AGCCGCGGCACTAGCCCACGCGGTGGCACGGGCGTGTAGTCGCGGGGAGCTCAACGAGT
GTTCCTGCGACGCCCGCGTGAGAAAGCGGACTCCAAGACATTGGCAATGGGGCGGCTGC
TCGGAGGATATAAGATATGGGGAGAAGTTTAGTCGAGATTTTGTGGATGTTAAAGAAGA
GAAAGAAAGCGATGAAGGTATAATGAATTTACACAATAACGAGGCTGGCCGTAGGGCG
GTACGCGGTCGTATGCAGCGTGTATGCAAGTGTCACGGCATGTCGGGGTCTTGTTCAGTA
AGGGTTTGTTGGCGGAGACTACCTCAGTTGAGGGTGGTGGGTGACGCCCTCACCACGAG
ATATGAGGGCGCTTCTCACGTTAAAGTTGTGGAAAGGAAGCGAGGTAAAAACATAAGAA
AGTTACGGCCAATACACATGGATATGAAGAAACCAAATAAAACGGACTTAGTTTATCTA
GAGGACTCCCCGGACTATTGTGAGCCAAAGGAAGAGCTGGACGTGGCGGGCACGCGCGG
GCGCACGTGCAACCGCACGTCGGCGGCGCTGGACGGCTGCCGTCTGCTGTGCTGCGGGC
GCGGCTACCAGACGCGCGTGCGCGACCACGAGGAGAAGTGCCGCTGCCGCTTCGTGTGG
TGCTGCCGCGTGCACTGCGACCTGTGCCGCTCCAAGCGCGACCACCACGTCTGCAACTAG

>wntA_Junonia_coenia_KJ906611.1_ncbi
CCAAGACATTGGCAGTGGGGTGGATGCTCTGAAGATATAAGATACGGTGAAAAGTATAG
CCGAGATTTCGTGGACGTTAAAGAAGATAAGGAGAGTGATGAAGGAATAATGAACCTGC
ATAATAATGAAGCTGGACGCAGGGCGGTGCGCGGGCGCATGCAGCGCGTGTGCAAGTGC
CACGGCATGTCGGGATCGTGCTCGGTGCGCGTGTGCTGGCGCCGCCTGCCGCAGCTGCGC
CTCGTGGGTGACGCGCTCGCCACCCGCTATGAGGGAGCCTCGCATGTTAAGATTGTCGAA
AGGAAAAAAGGTAAAAATATCAGGAAACTACGACCGATACACGCAGACATGAAGAAAC
CAAACAAAACGGATCTAGTTTATCTCGAGGATTCCCCGGATTACTGTGAACCGAACGATG
AGCTCGGTATACTGGGAACGCGAGGAAGAACATGCAATAGGACATCTGCAGGATTGGAC
GGCTGTAGGCTCTTGTGCTGCGGACGAGGATACCAGACCAGGGTCAGGGACCACGAGGT
GAAGTGCCGCTGC

>wntA_Vanessa_cardui_KJ906612.1_ncbi
CCGAGACACTGGCAATGGGGTGGATGTTCTGAGGATATAAGATATGGTGAAAAGTTTAG
CCGGGATTTCGTAGACGTTAAAGAGGACAAAGAAAGCGATGAAGGAATAATGAATCTAC
ATAATAATGAAGCTGGTCGTAGGGCTGTTCGTGGTCGAATGCAACGTGTCTGCAAATGTC
ATGGTATGTCCGGTTCATGTTCAGTACGAGTGTGCTGGCGTCGTCTCCCACAACTAAGGA
TCGTGGGTGACTCTTTAAGTACAAGATACGAGGGCGCTTCGCATGTTAAGATTGTAGAAA
GAAAAAGAGGGAAGAATATCAGGAAATTGAGACCAATACACGCGGATATGAAGAAACC
GAACAAAACTGACTTAGTTTATCTCGAGGATTCCCCTGATTATTGCGAACCTAACGATGA
ACTCGGAATTCTCGGAACTCGTGGAAGAACATGTAACAGAACATCTGCTGGATTAGATG
GCTGTCGACTGCTATGCTGTGGACGCGGATATCAGACCAGAGTCAGAGATCACGAAGAG
AAGTGCCGTTGT
